# Antiviral Activity of Lipophilic Nucleoside Tetraphosphate Compounds

**DOI:** 10.1021/acs.jmedchem.3c02022

**Published:** 2024-02-12

**Authors:** Xiao Jia, Dominique Schols, Chris Meier

**Affiliations:** † Organic Chemistry, Department of Chemistry, Faculty of Mathematics, Informatics and Natural Sciences, Universität Hamburg, Martin-Luther-King-Platz 6, Hamburg D-20146, Germany; ‡ Laboratory of Virology and Chemotherapy, Department of Microbiology and Immunology and Transplantation, Rega Institute for Medical Research, KU Leuven, Herestraat 49, Leuven B-3000, Belgium; § Centre for Structural Systems Biology (CSSB), Hamburg, DESY Campus, Notkestrasse 85, Hamburg D-22607, Germany

## Abstract

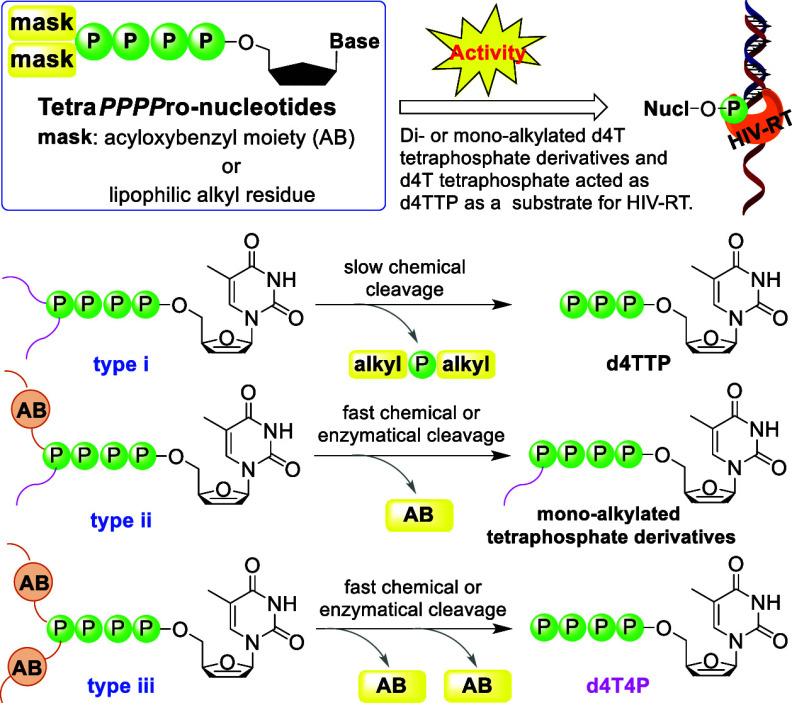

We report on the synthesis and characterization of three types of nucleoside tetraphosphate derivatives **4**–**9** acting as potential prodrugs of d4T nucleotides: (i) the δ-phosph(on)ate is modified by two *hydrolytically stable* alkyl residues **4** and **5**; (ii) the δ-phosph(on)ate is esterified covalently by one *biodegradable* acyloxybenzyl moiety and a *nonbioreversible* moiety **6** and **7**; or (iii) the δ-phosphate of nucleoside tetraphosphate is masked by two *biodegradable* prodrug groups **8** and **9**. We were able to prove the efficient release of d4T triphosphate (d4TTP, (i)), δ-monoalkylated d4T tetraphosphates (**20** and **24**, (ii)), and d4T tetraphosphate (d4T4P, (iii)), respectively, by chemical or enzymatic processes. Surprisingly, δ-dialkylated d4T tetraphosphates, δ-monoalkylated d4T tetraphosphates, and d4T4P were substrates for HIV-RT. Remarkably, the antiviral activity of Tetra*PPPP*ro-prodrug **7** was improved by 7700-fold (SI 5700) as compared to the parent d4T in CEM/TK^–^ cells, denoting a successful cell membrane passage of these lipophilic prodrugs and an intracellular delivery of the nucleotide metabolites.

## 1 Introduction

Clinically approved nucleoside analogues [nucleoside reverse transcriptase inhibitors (NRTIs)^1^ and RNA-dependent RNA polymerase (RdRp) inhibitors^4^] play a pivotal role in drug therapy due to their ability to tackle virus infections (e.g., HIV, hepatitis B and C viruses, and SARS-CoV-2).^2−8^ Among them, nucleoside analogues require three successive enzyme-mediated phosphorylation steps to form the corresponding bioactive triphosphorylated NRTI metabolites to interfere in the metabolic pathway fundamental to aberrant cellular replication.^5,8−13^ However, nucleoside analogue activity may also be limited by poor cellular uptake, rapid catabolism, the release of toxic byproducts, or resistance development.^14−20^ To overcome at least some of these issues, several prodrug strategies such as nucleoside monophosphate prodrugs (SATE-,^21,22^ bisPOM-,^23^ phosphoramidate nucleotides,^24,25^ and *cyclo*Sal^26−28^), and nucleoside diphosphate prodrugs (Di*PP*ro-concept),^29−42^ allowing intracellular delivery of nucleoside monophosphates (NMP) and nucleoside diphosphates (NDP), respectively, have been developed over the past decades.

In 2015, we reported on the first examples of the bioreversible protection of nucleoside triphosphate analogues **1** (NTP, Tri*PPP*ro-approach).^43^ In that approach, two acyloxybenzyl (AB; ester) moieties and/or alkoxycarbonyloxybenzyl (ACB; carbonate) moieties were attached to the γ-phosphate of the NTP.^43−48^ All Tri*PPP*ro-compounds **1** were rapidly hydrolyzed in CEM/0 cell extracts. Moreover, intracellular delivery was proven using a fluorescent nucleoside analogue triphosphate (ddBCNATP).^44^ Subsequently, the second generation of Tri*PPP*ro-prodrugs **2**, comprising an *enzyme-cleavable* AB- or ACB-prodrug moiety in addition to a *nonbioreversible*, *hydrolytically stable* alkyl residue at the γ-phosphate or γ-phosphonate group, was disclosed.^49−54^ In the case of γ-(AB; alkyl)-d4TTPs **2a**(49) and γ-(AB)-γ-C-(alkyl)-d4TTPs **2b**,^50^ the cleavage of the phenyl ester moiety within the masking units led to the formation of γ-(alkylphosphate)-d4TDPs and γ-C-(alkylphosphonate)-d4TDPs, respectively. Interestingly, such compounds still were substrates for HIV-RT. Recently, a potential new generation of γ-bis-alkyl-phosph(on)ate-modified nucleoside analogues **3**(55) was discovered that comprised two different alkyl residues at the γ-phosphate group or γ-phosphonate group, respectively. Previous studies demonstrated that γ-phosph(on)ate-modified-d4TDPs **3**(55) (γ-(alkyl-C4; alkyl-C18)-d4TTP: EC_50_ = 0.032 μM/HIV-2) was markedly more antivirally potent against HIV-2 in thymidine kinase-deficient cell cultures (CEM/TK^–^ cells) as compared to the Tri*PPP*ro-prodrugs **1**(43) [γ-(AB-C9; AB-C9)-d4TTP **1a**: EC_50_ = 0.29 μM/HIV-2, Table 2]. In addition, the initial cleavage step in the hydrolysis mechanism proceeded differently from the published cleavage pathway for Tri*PPP*ro-prodrugs **1** and **2**. The delivery of d4TDP (for the Tri*PPP*ro-compounds **3**) rather than d4TTP (for the Tri*PPP*ro-prodrugs **1**) was shown in CEM/0 cell extracts, which was probably due to chemical phosphoroanhydride bond cleavage between the β-phosphate and the γ-phosph(on)ates. Interestingly, the double-alkylated Tri*PPP*ro-compounds **3** were accepted by HIV reverse transcriptase (HIV-RT) as a substrate in primer extension assays.^55^

**Figure 1:**
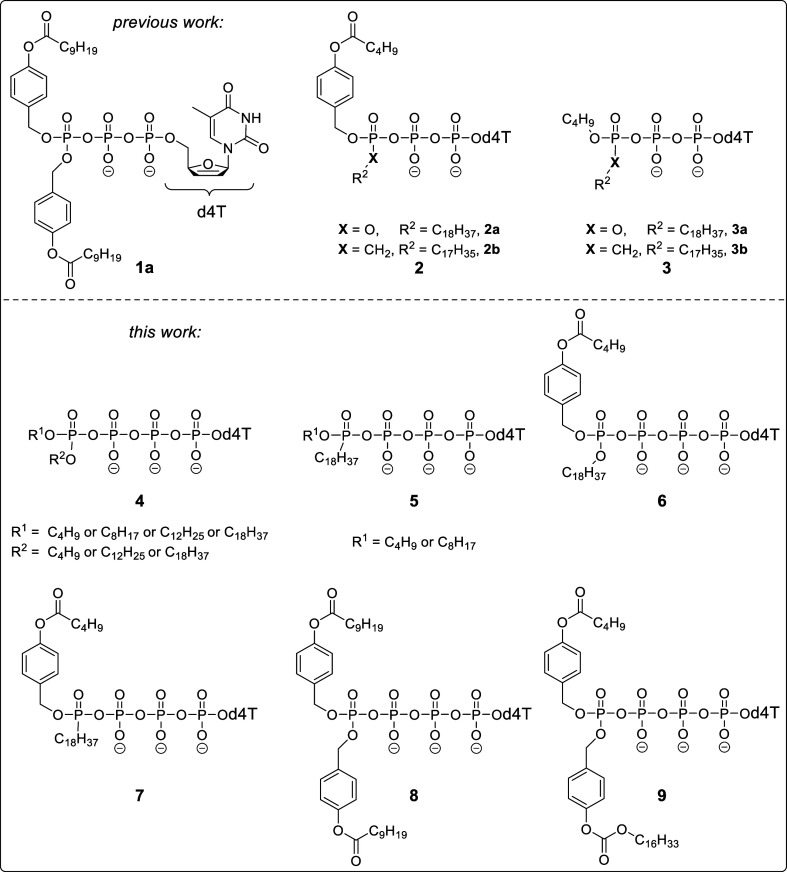
General structures of Tetra*PPPP*ro-nucleotides **4**–**9**.

Taking the results summarized above into account, we report on the synthesis and biological activities of a series of new Tetra*PPPP*ro-nucleotides **4** and **5** [δ-phosph(on)ate-modified-d4TTPs, Figure 1]. It was expected that with these compounds, a selective conversion of the compounds **4** and **5** into nucleoside triphosphates such as d4TTP may be achieved. Furthermore, we also report on the synthesis of two types of nucleoside tetraphosphate prodrugs: (1) δ-(AB-C4; alkyl-C18)-d4T4P **6** and δ-(AB-C4)-δ-C-(alkyl-C18)-d4T4P **7** bearing one biodegradable prodrug moiety in addition to a noncleavable moiety at the δ-phosph(on)ate unit and (2) δ-(AB-C9; AB-C9)-d4T4P **8** and δ-(AB-C4; ACB-C16)-d4T4P **9** bearing two lipophilic biodegradable masking units providing different stabilities attached to the δ-phosphate group.

Moreover, we summarize chemical and enzymatic studies to evaluate their hydrolysis behavior. Finally, we performed primer extension assays and evaluated the antiviral activity in HIV-1- and HIV-2-infected wild-type (CEM/0) cells and in HIV-2-infected CEM/TK^–^ cells.

## 2 Results and Discussion

### 2.1 Part I: Synthesis of d4T Comprising Tetra*PPPP*ro-nucleotides 4–9, Monoalkylated Tetraphosphates 20 and 24, and d4T4P

**Scheme 1:**
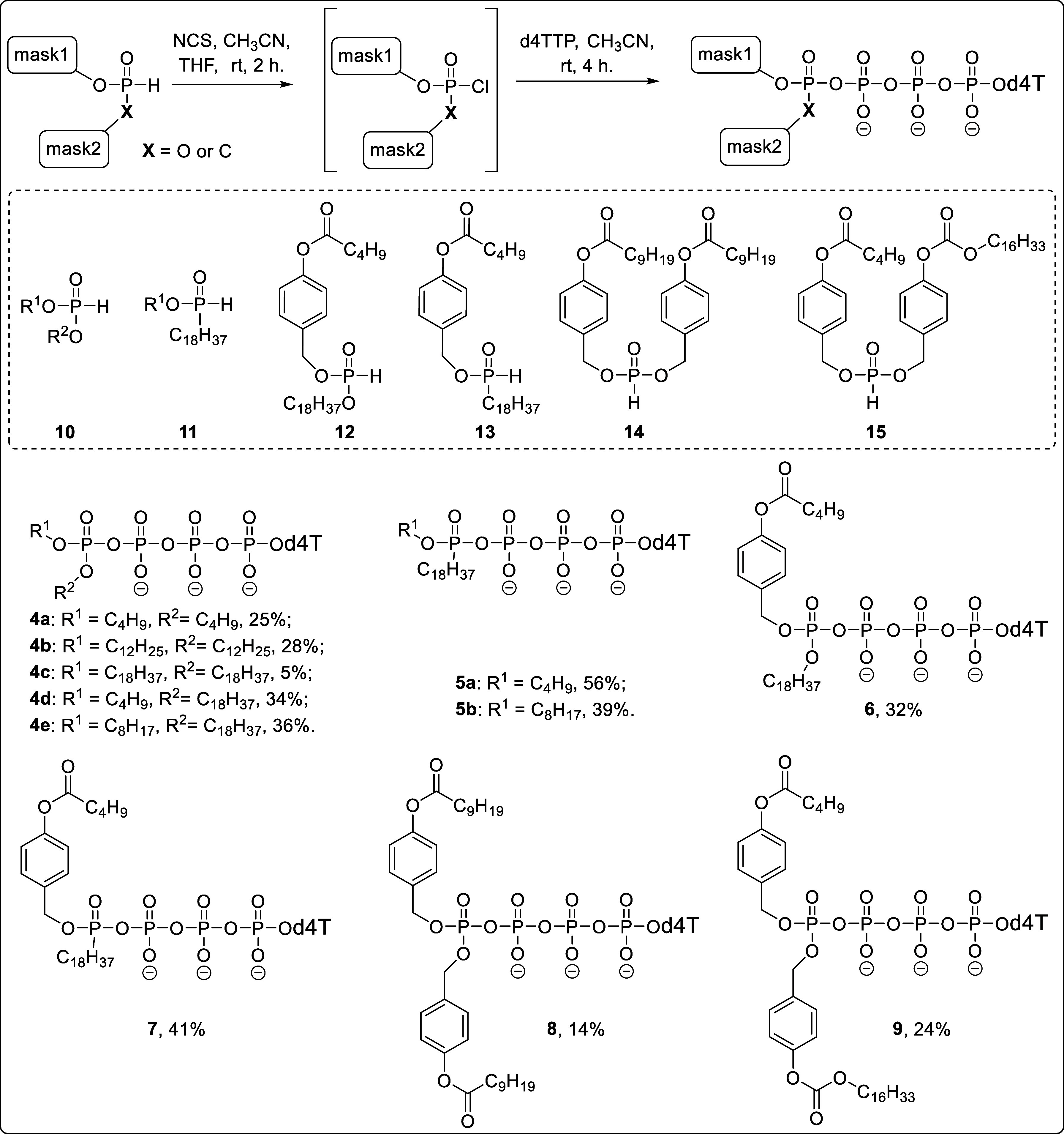
Synthesis of Tetra*PPPP*ro-nucleotides **4**–**9** Using the *H*-Phosphonate and *H*-Phosphinate Routes

**Scheme 2:**
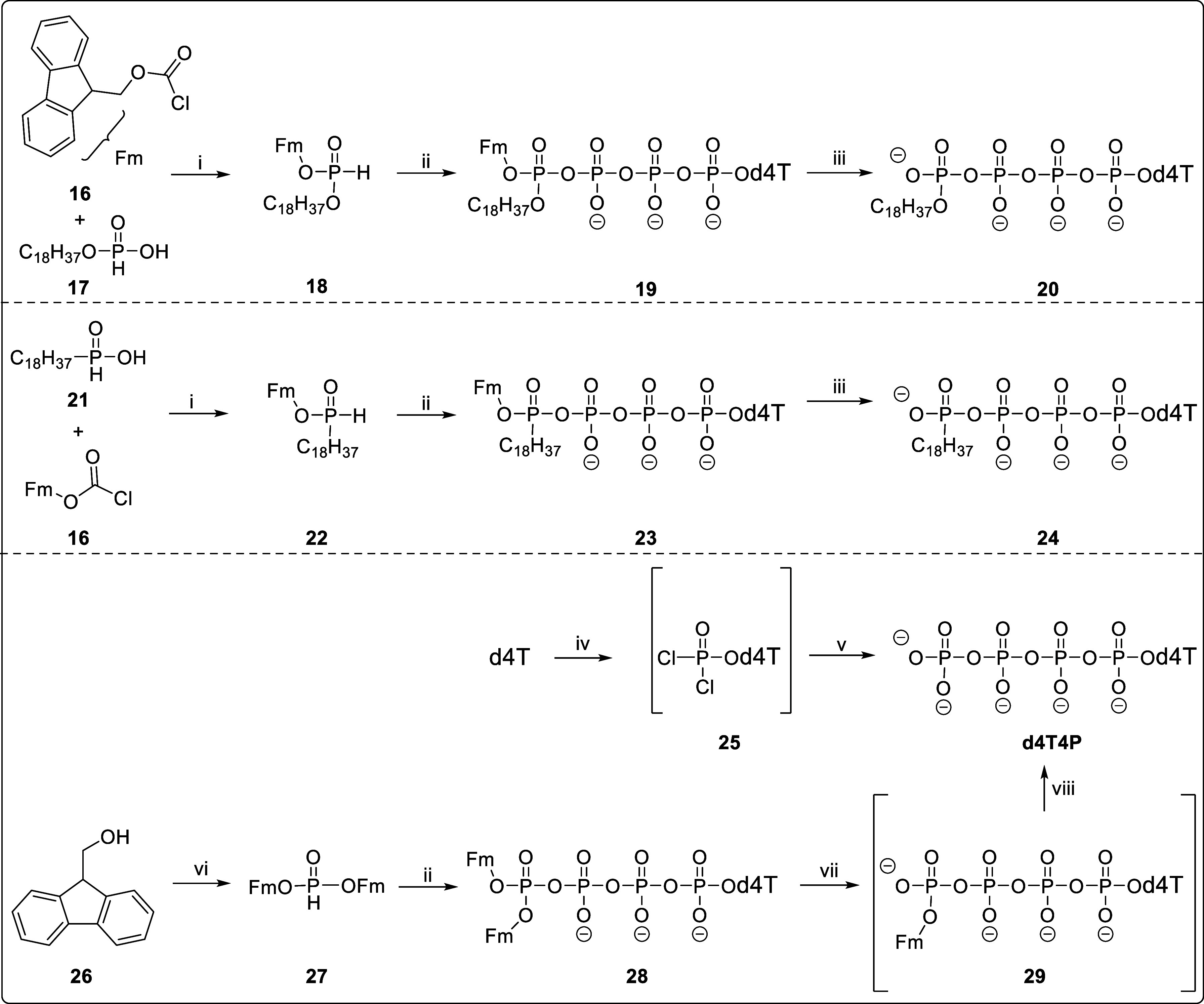
Synthesis of Tetra*PPPP*ro-nucleotides **20** and **24** and d4T4P. Reagents and Conditions: (i) *N*-methylmorpholine, Et_2_O, Toluene, 0 °C-rt, 12 h; (ii) a. CH_3_CN, THF, NCS, rt, 2 h. b. d4TTP (*n*-Bu_4_N^+^ Form), CH_3_CN, rt, 4 h; (iii) Et_3_N, CH_3_CN, 4 h, Dowex 50WX8 (NH_4_^+^ Form) Ion Exchange, 7% (**20**) or 22% (**24**) Overall yield; (iv) POCl_3_, TMP, Proton Sponge, −5 °C, 30 min; (v) (*n*Bu_4_N^+^)_3_(H^+^)_2_(P_3_O_10_)^5–^, Bu_3_N, CH_3_CN, 10 min, 3% Over Steps (iv–v); (vi) DPP, Pyridine, CH_2_Cl_2_, 0 °C-rt, 12 h, 64%; (vii) Et_3_N, CH_3_CN, 10 min; (viii) Et_3_N, CH_3_CN, 24 h, 19% Over Three Steps

For the synthesis of δ-phosphate-modified-d4TTPs **4**, **6**, **8**, and **9** and δ-phosphonate-modified-d4TTPs **5**,**7**, a convergent strategy using *N*-chlorosuccinimide (NCS)-mediated coupling of *H*-phosphonates **10**, **12**, **14**, and **15** or *H*-phosphinates **11** and **13** and d4TTP (*n*-Bu_4_N^+^ form) to form the energetically rich Tetra*PPPP*ro-nucleotides **4**–**9** (Scheme 1) was applied. In a first attempt, a previously reported procedure was applied for the synthesis of compounds **10**–**15**.^44,45,49,50,55^ D4TTP were prepared by using the *cyclo*Sal method.^56^ In the next step, compounds **10**–**15** were reacted with NCS^57^ to afford the corresponding phosphorochloridates and phosphonochloridates. In the last step, Tetra*PPPP*ro-nucleotides **4**–**9** (*n*-Bu_4_N^+^ form) are formed through a nucleophilic attack of d4TTP (*n*-Bu_4_N^+^ form) on the respective phosphorochloridates. After a Dowex 50WX8 (NH_4_^+^) ion exchange column and freeze-drying, Tetra*PPPP*ro-nucleotides **4**–**9** (NH_4_^+^ form) were isolated as colorless solids. Notedly, δ-(AB-C9; AB-C9)-d4T4P **8** was obtained in 85% purity only (NMR spectra and high-performance liquid chromatography (HPLC) chromatogram in Supporting Information), probably due to poor chemical stability. In addition, δ-(Fm; C18)-d4T4P **19** and δ-(Fm)-δ-C-(C18)-d4T4P **23** were successfully synthesized using the same routes as above. Subsequently, the Fm-moiety was cleaved to form the corresponding δ-monoalkylated d4T tetraphosphates in low yields of 7% (**20**) and 22% (**24**) (Scheme 2). It was assumed that the low yield of δ-(C18)-d4T4P **20** (δ-monoalkylated d4T tetraphosphate) correlated with the lability of δ-Fm-protected Tetra*PPPP*ro-compound **19** under basic conditions.

Starting from d4T, the “one-pot-three-step” Ludwig procedure^58^ proved unsuccessful in preparing nucleoside tetraphosphate in satisfying yields. Treatment of d4T with phosphoroxychloride (POCl_3_) in trimethyl phosphate (TMP) and proton sponge, followed by the reaction with tetra-*n*-butylammonium triphosphate using acetonitrile as solvent and tributylamine as base, gave d4T4P in a poor yield of only 3% (Scheme 2, lower section). In order to achieve an efficient chemical synthesis of the nucleoside tetraphosphate, the *H*-phosphonate route was used. For this, first, the (Fm; Fm)-*H*-phosphonate **27** was prepared from 9-fluorenylmethanol **26** and diphenyl hydrogen phosphonate (DPP). Afterward, compound **27** was activated with NCS to give the corresponding phosphorochloridate. Subsequent reaction with d4TTP (*n*-Bu_4_N^+^ form) yielded bis(Fm)-protected compound **28**. This δ-(Fm; Fm)-d4T4P **28** was readily hydrolyzed to form δ-(Fm)-d4T4P **29** with triethylamine (10 min). Intermediate **29** was obtained by reverse-phase column chromatography, followed by a deprotection step (24 h triethylamine treatment) to form d4T4P. In this case, d4T4P was finally obtained in pure form in a yield of 19%.

### 2.2 Part II: Hydrolysis by Chemical or Biological Means

**Scheme 3:**
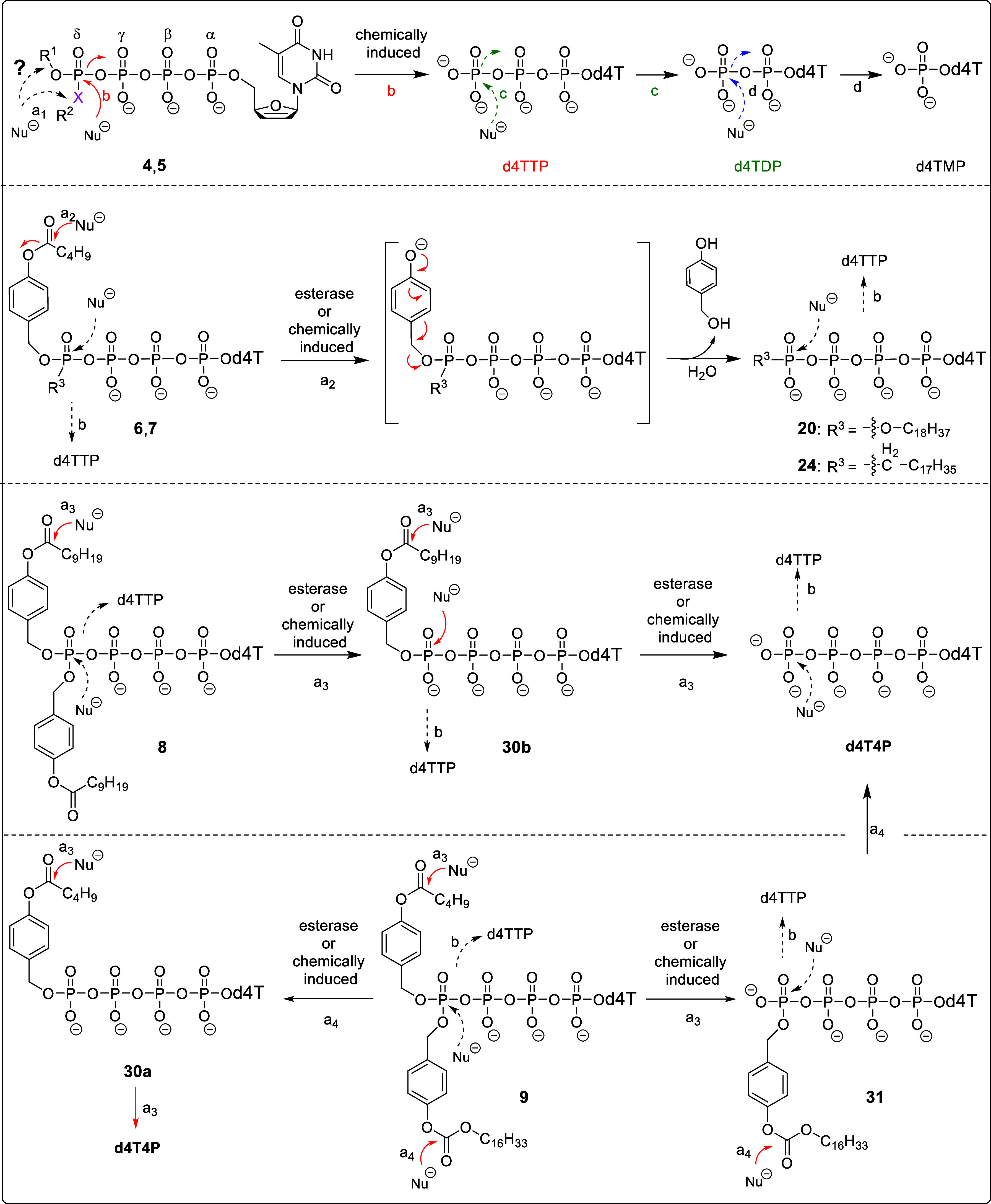
Putative Hydrolysis Pathways of Tetra*PPPP*ro-nucleotides **4**–**9**; the Prodrugs **4**–**9** Target for d4TTP (for Tetra*PPPP*ro-nucleotides **4** and **5**), δ-mono-alkylated d4T Tetraphosphates **20** and **24** (for Tetra*PPPP*ro-nucleotides **6** and **7**), and d4T4P (for Tetra*PPPP*ro-nucleotides **8** and **9**), respectively, by Chemical or Enzymatic Processes

To investigate the hydrolysis properties of Tetra*PPPP*ro-nucleotides **4**–**9** and their capacity to undergo chemical or enzyme-triggered nucleotide release, compounds **4**–**9** were studied in phosphate buffered saline (PBS, 25 mM, pH 7.3 and pH 8.0), citrate buffer (25 mM, pH 2.0), pig liver esterase (PLE), CEM/0 cell extracts, and citrate-stabilized human plasma. All metabolite mixtures were analyzed and quantified by means of analytical RP18-HPLC (UV detection). The half-lives (*t*_1/2_) of Tetra*PPPP*ro-nucleotides **4**–**9** are listed in Table 1, reflecting the initial hydrolysis step of Tetra*PPPP*ro-nucleotides **4**–**9** to yield their corresponding nucleotides and δ-monoprotected compounds **20**, **24**, **30**, and **31**, respectively. The possible chemical and enzymatic hydrolysis pathways of how the masking or biocleavable groups of compounds **4**–**9** may be cleaved are summarized in Scheme 3. Conceptually, we expected for each of the different prodrug compounds the following preferred hydrolysis pathways. Compounds **4** and **5** bearing two nonhydrolyzable moieties attached to the γ-phosph(on)ate d4TTP should be the favored product in all media. The anticipated outcome was that such a combination would undergo slow cleavage, primarily through chemical means, to form d4TTP. In the case of the mixed modified d4T4P derivatives **6** and **7**, the main product in the biological media should be the monoalkylated d4T4P derivatives **20** and **24**. In the chemical hydrolysis, d4TTP might also be formed in parallel. The expectation was that these prodrugs **6** and **7** would bypass all steps of intracellular phosphorylation, thereby maximizing the intracellular concentration of the bioactive monoalkylated nucleoside tetraphosphate analogues **20** and **24**. Finally, for the d4T4Ps **8** and **9** compounds comprising two biocleavable groups, d4TTP should be the favored product. The anticipation was that such a combination would experience more rapid cleavage, particularly through enzymatic means, forming the (AB or ACB)-intermediates **30** and **31**, followed by selective cleavage to form d4T4P.

**Table 1:**
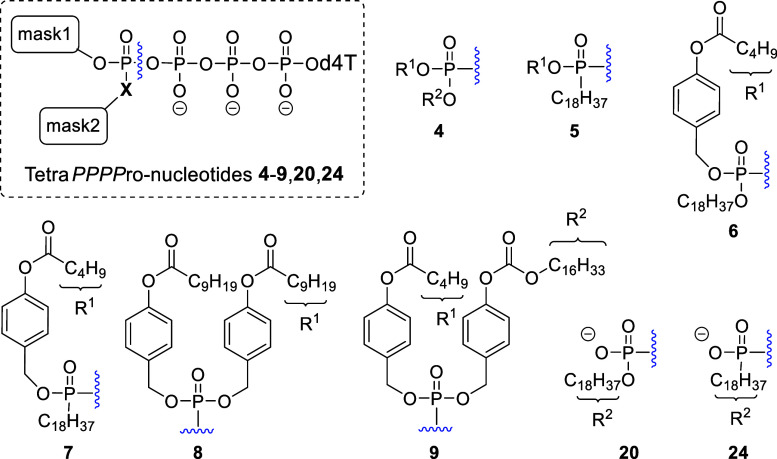
Half-Lives of Tetra*PPPP*ro-nucleotides **4**–**9**, **20**, and **24** and d4T4P in PBS, PLE, and CEM/0 Cell Extracts as Well as Retention Times^a^

			PBS		citrate buffer			
comp	R^1^	R^2^	pH 7.3	pH 8.0	pH = 2.0	CEM/0 cell extracts	PLE	RP-HPLC
			*t*_1/2_ [h]	*t*_1/2_ [h]	*t*_1/2_ [h]	*t*_1/2_ [h]	*t*_1/2_ [h]	*t*_R_ (min)
**4a**	C_4_H_9_	C_4_H_9_	167	n.d.^b^	n.d.^b^	n.d.^b^	n.d.^b^	10.2
**4b**	C_12_H_25_	C_12_H_25_	215	n.d.^b^	n.d.^b^	n.d.^b^	n.d.^b^	14.7
**4c**	C_18_H_37_	C_18_H_37_	n.d.^b^	n.d.^b^	n.d.^b^	n.d.^b^	n.d.^b^	20.7
**4d**	C_4_H_9_	C_18_H_37_	251	>100	66	>20	>50	14.5
**4e**	C_8_H_17_^c^	C_18_H_37_	325	n.d.^b^	n.d.^b^	n.d.^b^	n.d.^b^	15.2
**5a**	C_4_H_9_	C_18_H_37_	>1000	>200	>150	>20	>50	14.4
**5b**	C_8_H_17_^c^	C_18_H_37_	>1000	n.d.^b^	n.d.^b^	n.d.^b^	n.d.^b^	15.1
**6**	C_4_H_9_^d^	C_18_H_37_	77	99	27	6.67	1.06	15.3
**7**	C_4_H_9_^d^	C_18_H_37_	>600	n.d.^b^	n.d.^b^	>8	0.50	15.2
**8**	C_9_H_19_^d^	C_9_H_19_^d^	26	n.d.^b^	n.d.^b^	1.37	0.07	15.2
**9**	C_4_H_9_^d^	C_16_H_33_^e^	25	n.d.^b^	n.d.^b^	2.58	1.03	16.0
**20**		C_18_H_37_	132	n.d.^b^	n.d.^b^	>8	>50	13.3
**24**		C_18_H_37_	>1000	n.d.^b^	n.d.^b^	>30	>50	13.2
d4T4P			534	>150	>150	0.37	n.d.^b^	10.2
**1a**	C_9_H_19_^d^	C_9_H_19_^d^	44^43^	68	n.d.^b^	2.8^43^	0.082^43^	16.3

a The hydrolysis experiments of compounds **4**–**9**, **20**, and **24** and d4T4P were conducted in aqueous 25 mM phosphate buffered saline (PBS, pH = 7.3 and 8.0), citrate buffer (pH = 2.0), PLE and CEM/0 cell extracts. The hydrolysis products were detected by analytical RP18 HPLC. **4a**-**e**: δ-(alkyl/C4–C18; alkyl/C4–C18)-d4T4P compounds; **5a**-**b**: δ-(alkyl/C4–C8)-δ-C-(alkyl-C18)-d4T4P compounds; **6**: δ-(AB-C4; alkyl-C18)-d4T4P; **7**: δ-(AB-C4)-δ-(alkyl-C18)-d4T4P; **8**: δ-(AB-C9; AB-C9)-d4T4P; **9**: δ-(AB-C4; ACB-C16)-d4T4P; **20**: δ-(alkyl-C18)-d4T4P; **24**: δ-C-(alkyl-C18)-d4T4P; d4T4P: d4T tetraphosphate; **1a**: γ-(AB-C9; AB-C9)-d4TTP.

b n.d.: not determined.

c R^1^ = C_8_H_17_ (2-ethylheayl).

d acyloxybenzyl (AB)-masking group.

e alkoxycarbonyloxybenzyl (ACB)-masking group. Assay conditions are summarized in the Experimental Section.

#### 2.2.1 Chemical Stability of Tetra*PPPP*ro-nucleotides 4–9 in Aqueous Buffers

##### 2.2.1.1 δ-Dialkyl-modified Compounds 4,5

In PBS, pH 7.3, the chemical stabilities of δ-phosphate-modified-d4TTP compounds **4** (*t*_1/2_ = 167–325 h) and δ-phosphonate-modified-d4TTP compounds **5** (*t*_1/2_ > 1000 h) bearing two identical or different alkyl residues were found to be higher than δ-(AB-C4; C18)-d4T4P **6** (*t*_1/2_ = 77 h) and δ-(AB-C4)-δ-C-(C18)-d4T4P **7** (*t*_1/2_ > 600 h), respectively, bearing only one noncleavable moiety in combination with a biodegradable acyloxybenzyl moiety and significantly higher than the doubly, bioreversibly modified δ-(AB-C9; AB-C9)-d4T4P **8** (*t*_1/2_ = 26 h) and δ-(AB-C4; ACB-C16)-d4T4P **9** (*t*_1/2_ = 25 h). Generally, all δ-phosphonates comprising derivatives **5** proved to be more stable than their corresponding δ-phosphate counterparts **4**. Interestingly, the half-lives of compounds **4** and **5** were found to be lower than the previously reported γ-dialkyl-modified compound **3** [third generation Tri*PPP*ro-compounds: γ-(alkyl-C4)-γ-C-(alkyl-C18)-d4TTP, *t*_1/2_ > 1700 h]^55^ and β-dialkyl-modified compounds **32** [third generation Di*PP*ro-compounds: β-(alkyl-C4)-β-C-(alkyl-C18)-d4TDP, *t*_1/2_ > 3000 h].^55^

**Figure 2:**
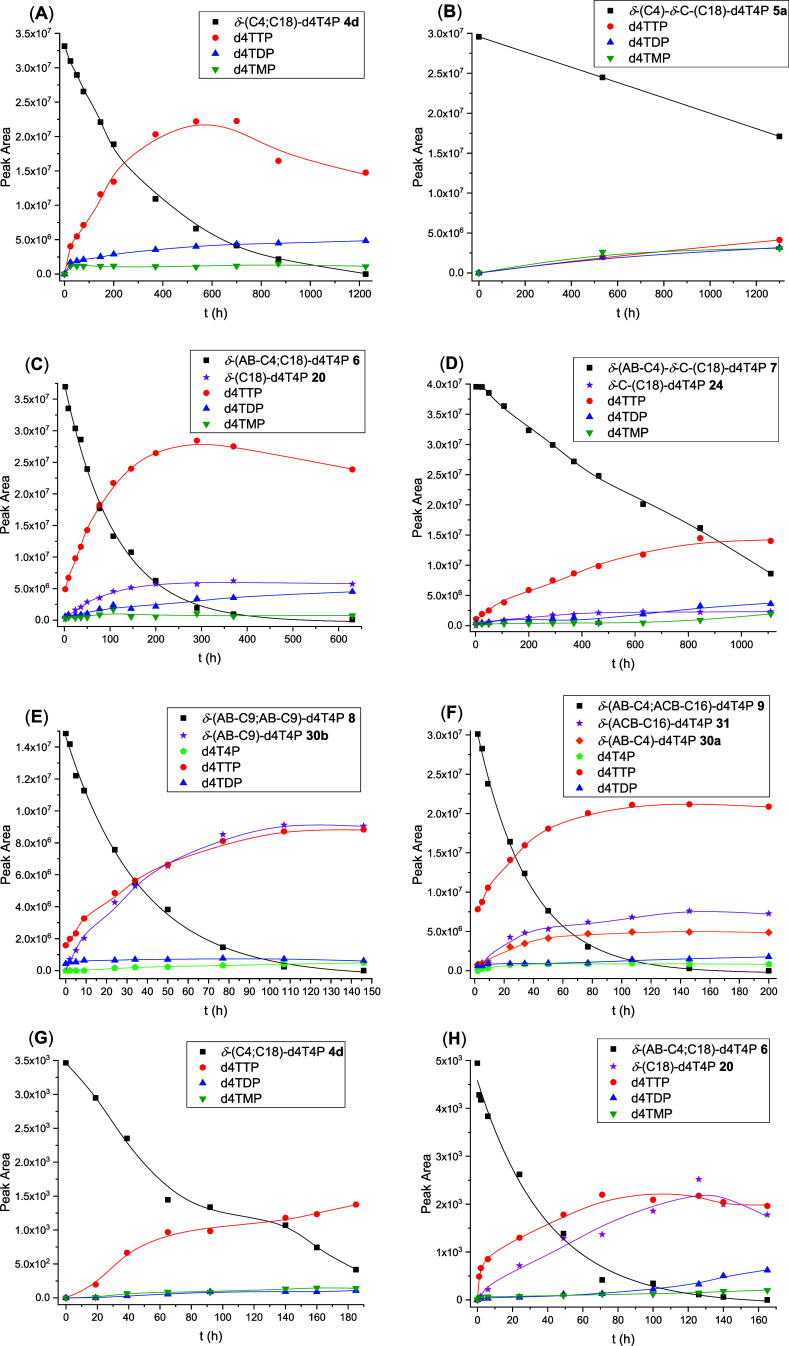
Hydrolysis of Tetra*PPPP*ro-nucleotides **4**–**9** in PBS (25 mM, pH 7.3, Figure (A–F) and compounds **4d** and **6** in citrate buffer (25 mM, pH 2.0, Figure (G–H). (Figure **2A**) δ-(C4; C18)-d4T4P **4d**, (Figure **2B**) δ-(C4)-δ-C-(C18)-d4T4P **5a**, (Figure **2C**) δ-(AB-C4; C18)-d4T4P **6**, (Figure **2D**) δ-(AB-C4)-δ-C-(C18)-d4T4P **7**, (Figure **2E**) δ-(AB-C9; AB-C9)-d4T4P **8**, (Figure **2F**) δ-(AB-C4; ACB-C16)-d4T4P **9**, (Figure **2G**) δ-(C4; C18)-d4T4P **4d**, and (Figure **2H**), δ-(AB-C4; C18)-d4T4P **6**.

In the case of the hydrolysis of δ-(alkyl-C4; alkyl-C18)-d4T4P **4d** (Figures 2A and S1; Supporting Information) and δ-(alkyl-C4)-δ-C-(alkyl-C18)-d4T4P **5a** (Figures 2B and S3; Supporting Information) in PBS (pH 7.3), the formation of d4TTP (pathway b, Scheme 3, upper section) was observed and no formation of δ-monoalkylated compounds **20** and **24** (pathway a_1_, Scheme 3, upper section) was detected, which was fairly consistent with the previously published cleavage pathways for γ-dialkyl-modified nucleoside triphosphate analogues **3**(55) and β-dialkyl-modified nucleoside diphosphate analogues **32**(55) in PBS (pH 7.3), Furthermore, before complete consumption of the starting materials **4** and **5** (Figures 2A,B and S1–S4; Supporting Information), an increase of the concentrations of d4TDP (pathway c, Scheme 3, upper section) and also d4TMP (pathway d, Scheme 3, upper section) was detected, although in really small amounts (<10%). Additionally, we observed that d4TTP was cleaved to form d4TDP (Figure S5; Supporting Information), while d4TDP was hydrolyzed to yield d4TMP very slowly and thus in very small amounts (Figure S6; Supporting Information). At the same time, a cleavage of the glycosidic bond in δ-dialkyl-modified compounds **4** and **5** (Figures S1–S4; Supporting Information) and the nucleotides (Figures S5–S6; Supporting Information) occurred, as proven by the appearance of the nucleobase thymine.

##### 2.2.1.2 δ-[Acyloxybenzyl (AB); alkyl]-Modified Compounds 6,7

In PBS (pH 7.3), the hydrolytic stability of δ-(AB-C4; C18)-d4T4P **6** (*t*_1/2_ = 77 h) was 3- and 5-fold lower than the corresponding γ-(AB-C4; C18)-d4TTP **2a** (*t*_1/2_ = 237 h)^49^ and β-(AB-C4; C18)-d4TDP **33a** (*t*_1/2_ = 406 h),^55^ respectively. The half-live of δ-(AB-C4; C18)-d4T4P **6** was also found to be lower than δ-(AB-C4)-δ-C-(C18)-d4T4P **7** (*t*_1/2_ > 600 h). The result was similarly to that previously reported for the corresponding Tri*PPP*ro-compounds **2a** and **b**(49,50) (second generation Tri*PPP*ro-compounds: γ-(AB-C4; C18)-d4TTP **2a** and γ-(AB-C4)-γ-C-(C18)-d4TTP **2b** (*t*_1/2_ = 646 h)) and Di*PP*ro-compounds **33**(55) [second generation Di*PP*ro-compounds: β-(AB-C4; C18)-d4TDP **33a** and β-(AB-C4)-β-C-(C18)-d4TDP **33b** (*t*_1/2_ > 1000 h)]. Furthermore, the initial cleavage of the AB-moiety in Tetra*PPPP*ro-derivatives **6** and **7** was introduced by ester hydrolysis, and the hydrolysis mechanism proceeded similarly to the published cleavage pathways for Tri*PPP*ro-compounds **2a** and **b**(49,50) and Di*PP*ro-compounds **33a** and **b**(55) (pathway a_2_, Scheme 3). Therefore, an increase of the amount of δ-(C18)-d4T4P **20** (Figures 2C and S7; Supporting Information) and δ-C-(C18)-d4T4P **24** (Figures 2D and S8; Supporting Information) was detected, but in low concentration in these studies. Also, d4TTP and d4TDP were detected when Tetra*PPPP*ro-compounds **6** and **7** were incubated in PBS (pH 7.3) (Figures 2C,D, and S7–S8; Supporting Information), which supports the hypothesis that δ-modified-prodrugs **6** and **7** were susceptible to a bond breakage between the δ-phosph(on)ate and the γ-phosphate or between the γ-phosphate and the β-phosphate. Additionally, no d4T4P was observed because of the noncleavable moieties attached to the δ-phosph(on)ate group.

##### 2.2.1.3 δ-modified Compounds 8 and 9 Bearing Two Biocleavable Groups

As compared to the studies of δ-(AB-C9; AB-C9)-d4T4P **8** [releasing the intermediate, δ-(AB-C9)-d4T4P **30b**, Figures 2E and S9; Supporting Information; pathway a_3_, Scheme 3], the hydrolysis of δ-(AB-C4; ACB-C16)-d4T4P **9** in PBS released two different intermediates, δ-(AB-C4)-d4T4P **30a** and δ-(ACB-C16)-d4T4P **31** (Figures 2F and S10; Supporting Information). In contrast to δ-(AB-C9; AB-C9)-d4T4P **8**, the hydrolysis mechanism of δ-(AB-C4; ACB-C16)-d4T4P **9** in PBS (pH 7.3) involves an ester and a carbonate pathway and is shown in Scheme 3 (lower section). The pathway a_3_ was initiated by an ester cleavage and yielded the monomasked intermediate **31** (major), while pathway a_4_ shows the hydrolysis of δ-(AB-C4; ACB-C16)-d4T4P **9** initiated by a carbonate hydrolysis that led to the formation of intermediate **30a** (minor). Then, both intermediates **30** and **31** released d4T4P (Figures 2E,F and S9–S10; Supporting Information) selectively, with the subsequent cleavage to d4TTP and d4TDP in less than 10% as well (Figure S11; Supporting Information). Consequently, the chemically triggered release of nucleoside tetraphosphate, triphosphate, and diphosphate from Tetra*PPPP*ro-prodrugs **8** and **9** was observed.

##### 2.2.1.4 Hydrolysis in Acidic Aqueous Conditions, pH 2.0 and pH 8.0

Next, Tetra*PPPP*ro-nucleotides **4d**, **5a**, and **6** were further studied in citrate buffer (pH 2.0) and in phosphate buffer (pH 8.0) at 37 °C. As is shown in Table 1 and as expected, the half-lives of these prodrugs at pH 2.0 were found to be lower than the half-lives in PBS (pH 7.3). The main reason is that esters are easier to cleave under acidic conditions. Also, protonation of the phosphate moieties may influence the stability of the anhydride bonds. Nevertheless, the selected compounds exhibited considerable stability even under these conditions. The lowest half-life was detected for compound **6** at 27 h. During the course of the hydrolysis, the formation of the corresponding nucleotides (d4TTP, Figure 2G) and δ-monoalkylated d4T tetraphosphates (**20**, Figure 2H) was detected. Surprisingly, as compared to pH 7.3, at pH 8.0, an even greater hydrolytic stability was determined for some examples of compounds **4**–**9**, e.g., compound **6**. This might be associated with the slightly higher degree of deprotonation of the phosphate moieties. This higher degree results in a more significant electrostatic repulsion of the incoming hydroxide ion, which even results in a slower cleavage of the ester comprising the masking group of compounds **6** (*t*_1/2_; pH 7.3:77 h vs *t*_1/2_; pH 8.0:99 h). This increase was also observed for reference compound **1a**, which comprises two ester-bearing masking groups (*t*_1/2_; pH 7.3:44 h vs *t*_1/2_; pH 8.0:68 h). As a conclusion, Tetra*PPPP*ro-nucleotides **4**–**9** followed a cleavage pathway, as depicted in Scheme 3, demonstrating consistent behavior across various pH conditions.

It is noteworthy that in all cases of chemical hydrolysis in which the starting materials **4**–**9** disappeared, an increase in the d4TTP concentration was observed (Figures 2A–H and Figures S1–S4 and S7–S10; Supporting Information). It was concluded that d4TTP was formed from the starting Tetra*PPPP*ro-compounds **4**–**9** by a nucleophilic attack at the δ-phosphate or δ-phosphonate moiety, respectively (pathway b, Scheme 3). In the case of compounds **8**, **9**, **30**, and **31** also comprising one or two cleavage masks (AB or ACB), the hydrolysis of the masks occurred, yielding d4T4P derivatives (trace, Figure 2E,F) in addition to d4TTP.

#### 2.2.2 Enzymatic Activation of Tetra*PPPP*ro-Nucleotides 4–9

##### 2.2.2.1 Hydrolysis Study Using PLE

**Figure 3:**
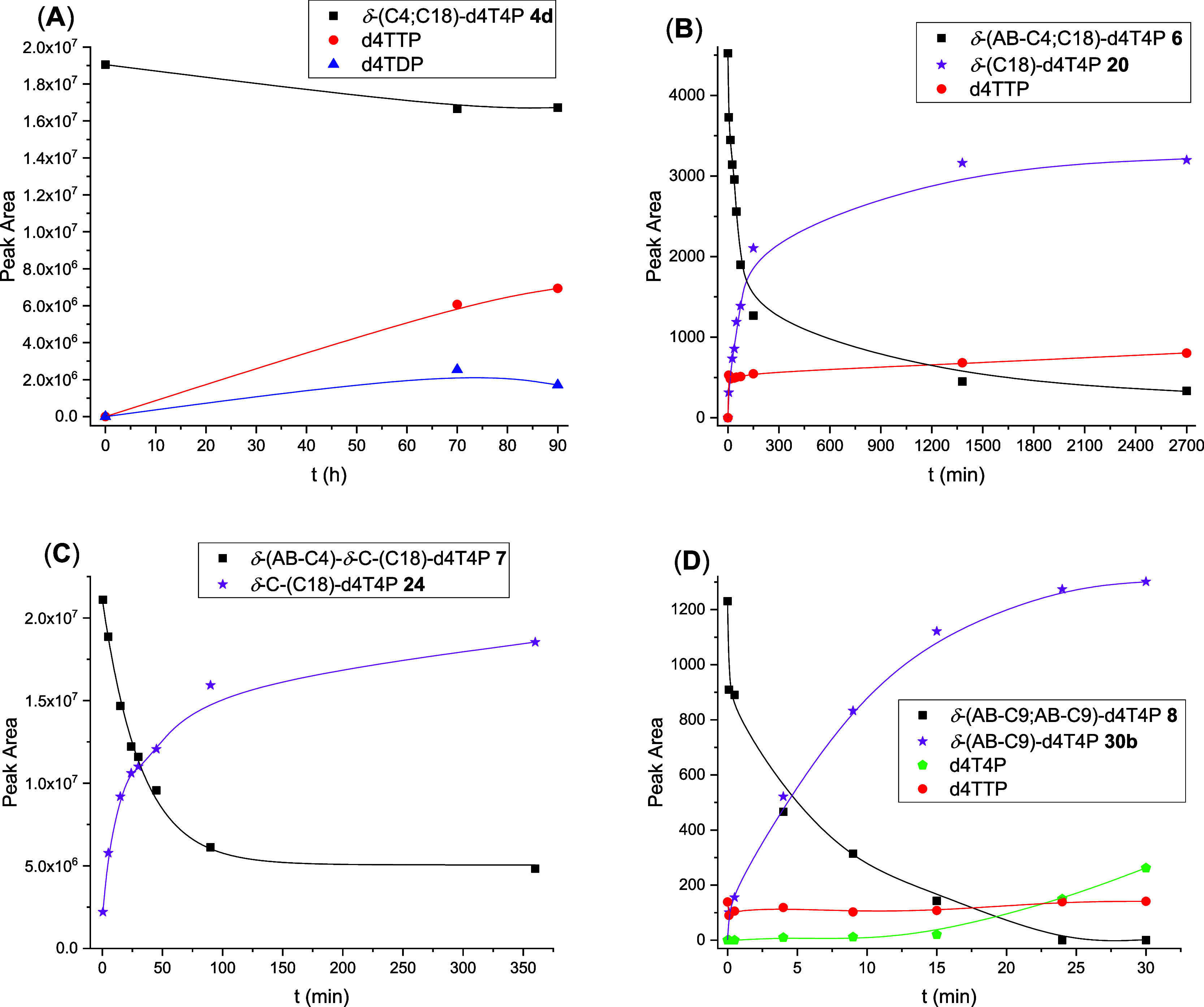
Hydrolysis of Tetra*PPPP*ro-nucleotides **4**–**9** with PLE. (Figure **3A**) δ-(C4; C18)-d4T4P **4d**, (Figure **3B**) δ-(AB-C4; C18)-d4T4P **6**, (Figure **3C**) δ-(AB-C4)-δ-C-(C18)-d4T4P **7**, and (Figure **3D**) δ-(AB-C9; AB-C9)-d4T4P **8**.

To confirm the enzymatic hydrolysis process, Tetra*PPPP*ro-nucleotides **4**–**9** bearing different kinds of lipophilic groups attached to the δ-phosph(on)ate group were incubated with PLE in phosphate buffer. The cleavage of δ-phosph(on)ate-modified-d4TTP compounds **4**,**5** (*t*_1/2_ > 50 h, Table 1) bearing two noncleavage residues led again only to the formation of d4TTP (Figures 3A and S12; Supporting Information) but proceeded much slower than in the cases of Tetra*PPPP*ro-compounds **6**–**9** (*t*_1/2_ = 0.07–1.06 h, Table 1) comprising at least one biodegradable moiety (AB or ACB) (Figures 3B,C,D and S13; Supporting Information). The cleavage of the ester unit within the cleavable masking groups (AB: C4 or C9) in Tetra*PPPP*ro-compounds **6**–**8** was induced by enzymatic means to form δ-alkylated nucleoside tetraphosphate derivatives **20** and **24** (Figure 3B,C) and the δ-monomasked AB-intermediate **30b** (Figures 3D and S13; Supporting Information), respectively. Thus, a large amount of δ-monomasked compounds **20**, **24**, and **30b** and only a small amount of d4TTP were observed, which was different from the results obtained from the studies of compounds **6**–**8** in PBS (Figures 2C,D,E and S7–S9; Supporting Information). In contrast to the studies of δ-(AB-C4; C18)-d4T4P **6** (Figure 3B) and δ-(AB-C4)-δ-C-(C18)-d4T4P **7** (Figure 3C), an increase in the d4T4P concentration was detected during the enzymatic hydrolysis experiment using prodrug δ-(AB-C9; AB-C9)-d4T4P **8** (Figures 3D and S13; Supporting Information). As a consequence, the pure chemical cleavage of alkyl residues in δ-modified-d4T4P compounds **4** and **5** and the enzymatic cleavage of AB or ACB units in Tetra*PPPP*ro-compounds **6**–**9** confirmed our initial concept of introducing different kinds of masking groups attached to δ-phosph(on)ate units, either leading to d4TTP, δ-alkylated d4T4P derivatives **20** and **24**, or d4T4P in dependence of the starting nucleoside tetraphosphate analogues.

##### 2.2.2.2 Hydrolysis in Human CD4^+^ T-Lymphocyte CEM/0 Cell Extracts

Next, Tetra*PPPP*ro-nucleotides **4**–**9** were incubated in human CD4^+^ T-lymphocyte CEM/0 cell extracts to study their stability and identify the hydrolysis products. As expected, the half-lives determined for δ-phosph(on)ate-modified-d4TTP compounds **4** and **5** comprising two *hydrolytically stable* alkyl residues were very high (*t*_1/2_ > 20 h). At the same time, compounds **4** and **5** proved to be stable in CEM/0 cell extracts toward dephosphorylation. The stabilities of compounds **4** and **5** were also found to be markedly higher than δ-(AB; alkyl)-modified compounds **6** and **7** (*t*_1/2_ = 6.7–8 h) and the compounds **8** and **9** bearing two biocleavable moieties (*t*_1/2_ = 1.37–2.58 h).

**Figure 4:**
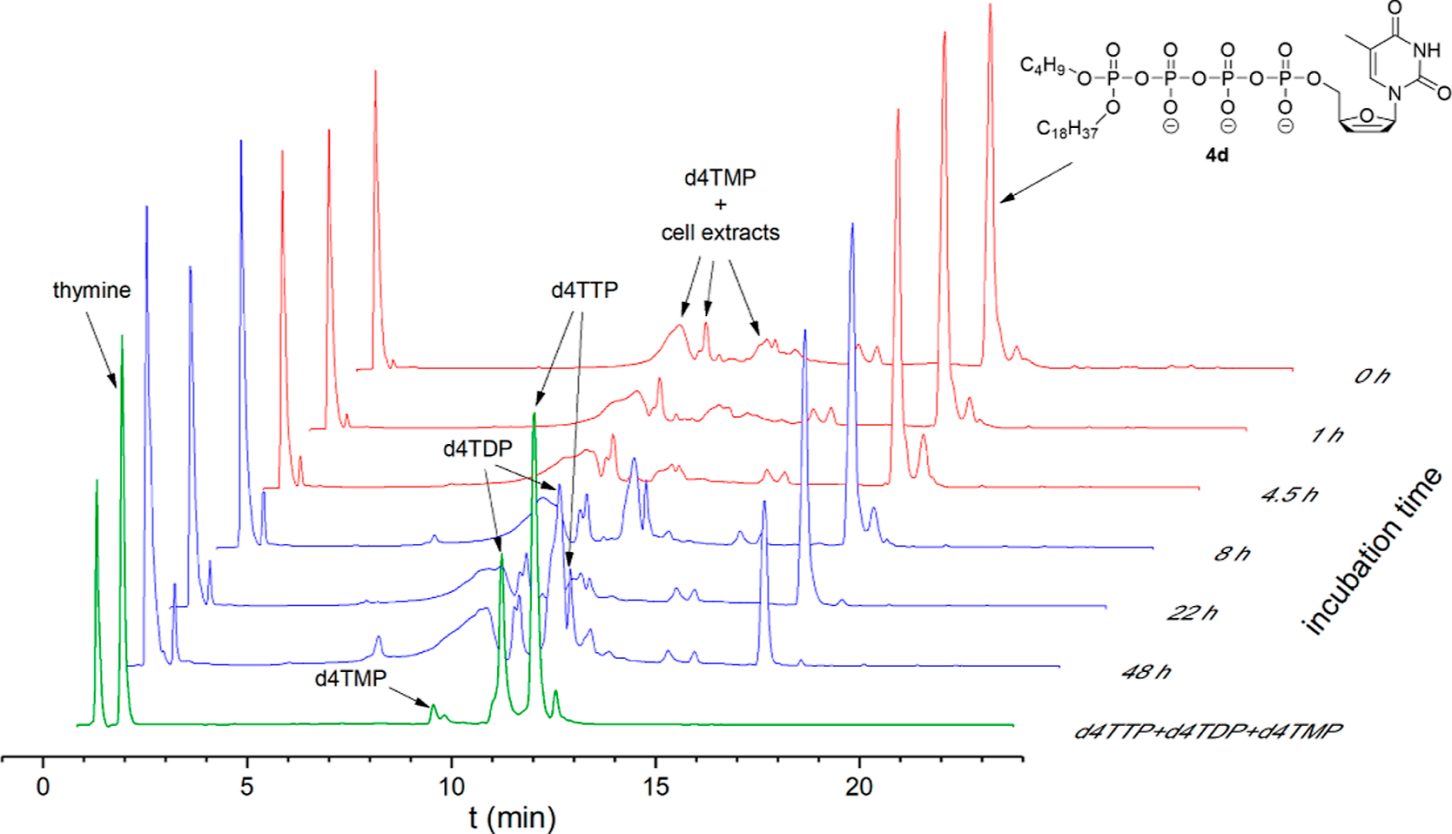
HPLC profiles of compound **4d** after incubation in CEM/0 cell extracts.

As shown in Figures 4 and S14 (Supporting Information), compounds **4** and **5** were slowly hydrolyzed to form a small amount of d4TTP, which was subsequently rapidly dephosphorylated (*t*_1/2_ = 38 min)^43^ to form d4TDP in CEM/0 cell extracts. In contrast, the monomasked compounds **20**, **24**, and **30** were detected in these studies using Tetra*PPPP*ro-prodrugs **6**–**8** (Figures S15–S17; Supporting Information), which was similar to the results obtained from the studies in PBS or with PLE. Additionally, the formation of d4TTP (trace) and d4TDP from δ-(alkyl-C18)-d4T4P **20** (Figure S18; Supporting Information) and δ-C-(alkyl-C18)-d4T4P **24** (Figure S19; Supporting Information) was detected. However, for enzyme-cleavable Tetra*PPPP*ro-compound **8**, it was not possible to calculate the exact peak area for d4T4P (*t*_1/2_ = 22 min) and d4TTP (*t*_1/2_ = 38 min)^43^ probably due to their instability in CEM/0 cell extracts and the collecting peaks resulting from the CEM/0 cell extracts and themselves (Figure S17; Supporting Information). The dephosphorylation of d4T4P and d4TTP is most probably catalyzed by cellular phosphatases and leads to a stepwise degradation of the polyphosphates to the lower phosphorylated metabolites.

##### 2.2.2.3 Hydrolysis in Human Plasma

**Figure 5:**
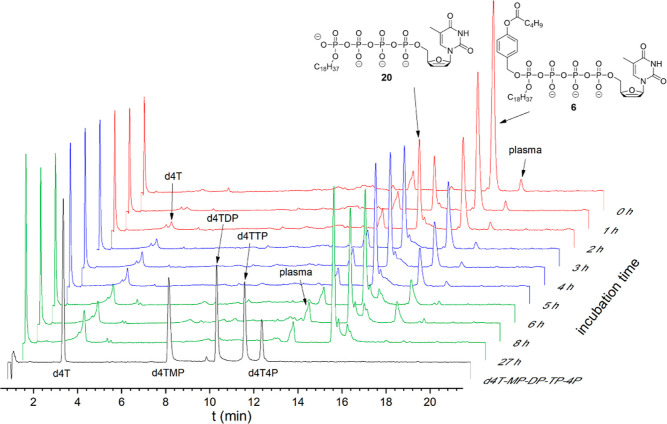
HPLC profiles of compound **6** after incubation in citrate-stabilized human plasma.

Further, selection of three Tetra*PPPP*ro-nucleotides **5a**, **6**, and **8** was incubated in citrate-stabilized human plasma at 37 °C as well. As compared to the double-biocleavably modified δ-(AB-C9; AB-C9)-d4T4P **8** (*t*_1/2_ = 2.8 h) and the alkylated and cleavably modified δ-(AB-C4; alkyl-C18)-d4T4P **6** (*t*_1/2_ = 2.1 h), the half-live for δ-bis-alkyl-phosphonate modified nucleoside analogue **5a** (*t*_1/2_ > 22 h) was found to be higher by at least 8-fold. As is shown in Figure S20 (Supporting Information), almost no peaks corresponding to d4TTP, d4TDP, or d4TMP and δ-monoalkylated compounds **20** and **24** (e.g., **24**; *t*_1/2_ > 22 h; Figure S21, Supporting Information) were formed from compound **5a**. In contrast, the starting materials **6** and **8** disappeared, and the expected δ-(alkyl-C18)-d4T4P **20** and δ-(AB-C9)-d4T4P **30b** were formed, respectively, as shown in Figures 5 and S22 (Supporting Information). Moreover, after complete consumption of the initial Tetra*PPPP*ro-compounds **6** and **8**, d4T was detected in some amounts.

##### 2.2.2.4 Lipophilicity for Tetra*PPPP*ro-nucleotides 4–9

All compounds **4**–**9** were analyzed by RP18-HPLC, and their retention times using an identical elution gradient were used to estimate qualitatively their relative lipophilicity (Table 1). The retention times of most of Tetra*PPPP*ro-nucleotides **4**–**9** were in the range of 14.4 to 16.0 min. As can be seen in Table 1, the lipophilicities of compounds **4a**-**c** (R^1^ = R^2^: C4–C18), **4d**-**e** (R^1^: C4–C8; C18), and **5a**-**b** (R^1^: C4–C8; C18) increased as expected with increasing alkyl chain lengths. The lipophilicity of δ-phosphate-modified-d4TTP compounds **4d** and **e** (*t*_*R*_ = 14.5–15.2 min) was in the same range as δ-phosphonate-modified-d4TTP compounds **5a** and **b** (*t*_*R*_ = 14.4–15.1 min). Thus, the phosphate or phosphonate moiety has no influence on lipophilicity. Moreover, there is almost no difference between the δ-alkylated nucleoside tetraphosphate derivatives **20** (*t*_*R*_ = 13.3 min) and **24** (*t*_*R*_ = 13.2 min).

In comparison, the retention times of Tetra*PPPP*ro-nucleotides **4**–**9** were found to be lower than the previously studied Tri*PPP*ro-compounds **1**–**3** and Di*PP*ro-compounds **32** and **33** when applying the same gradient to the HPLC system.^55^ As an example, the retention time of δ-(alkyl-C4; alkyl-C18)-d4T4P **4d** (*t*_*R*_ = 14.5 min) was lower than γ-(alkyl-C4:alkyl-C18)-d4TTP **3** (*t*_*R*_ = 16.2 min)^55^ and β-(alkyl-C4:alkyl-C18)-d4TTP **32** (*t*_*R*_ = 18.9 min).^55^ This is, of course, due to the different numbers of negative charges that remain at the polyphosphate moiety. The higher polarity of the tetraphosphate compounds disclosed here might also be a reason for the lower stability under the chemical hydrolysis conditions described above.

### 2.3 Part III. Primer Extension Assays

Encouraged by the previously obtained results that γ-phosphate-modified-d4TDPs **3**, γ-monoalkylated d4TTP derivatives as well as d4TDP were accepted as substrates by HIV-RT,^49,50,55,59−61^ δ-dialkylated d4T tetraphosphate analogues **4**,**5**, δ-monoalkylated d4T tetraphosphate analogues **20** and **24** and d4T4P were investigated in primer extension assays in the presence of HIV-RT or the three different human DNA polymerases α, β and γ. D4TTP (for the compounds **4**–**9**) and the natural thymidine triphosphate (TTP) were used as reference compounds. It was checked prior to the primer extension assay that compounds **4a**, **4b**, **5a**, **20**, and **24** proved to be stable under the assay conditions.

**Figure 6:**
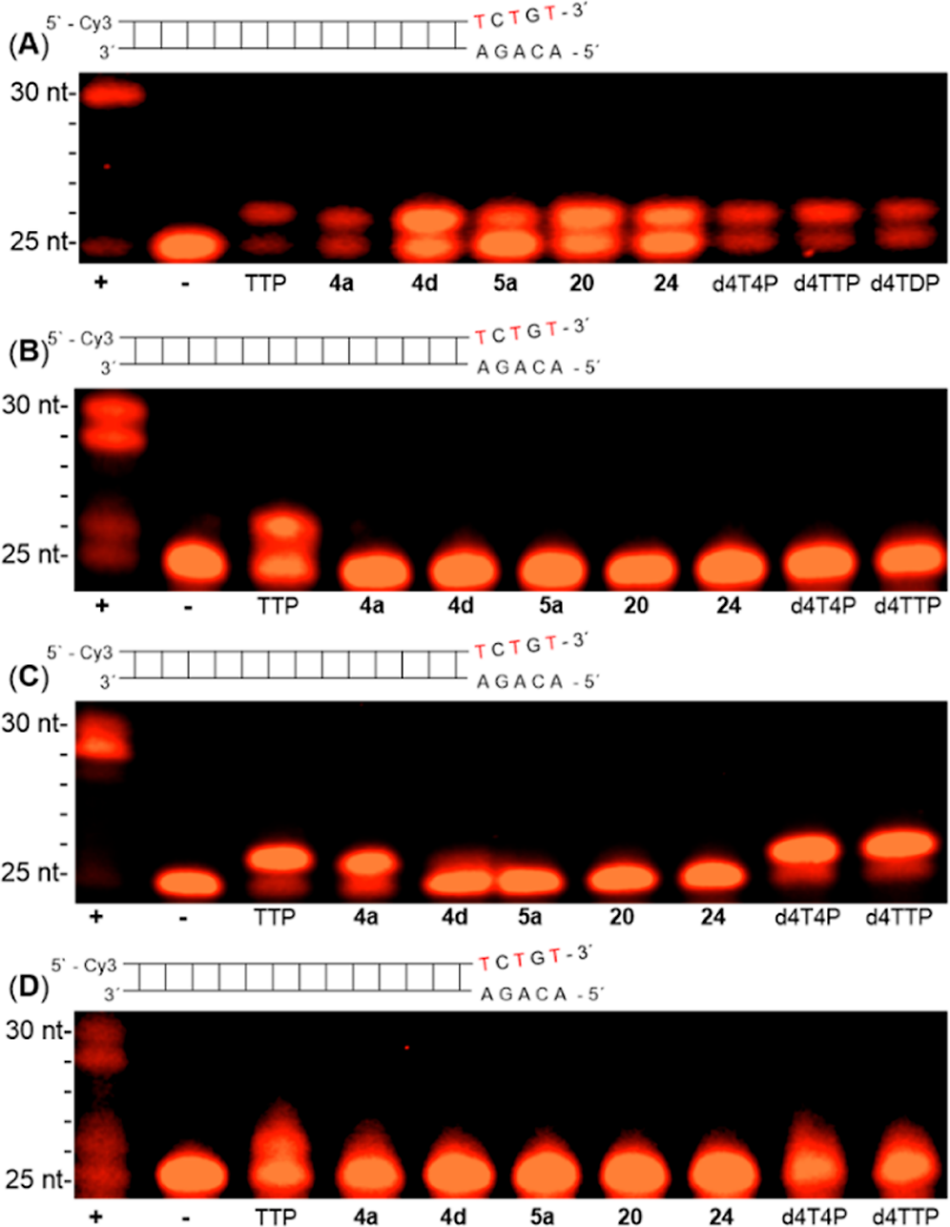
Primer extension assay using HIV-RT (**A**), human polymerases α (**B**), β (**C**), and γ (**D**). (Figure **6A**). Primer extension assay using HIV-RT (30 min, 6U). Lane 1: (+) dATP, dCTP, dGTP, and TTP with HIV-RT. Lane 2: (−) dATP, dCTP, dGTP, and TTP without HIV-RT. Lane 3: TTP. Lane 4: δ-(C4; C4)-d4T4P **4a**. Lane 5: δ-(C4; C18)-d4T4P **4d**. Lane 6: δ-(C4)-δ-C-(C18)-d4T4P **5a**. Lane 7: δ-(C18)-d4T4P **20**. Lane 8: δ-C-(C18)-d4T4P **24**. Lane 9: d4T4P. Lane 10: d4TTP. Lane 11: d4TDP. HIV-RT assay reaction conditions: 50 mM Tris–HCl (pH 8.6), 10 mM MgCl_2_, 40 mM KCl, 250 μM dNTPs, HIV-RT (6 U), 0.20 μM DNA hybrid, primer-template-hybrid 0.32 mM in a reaction volume of 10 μL, incubated at 37 °C for 0.5 h, 80 °C for 7 min. Primer extension assays: 50 mA, 45 w for 4 h. (Figure **6B**). For human DNA polymerase α assay (60 min, 2 U): Lane 1: (+) dATP, dCTP, dGTP, and TTP with human polymerase α. Lane 2: (−) dATP, dCTP, dGTP, and TTP without human polymerase α. Lane 3: TTP. Lane 4: δ-(C4; C4)-d4T4P **4a**. Lane 5: δ-(C4; C18)-d4T4P **4d**. Lane 6: δ-(C4)-δ-C-(C18)-d4T4P **5a**. Lane 7: δ-(C18)-d4T4P **20**. Lane 8: δ-C-(C18)-d4T4P **24**. Lane 9: d4T4P. Lane 10: d4TTP. Human DNA polymerase α assay conditions: 60 mM Tris–HCl (pH 8), 5 mM Mg(OAc)_2_, 100 mM KCl, 1.0 mM dithiothreitol, 0.1 mM spermine, 0.01% (w/v) bovine serum albumin, 250 μM dNTPs, 2 U human polymerase α, 0.20 μM DNA hybrid, and primer-template-hybrid 0.32 mM in a reaction volume of 10 μL, incubated at 37 °C for 1 h, 80 °C for 7 min. Primer extension assays: 50 mA, 45 w for 3 h. (Figure **6C**). For human DNA polymerase β assay (60 min, 2 U): Lane 1: (+) dATP, dCTP, dGTP, and TTP with human polymerase β. Lane 2: (−) dATP, dCTP, dGTP, and TTP without human polymerase β. Lane 3: TTP. Lane 4: δ-(C4; C4)-d4T4P **4a**. Lane 5: δ-(C4; C18)-d4T4P **4d**. Lane 6: δ-(C4)-δ-C-(C18)-d4T4P **5a**. Lane 7: δ-(C18)-d4T4P **20**. Lane 8: δ-C-(C18)-d4T4P **24**. Lane 9: d4T4P. Lane 10: d4TTP. Human DNA polymerase β assay conditions: 50 mM Tris–HCl (pH 8.7), 10 mM Mg(OAc)_2_, 100 mM KCl, 1.0 mM dithiothreitol, 0.1 mM spermine, 0.01% (w/v) bovine serum albumin, 15% glycerol, 250 μM dNTPs, 2 U human DNA polymerase β, 0.20 μM DNA hybrid, and primer-template-hybrid 0.32 mM in a reaction volume of 10 μL, incubated at 37 °C for 1 h, 80 °C for 7 min. Primer extension assays: 50 mA, 45 w for 3 h. (Figure **6D**), For the human DNA polymerase γ assay (120 min, 2 U): Lane 1: (+) dATP, dCTP, dGTP, and TTP with human polymerase γ. Lane 2: (−) dATP, dCTP, dGTP, and TTP without human polymerase γ. Lane 3: TTP. Lane 4: δ-(C4; C4)-d4T4P **4a**. Lane 5: δ-(C4; C18)-d4T4P **4d**. Lane 6: δ-(C4)-δ-C-(C18)-d4T4P **5a**. Lane 7: δ-(C18)-d4T4P **20**. Lane 8: δ-C-(C18)-d4T4P **24**. Lane 9: d4T4P. Lane 10: d4TTP. Human polymerase γ assay conditions: 60 mM Tris–HCl (pH 8), 5 mM Mg(OAc)_2_, 1.0 mM dithiothreitol, 0.1 mM spermine, 0.01% (w/v) bovine serum albumin, 250 μM dNTPs, 2 U human polymerase γ, 0.5 μM MnCl_2_, 0.20 μM DNA hybrid, primer-template-hybrid 0.32 mM in a reaction volume of 10 μL, incubated at 37 °C for 2 h, 80 °C for 7 min. Primer extension assays: 50 mA, 45 w for 3 h.

As expected, with d4TTP, the incorporation of d4TMP was detected, which led to the presence of the expected n+1 (26 nt) band (Figure 6A, lane 10).^49,50,55,59−61^ Here, we could prove that δ-dialkylated compounds **4** and **5**, δ-monoalkylated nucleoside tetraphosphate derivatives **20** and **24**, and d4T4P were all substrates for HIV-RT (Figure 6A). In contrast, they all proved to be nonsubstrates for the cellular DNA polymerases α (Figure 6B) and γ (Figure 6D). Surprisingly, δ-(alkyl-C4; alkyl-C4)-d4T4P **4a** (Figure 6C, lane 4) was also a substrate for DNA polymerase β, while δ-(alkyl-C4; alkyl-C18)-d4T4P **4d** (Figure 6C, lane 5) bearing the longer alkyl moiety was not accepted. No incorporation of d4TMP from δ-(alkyl-C4)-δ-C-(alkyl-C18)-d4T4P **5a** was observed (Figure 6C, lane 6). Additionally, it was interesting to observe that d4T4P was efficiently incorporated into the primer by DNA polymerase β, resulting in an immediate chain termination (Figure 6C, lane 9). The same was detected for d4TTP (positive control; Figure 6C, lane 10).

### 2.4 Part IV. Antiviral Evaluation

Having all prodrugs **4**–**9** in hand, their antiviral activity was evaluated in HIV-1- and HIV-2-infected wild-type (CEM/0) cells and in an HIV-2-infected mutant thymidine kinase-deficient (CEM/TK^–^) cell model. For comparison, a first-generation Tri*PPP*ro-prodrug **1a** bearing two C9 chains attached to the AB group was also evaluated in the same assay. Moreover, we used compounds **2** (AB-alkyl-d4TTP) and **3** (dialkyl-d4TTP) for comparison as well. The resulting data expressed as antiviral activity (EC_50_), cytotoxicity (CC_50_), and selectivity index (SI) are summarized in Table 2. In these assays, some of the Tetra*PPPP*ro-nucleotides **4**–**9** bearing different kinds of masking groups were highly antivirally active against HIV-1 and HIV-2 in wild-type (CEM/0) cells, while others were as active or somewhat less active as their parent nucleoside analogue d4T. Interestingly, some prodrugs **4**–**9** exhibited significant *anti*-HIV-2 activity in HIV-2-infected CEM/TK^–^ cells as well. The parent nucleoside d4T (EC_50_ > 50 μM/HIV-2) and the tetraphosphate d4T4P (EC_50_ = 33.8 μM/HIV-2) displayed very poor *anti*-HIV activity in CEM/TK^–^ cell cultures. The reason for the failure of d4T4P is most probably that the compound is too polar to be uptaken and thus is dephosphorylated to d4T in the extracellular medium during the antiviral assay due to enzymes present in the used fetal calf serum. In contrast, the nucleoside analog d4T gets into the cell and shows activity in the wild-type CEM cells because it is phosphorylated by the kinases to d4TTP.

**Table 2:** Antiviral Activity and Cytotoxicity of Tetra*PPPP*ro-nucleotides **4**–**9**,**20**,**24**, Tri*PPP*ro-d4TTP **1a**, and d4T4P in Comparison with the Parent Nucleoside Analogue d4T

comp	HIV-1 (HE)	HIV-2 (ROD)	CEM/TK^–^	toxicity	selectivity index (SI^c^)	toxicity	selectivity index (SI^e^)
			HIV-2 (ROD)				
	EC_50_^a^ [μM]	EC_50_^a^ [μM]	EC_50_^a^ [μM]	CC_50_^b^ [μM]		CC_50_^d^ [μM]	
**4a**	0.22 ± 0.13	0.16 ± 0.04	14.81 ± 2.8	>100	>625	>100	>6
**4b**	0.17 ± 0.10	0.11 ± 0.05	0.029 ± 0.037	44.6	405	84.9	2927
**4c**	1.99 ± 0.90	2.09 ± 0.58	6.18 ± 1.0	50.0	24	84.1	13
**4d**	0.11 ± 0.01	0.084 ± 0.018	0.18 ± 0.15	43.9	522	46.8	260
**4e**	0.44 ± 0.17	0.32 ± 0.09	0.43 ± 0.21	44.7	139	48.9	114
**5a**	0.33 ± 0.09	0.33 ± 0.12	0.043 ± 0.026	44.6	135	49.8	1158
**5b**	1.44 ± 0.70	1.30 ± 0.64	0.16 ± 0.13	>100	>77	50.0	312
**6**	0.083 ± 0.018	0.063 ± 0.032	0.059 ± 0.054	40.5	643	48.0	813
**7**	0.0092 ± 0.0082	0.0091 ± 0.0077	0.0065 ± 0.0057	39.4	4330	36.9	5677
**8**	0.39 ± 0.16	0.26 ± 0.14	4.44 ± 1.54	45.0	173	50.0	11
**9**	0.17 ± 0.05	0.21 ± 0.11	9.71 ± 4.78	76.8	365	50.0	5
**20**	0.71 ± 0.26	0.50 ± 0.16	4.60 ± 0.33	>100	>200	>100	>21
**24**	0.43 ± 0.37	0.24 ± 0.07	1.35 ± 0.63	23.2	105	40.1	30
d4T4P	0.42 ± 0.16	0.22 ± 0.092	33.80 ± 21.3	>100	454	>100	>3
**1a**	0.042 ± 0.020	0.066 ± 0.026	0.29 ± 0.19	73.2	1109	50.0	172
**2a**(49)	0.18 ± 0.09	0.16 ± 0.10	0.17 ± 0.00	13	76		
**2b**(50)	0.0074 ± 0.0014	0.021 ± 0.006	0.042 ± 0.002	19	452		
**3a**(55)	0.07 ± 0.039	0.041 ± 0.043	0.032 ± 0.031	30	938		
**3b**(55)	0.031 ± 0.017	0.035 ± 0.021	0.018 ± 0.006	33	1830		
d4T	0.33 ± 0.13	0.97 ± 0.50	>50	>50	1	>50	1

a Antiviral activity determined in CD4^+^ T-lymphocytes: 50% effective concentration; values are the mean ± SD of *n* = 2–3 independent experiments.

b Cytotoxicity: 50% cytostatic concentration or compound concentration required to inhibit CD4^+^ T-cell (CEM) proliferation by 50%.

c Selectivity index (SI) was calculated as the ratio of the CC_50_ [μM] and the EC_50_ [μM], HIV-2 (ROD) in CEM cells.

d Cytotoxicity: 50% cytostatic concentration or compound concentration required to inhibit CD4^+^ T-cell (CEM/TK^–^) proliferation by 50%; values are the mean ± SD of *n* = 2–3 independent experiments.

e Selectivity index (SI) was calculated as the ratio of the CC_50_ [μM] and the EC_50_ [μM], HIV-2 (ROD) in CEM/TK^–^ cells.

As can be seen, δ-dialkylated compounds **4a**,**c** showed similar or lower activities against HIV-1 and HIV-2 than the corresponding symmetric δ-dialkylated (C12) compound **4b** in wild-type (CEM/0) CD4^+^ T-cells. It was observed that mixed δ-dialkylated compounds **4d** and **5a** showed similar activities against HIV-1 and HIV-2 compared to compound **4b**. As above, δ-dialkylated compounds **4e** and **5b** showed lower antiviral activity than δ-dialkylated compounds **4d** and **5a**, respectively, probably due to the lower solubility caused by the longer alkyl chain (R^1^). Moreover, δ-dialkylated compounds **4b**, **4d**, and **5a** were as active as or even more active than the parent d4T (EC_50_ = 0.33 μM/HIV-1; EC_50_ = 0.97 μM/HIV-2) against HIV-1 (up to 2 fold) and HIV-2 (3–11 fold) in wild-type (CEM/0) cells. These results also strongly point to the successful cellular uptake of these δ-dialkylated compounds **4** and **5** although these compounds are still highly charged, and point also to an intracellular delivery of nucleotide metabolites.

Interestingly, very good *anti*-HIV activity of δ-dialkylated compounds **4b** (EC_50_ = 0.029 μM/HIV-2), **4d** (EC_50_ = 0.18 μM/HIV-2), and **5a** (EC_50_ = 0.043 μM/HIV-2) was detected in HIV-2-infected CEM/TK^–^ cells with >1700-fold, >270-fold, and >1100-fold, respectively, improved as compared to the parent nucleoside d4T (EC_50_ > 50 μM/HIV-2). These results point to a successful uptake of the compounds into the CEM/TK^–^ cells and a delivery of at least phosphorylated metabolites, thus bypassing the missing enzyme thymidine kinase. The improvement of antiretroviral activity may be explained by δ-dialkylated compounds **4d** and **5a** and their hydrolysis products (d4TTP and d4TDP), which are good substrates for HIV-RT in primer extension assays (Figure 6A). Surprisingly, compounds **4a** and **4c** did not show the retention of antiviral activity to the same extent as the other above-mentioned compounds **4b**, **4d**, and **5a**, for some unknown reasons.

A surprising result was that the marked antiviral activity determined for δ-(AB-C4; C18)-d4T4P **6** [EC_50_ = 0.083 μM/HIV-1 and EC_50_ = 0.063 μM/HIV-2 (wild-type CEM/0 cells); EC_50_ = 0.059 μM/HIV-2 (CEM/TK^–^ cells)] and particular to the δ-(AB-C4)-δ-C-(C18)-d4T4P **7** [EC_50_ = 0.0092 μM/HIV-1 and EC_50_ = 0.0091 μM/HIV-2 (wild-type CEM/0 cells); EC_50_ = 0.0065 μM/HIV-2 (CEM/TK^–^ cells)] bearing one noncleavable moiety (alkyl-C18) and a biocleavable group (AB-C4). The most active prodrug of all the described Tetra*PPPP*ro-nucleotides **4**–**9** (Table 2) was δ-(AB-C4)-δ-C-(C18)-d4T4P **7** (*t*_*R*_ = 15.2 min), which is a 36-fold, 107-fold, and 7700-fold increase in antiviral potency as compared to d4T in CEM/0 and CEM/TK^–^ cells, respectively, resulting in a SI value of 5677. The high antiviral activity of the lipophilic δ-(AB-C4)-δ-C-(C18)-d4T4P **7** was probably attributed to the release of δ-C-(C18)-d4T4P **24**, d4TTP, and d4TDP in CEM/0 cell extracts (Figure S16; Supporting Information), all of which acted as substrates for HIV-RT (Figure 6A). Additionally, δ-monoalkylated tetraphosphate **24** proved to be stable in CEM/0 cell extracts (*t*_1/2_ > 30 h). Interestingly, Tetra*PPPP*ro-prodrugs **6** and **7** comprising a noncleavable moiety in addition to an enzymatically cleavable prodrug moiety at the δ-phosph(on)ate group proved to be more active as compared to the δ-(bis-alkyl)phosph(on)ate modified compounds **4** and **5** in all cell cultures. One reason might be the sufficient lipophilicity of the Tetra*PPPP*ro-compounds **6** and **7** combined with a relatively rapid cleavage of the acyloxybenzyl AB-moiety, which led to the formation of δ-monoalkylated nucleoside tetraphosphate derivatives **20** and **24**, which were also slowly cleaved to form the triphosphate and then released the diphosphate in CEM/0 cell extracts as well (Figures S18–S19; Supporting Information).

In assays, Tri*PPP*ro-d4TTP **1a** (AB-C9; AB-C9) showed higher antiviral activities in wild-type CEM/0 cells (EC_50_ = 0.042 μM/HIV-1; EC_50_ = 0.066 μM/HIV-2) and in HIV-2-infected CEM/TK^–^ cells (EC_50_ = 0.29 μM/HIV-2) than the parent d4T. However, the inhibition of HIV-1 and HIV-2 replication by δ-biocleavable protected d4T4P **8** and **9** was lower than γ-(AB-C9; AB-C9)-d4TTP **1a** in CEM/0 and CEM/TK^–^ cells, which might be reasoned by the lower chemical and enzymatic stabilities of Tetra*PPPP*ro-d4T4Ps **8** and **9** compared to Tri*PPP*ro-d4TTP **1a**. Additionally, Tetra*PPPP*ro-prodrugs **8** and **9** exhibit poor cellular permeability because they are less lipophilic due to the additional negative charge as compared to Tri*PPP*ro-d4TTP **1a**.

The importance of lipophilicity can also be seen with compounds **20** and **24**. Both compounds were synthesized separately and then tested. Both compounds are additionally charged as compared to compounds **4**–**9**. Again, a compromised uptake would lead to low intracellular concentrations. Less uptake also means extracellular hydrolysis to d4T, which can be taken up and show activity in wild-type cells but not in CEM/TK^–^ cells.

As can be seen, Tetra*PPPP*ro-nucleotides **4b**, **5a**, **6**, and **7** exhibited significantly greater inhibition of HIV-2 replication compared to our previously reported **1a** in HIV-2-infected CEM/TK^–^ cells. More interestingly, the alkylated and biodegradable modified d4T tetraphosphate derivatives **6** and **7** showed similar or even higher values than Tri*PPP*ro-nucleotides **2a** and **2b**. In contrast, the antiviral activity of the double dialkylated d4T tetraphosphate derivatives **4** and **5** was found to be lower than the corresponding Tri*PPP*ro-nucleotides **3a** and **3b**. Taking our own test data, the SI values for prodrugs **6** (SI = 643) and **7** (SI = 4330) were significantly improved compared to the previously reported SI values for prodrugs **2a** (SI = 76) and **2b** (SI = 452) in HIV-2-infected CEM/0 cells. However, prodrugs **4d** (SI = 522) and **5a** (SI = 135) exhibited markedly lower SI values compared to prodrugs **3a** (SI = 938) and **3b** (SI = 1830), respectively.

## 3 Conclusions

Here, we disclose three different types of d4T tetraphosphate (d4T4P) derivatives **4**–**9** bearing modifications at the δ-position. All compounds were synthesized by using *H*-phosphonate and/or *H*-phosphinate chemistries. Interestingly, depending on the δ-modifications, different predominant products were formed in hydrolysis studies using different media. δ-Phosph(on)ate-modified-d4TTP compounds **4** and **5** bearing two *nonbioreversible* residues led selectively to the formation of d4T triphosphate (d4TTP) due to a pure chemical cleavage of the δ-phosphate or δ-phosphonate moiety, respectively. These derivatives showed very high hydrolytic stability. δ-(AB-C4; C18)-d4T4P **6** and δ-(AB-C4)-δ-C-(C18)-d4T4P **7** bear one biodegradable acyloxybenzyl moiety in combination with an alkyl-residue hydrolyzed by enzymatic cleavage to give δ-alkylated d4T4P derivatives **20** and **24** that proved to be highly stable toward further hydrolysis either by chemical or enzymatic means. Interestingly, such compounds acted as substrates for the HIV polymerase RT. Finally, δ-(AB-C9; AB-C9)-d4T4P **8** and δ-(AB-C4; ACB-C16)-d4T4P **9** comprising two biodegradable moieties, formed d4T tetraphosphate (d4T4P), which was also a substrate for HIV-RT but also prone to quick dephosphorylation to give d4TTP and d4TDP.

The compounds resulting in the formation of a δ-alkylated tetraphosphate derivative were found to be highly antivirally active also in TK-deficient CEM-cells (**6** (EC_50_ = 0.059 μM/HIV-2), and **7** (EC_50_ = 0.0065 μM/HIV-2; an improvement of up to 7700-fold as compared to the parent d4T). Particularly, the compounds bearing a phosphonate moiety in the δ-position proved, on the one hand, highly stable and, on the other hand, highly antivirally active as well. Interestingly, in primer extension assays, even the Tetra*PPPP*ro-nucleotides **4** were substrates for HIV-RT, as were the δ-monomasked compounds **20** and **24** and d4T4P. However, compounds **4**, **5**, **20**, and **24** were not substrates for human DNA polymerases α, β, and γ. In contrast, although not a substrate for DNA polymerases α and γ, d4T4P was a substrate for HIV-RT and was also a substrate for DNA-polymerase β, similar to d4TTP.

Hence, we are convinced that the Tetra*PPPP*ro technology has a high potential to be used in antiviral chemotherapies in the future. Highly active Tetra*PPPP*ro-prodrugs may be potentially clinically useful in the treatment of virus infections. Next, the pharmacokinetic properties of the lead compounds will be studied.

## 4 Experimental Section

General: All experiments were carried out under a nitrogen atmosphere and anhydrous conditions. HIV-RT was purchased from Roboklon, and human polymerases α, β, and γ were purchased from Chimerx. All anhydrous solvents (THF and CH_3_CN) were purchased from Acros Organics (Extra Dry over molecular sieves). Ultrapure water was produced by a Sartorius Aurium pro (Sartopore 0.2 μm, UV). HPLC-grade solvents (CH_3_CN and THF) were purchased from VWR. Phosphate buffer saline (PBS) and DEAE-Sephadex A-25 were purchased from Sigma-Aldrich. All other organic solvents (ethyl acetate, petroleum ether 50–70, CH_2_Cl_2_, acetone, and CH_3_OH) were purchased in technical grade and distilled prior to use. Commercially available reagents and solvents were used without further purification. General flash column chromatography was performed with silica gel 60 M (0.04–0.063 mm, Macherey-Nagel). For reversed-phase automated flash chromatography, an Interchim Puriflash 430 was used in combination with Chromabond Flash RS40C_18_ ec or RS 120C18 ec. Analytical thin-layer chromatography (TLC): For thin-layer chromatography, Macherey-Nagel precoated TLC sheets, Alugram Xtra SIL G/UV254, were used. All HPLC measurements were carried out using a VWR-Hitachi LaChromElite HPLC system (L-2130, L-2200, L-2455), EzChromElite software, and Agilent Technologies 1260 Infinity II with the following parts: 1260 Quat Pump VL, 1260 Vialsampler, 1260 DAD. TBAA buffer: 2 mM tetra-*n*-butylammonium acetate solution (pH 6.0). HPLC method: Nucleodur 100–5C18ec; 0–20 min: TBAA buffer/CH_3_CN gradient (5–80%); 20–30 min: buffer/CH_3_CN (80%); 30–33 min: buffer/CH_3_CN (80–5%); 33–38 min: buffer/CH_3_CN (5%); flow: 1 mL/min. All Tetra*PPPP*ro-compounds were analyzed for purity by NMR spectroscopy and RP-HPLC–UV. Unless otherwise indicated (prodrug **8** was 85%.), all compounds have a purity ≥95%. All NMR spectra were carried out using Bruker spectrometers: the Bruker AMX 400 and the Bruker AVIII 600. All chemical shifts (δ) were given in ppm and calibrated based on solvent signals. HRMS (ESI) mass spectra were performed with a VG Analytical Finnigan ThermoQuest MAT 95 XL or an Agilent 6224 EIS-TOF spectrometer. MALDI mass spectra were recorded on an ultrafleXtreme MALDI-TOF-TOF mass spectrometer by Bruker Daltonik with 9-AA as the matrix.

### 4.1 Syntheses and Characterization

The syntheses and characterization of *H*-phosphonate **10**,^55^*H*-phosphinate **11**,^55^*H*-phosphonates **12**,^49^*H*-phosphinate **13**,^50^*H*-phosphonate **14**,^44^ and *H*-phosphonate **15**(45) were described previously. The synthesis of d4TTP was described previously.^56^ D4TTP (*n*-Bu_4_N^+^ form) was eluted using a linear gradient of 0–1 M TEAB buffer and purified by automatic RP18 flash chromatography, followed by ion exchange with Dowex 50WX8 (H^+^), neutralization with tetra-*n*-butylammonium hydroxide, and a second RP18 flash chromatography purification step.

### 4.2 General Procedure: Preparation of Tetra*PPPP*ro-compounds 4–9

The reactions were performed in a nitrogen (N_2_) atmosphere under dry conditions. First, *H*-phosphonates **10**, **12**, **14**, and **15** and *H*-phosphinates **11** and **13** (0.3 mmol, 1.0 equiv) were dissolved in CH_3_CN or THF. *N*-chlorosuccinimide (NCS, 0.3 mmol, 1.0 equiv) was added, and the mixture was stirred for 2 h at room temperature. Subsequently, d4TTP (*n*-Bu_4_N^+^) (0.3 mmol, 1.0 equiv) in 6 mL of CH_3_CN were added, and the mixture was stirred for 4 h. All volatile components were removed in vacuum. The crude product was purified by automatic RP18 flash chromatography, followed by ion-exchange to the ammonium form with Dowex 50WX8 (NH_4_^+^) cation-exchange resin and a second RP18 chromatography purification step. The mixture compounds were freeze-dried, and the products **4**–**9** were obtained as white solids.

#### 4.2.1 (9H-Fluoren-9-yl)methyl Octadecylphosphonate 18

The reaction was carried out under a nitrogen (N_2_) atmosphere and dry conditions. *N*-methylmorpholine (11 mmol, 1.1 equiv) was added to a solution of octadecyl hydrogen phosphonate **17** (10 mmol, 1.0 equiv) and (9H-fluoren-9-yl)methyl carbonochloridate **16** (11 mmol, 1.1 equiv; 1 M solution in toluene) in dry diethyl ether and toluene. The reaction mixture was stirred for 30 min and cooled to 0 °C. The mixture was warmed to room temperature and stirred for 12 h. The solvent was removed in a vacuum, and the residue was purified using column chromatography to give nonsymmetric *H*-phosphonate **18**. Yield: 2.3 g (4.5 mmol, 45%) white solid. ^1^H NMR (400 MHz, CDCl_3_): δ [ppm] = 7.86 (d, ^1^*J*_HH_ = 687.3 Hz, 1H, PH), 7.77 (d, ^3^*J*_HH_ = 7.5 Hz, 2H, H-y), 7.62 (d, ^3^*J*_HH_ = 6.9 Hz, 2H, H-v), 7.42 (d, ^3^*J*_HH_ = 7.4 Hz, 2H, H-x), 7–35–7.32 (m, 2H, H-w), 4.45–4.38 (m, 2H, H-s), 4.28–4.22 (m, 1H, H-t), 4.00–3.90 (m, 2H, H-a), 1.64–1.58 (m, 2H, H-b), 1.36–1.20 (m, 30H, H-c, H-d, H-e, H-f, H-g, H-h, H-i, H-j, H-k, H-l, H-m, H-n, H-o, H-p, H-q), 0.88 (t, ^3^*J*_HH_ = 6.7 Hz, 3H, H-r). ^13^C NMR (101 MHz, CDCl_3_): δ (ppm) = 143.05, 143.01 (C-u), 141.38, 141,37 (C-z), 127.96, 127.93 (C-w), 127.17, 127.15 (C-x), 125.0 (C-v), 120.04, 120.02 (C-y), 67.1 (d, ^3^*J*_CP_ = 6.2 Hz, C-s), 65.8 (d, ^3^*J*_CP_ = 6.3 Hz, C-a), 48.1 (d, ^3^*J*_CP_ = 6.8 Hz, C-t), 31.9, 29.67, 29.63, 29.60, 29.5, 29.4, 29.3, 29.0, 22.7 (C-c, C-d, C-e, C-f, C-g, C-h, C-i, C-j, C-k, C-l, C-m, C-n, C-o, C-p, C-q), 30.3 (d, ^3^*J*_CP_ = 7.9 Hz, C-b), 14.1 (C-r). ^31^P NMR (162 MHz, CDCl_3_): δ [ppm] = 7.81. HRMS (ESI-TOF, *m*/*z*): C_32_H_49_O_3_P, [M + Na]^+^ 535.3311; found 535.2000.

#### 4.2.2 (9H-Fluoren-9-yl)methyl Octadecylphosphinate 22

The reaction was carried out under a nitrogen (N_2_) atmosphere and dry conditions. *N*-methylmorpholine (11 mmol, 1.1 equiv) was added to a solution of phosphonic acid **21** (10 mmol, 1.0 equiv) and (9H-fluoren-9-yl)methyl carbonochloridate **16** (11 mmol, 1.1 equiv; 1 M solution in toluene) in dry diethyl ether and toluene. The reaction mixture was stirred for 30 min and cooled to 0 °C. The mixture was warmed to room temperature and stirred for 12 h. The solvent was removed in a vacuum, and the residue was purified using column chromatography to give nonsymmetric *H*-phosphinates **22**. Yield: 2.8 g (5.6 mmol, 56%) white solid. ^1^H NMR (400 MHz, CDCl_3_): δ (ppm) = 7.86 (dt, ^1^*J*_HH_ = 531.3 Hz, ^4^*J*_HH_ = 1.9 Hz, 1H, PH), 7.77 (d, ^3^*J*_HH_ = 7.5 Hz, 2H, H-y), 7.59 (d, ^3^*J*_HH_ = 6.9 Hz, 2H, H-v), 7.41 (d, ^3^*J*_HH_ = 7.4 Hz, 2H, H-x), 7.32 (t, ^3^*J*_HH_ = 7.5 Hz, 2H, H-w), 4.60–4.50 (m, 1H, H-t), 4.32–4.20 (m, 2H, H-s), 1.80–1.62 (m, 2H, H-a), 1.54–1.40 (m, 2H, H-b), 1.38–1.20 (m, 30H, H-c, H-d, H-e, H-f, H-g, H-h, H-i, H-j, H-k, H-l, H-m, H-n, H-o, H-p, H-q), 0.88 (t, ^3^*J*_HH_ = 6.7 Hz, 3H, H-r). ^13^C NMR (101 MHz, CDCl_3_): δ [ppm] = 143.5, 142.9 (C-u), 141.44, 141,38 (C-z), 127.92, 127.85 (C-w), 127.14, 127.08 (C-x), 125.0, 124.8 (C-v), 120.02, 120.00 (C-y), 67.5 (d, ^3^*J*_CP_ = 7.0 Hz, C-s), 48.2 (d, ^3^*J*_CP_ = 6.4 Hz, C-t), 31.9, 30.4, 30.2, 29.66, 29.63, 29.62, 29.59, 29.54, 29.32, 29.25, 29.17, 29.05, 22.6 (C-b, C-c, C-d, C-e, C-f, C-g, C-h, C-i, C-j, C-k, C-l, C-m, C-n, C-o, C-p, C-q), 29.2, 28.3 (C-a), 20.5 (d, ^3^*J*_CP_ = 2.8 Hz, C-b), 14.1 (C-r). ^31^P NMR (162 MHz, CDCl_3_): δ [ppm] = 40.9. HRMS (ESI-TOF, *m*/*z*): C_32_H_49_O_2_P, [M + Na]^+^ 519.3362; found 519.1000.

#### 4.2.3 δ-(Alkyl-C4; alkyl-C4)-d4T4P 4a

According to the general procedure, a mixture of 58 mg (alkyl-C4; alkyl-C4)-*H*-phosphonate **10a** (0.3 mmol, 1.0 equiv), 40 mg NCS (0.3 mmol, 1.0 equiv), and 378 mg d4TTP 3.3 × *n*Bu_4_N^+^ salt (0.3 mmol, 1.0 equiv) was stirred for 4 h at room temperature. Yield: 53 mg (0.08 mmol, 25%) white solid. HPLC-UV analysis confirmed purity: >98%. *t*_*R*_ = 10.2 min ^1^H NMR (600 MHz, CD_3_OD): δ (ppm) = 7.68 (d, ^4^*J*_HH_ = 1.2 Hz, 1H, H-6), 6.95 (dt, ^3^*J*_HH_ = 3.4 Hz, ^4^*J*_HH_ = 1.5 Hz, 1H, H-1′), 6.57 (dt, ^3^*J*_HH_ = 6.1 Hz, ^4^*J*_HH_ = 1.7 Hz, 1H, H-3′), 5.88 (ddd, ^3^*J*_HH_ = 6.0 Hz, ^3^*J*_HH_ = 2.2 Hz, ^4^*J*_HH_ = 1.3 Hz, 1H, H-2′), 5.04–4.98 (m, 1H, H-4′), 4.34–4.10 (m, 6H, H-a, H-5′), 3.20 (q, ^3^*J*_HH_ = 7.4 Hz, 0.18H, HN(CH_2_CH_3_)_3_^+^), 1.92 (d, ^4^*J*_HH_ = 1.1 Hz, 3H, H-7), 1.75–1.60 (m, 4H, H-b), 1.54–1.35 (m, 4H, H-c), 1.31 (q, ^3^*J*_HH_ = 7.4 Hz, 0.24H, HN(CH_2_CH_3_)_3_^+^), 0.95 (t, ^3^*J*_HH_ = 7.4 Hz, 6H, H-d). ^13^C NMR (151 MHz, CD_3_OD): δ [ppm] = 166.7 (C-4), 152.9 (C-2), 138.7 (C-6), 136.0 (C-3′), 127.1 (C-2′), 112.0 (C-5), 90.9 (C-1′), 87.2 (d, ^3^*J*_CP_ = 9.2 Hz, C-4′), 69.4 (d, ^3^*J*_CP_ = 6.4 Hz, C-a), 67.8 (d, ^3^*J*_CP_ = 5.7 Hz, C-5′), 47.5 (HN(CH_2_CH_3_)_3_^+^), 33.3 (d, ^3^*J*_CP_ = 7.3 Hz, C-b), 19.7 (C-c), 14.0 (C-d), 12.5 (C-7), 9.1 [HN(CH_2_CH_3_)_3_^+^]. ^31^P NMR (243 MHz, CD_3_OD): δ [ppm]= −11.5 (d, ^2^*J*_pp_ = 17.6 Hz, *P*-δ), −12.7 (d, ^2^*J*_pp_ = 17.6 Hz, *P*-α), −22.9 (t, ^2^*J*_pp_ = 16.1 Hz, *P*-γ), −23.7 (t, ^2^*J*_pp_ = 16.1 Hz, *P*-β). MALDI-MS (*m*/*z*): calculated for C_18_H_32_N_2_O_16_P_4_ (M-H)^−^ 655.0630; found, 655.0571.

#### 4.2.4 δ-(Alkyl-C12; alkyl-C12)-d4T4P 4b

According to the general procedure, a mixture of 126 mg (alkyl-C12; alkyl-C12)-*H*-phosphonate **10b** (0.3 mmol, 1.0 equiv), 40 mg NCS (0.3 mmol, 1.0 equiv), and 378 mg d4TTP 3.3 × *n*Bu_4_N^+^ salt (0.3 mmol, 1.0 equiv) was stirred for 4 h at room temperature. Yield: 80 mg (0.08 mmol, 28%) white solid. HPLC-UV analysis confirmed purity: >96%. *t*_*R*_ = 14.7 min ^1^H NMR (600 MHz, CD_3_OD): δ [ppm] = 7.69 (d, ^4^*J*_HH_ = 1.2 Hz, 1H, H-6), 6.95 (dt, ^3^*J*_HH_ = 3.4 Hz, ^4^*J*_HH_ = 1.5 Hz, 1H, H-1′), 6.57 (dt, ^3^*J*_HH_ = 6.1 Hz, ^4^*J*_HH_ = 1.7 Hz, 1H, H-3′), 5.87 (ddd, ^3^*J*_HH_ = 6.0 Hz, ^3^*J*_HH_ = 2.2 Hz, ^4^*J*_HH_ = 1.4 Hz, 1H, H-2′), 5.02–4.98 (m, 1H, H-4′), 4.34–4.12 (m, 6H, H-a, H-5′), 3.20 (q, ^3^*J*_HH_ = 7.4 Hz, 1.32H, HN(CH_2_CH_3_)_3_^+^), 1.92 (d, ^4^*J*_HH_ = 1.1 Hz, 3H, H-7), 1.74–1.64 (m, 4H, H-b), 1.45–1.37 (m, 4H, H-c), 1.35–1.25 (m, 34H, H-d, H-e, H-f, H-j, H-h, H-i, H-j, H-k, HN(CH_2_CH_3_)_3_^+^), 0.90 (t, ^3^*J*_HH_ = 7.0 Hz, 6H, H-l). ^13^C NMR (151 MHz, CD_3_OD): δ [ppm] = 166.7 (C-4), 152.9 (C-2), 138.7 (C-6), 136.0 (C-3′), 127.1 (C-2′), 112.0 (C-5), 90.9 (C-1′), 87.2 (d, ^3^*J*_CP_ = 9.2 Hz, C-4′), 69.8 (d, ^3^*J*_CP_ = 6.2 Hz, C-a), 67.8 (d, ^3^*J*_CP_ = 5.7 Hz, C-5′), 47.5 [HN(CH_2_CH_3_)_3_^+^], 33.1 (C-b), 31.35, 31.30, 30.84, 30.81, 30.77, 30.76, 30.5, 30.3, 23.8 (C-d, C-e, C-f, C-g, C-h, C-i, C-j, C-k), 26.6 (C-c), 14.5 (C-l), 12.5 (C-7), 9.1 [HN(CH_2_CH_3_)_3_^+^]. ^31^P NMR (243 MHz, CD_3_OD): δ [ppm] = −11.5 (d, ^2^*J*_pp_ = 17.6 Hz, *P*-δ), −12.7 (d, ^2^*J*_pp_ = 17.6 Hz, *P*-α), −23.0 (t, ^2^*J*_pp_ = 17.6 Hz, *P*-γ), −23.8 (t, ^2^*J*_pp_ = 16.1 Hz, *P*-β). MALDI-MS (*m*/*z*): calculated for C_34_H_64_N_2_O_16_P_4_ (M-H)^−^ 879.3134; found: 879.3196.

#### 4.2.5 δ-(Alkyl-C18; alkyl-C18)-d4T4P 4c

According to the general procedure, a mixture of 176 mg (alkyl-C18; alkyl-C18)-*H*-phosphonate **10c** (0.3 mmol, 1.0 equiv), 40 mg NCS (0.3 mmol, 1.0 equiv), and 378 mg d4TTP 3.3 × *n*Bu_4_N^+^ salt (0.3 mmol, 1.0 equiv) was stirred for 4 h at room temperature. Yield: 21 mg (0.015 mmol, 5%) white solid. HPLC-UV analysis confirmed purity: >97%. *t*_*R*_ = 20.7 min ^1^H NMR (600 MHz, CD_3_OD): δ [ppm] = 7.72 (d, ^4^*J*_HH_ = 1.2 Hz, 1H, H-6), 6.96 (dt, ^3^*J*_HH_ = 3.4 Hz, ^4^*J*_HH_ = 1.7 Hz, 1H, H-1′), 6.55 (dt, ^3^*J*_HH_ = 6.0 Hz, ^4^*J*_HH_ = 1.7 Hz, 1H, H-3′), 5.85 (ddd, ^3^*J*_HH_ = 6.0 Hz, ^3^*J*_HH_ = 2.2 Hz, ^4^*J*_HH_ = 1.3 Hz, 1H, H-2′), 5.02–4.96 (m, 1H, H-4′), 4.34–4.10 (m, 6H, H-a, H-5′), 3.20 (q, ^3^*J*_HH_ = 7.4 Hz, 0.36H, HN(CH_2_CH_3_)_3_^+^), 1.92 (d, ^4^*J*_HH_ = 1.1 Hz, 3H, H-7), 1.73–1.63 (m, 4H, H-b), 1.45–1.37 (m, 4H, H-c), 1.45–1.25 (m, 56.6H, H-d, H-e, H-f, H-j, H-h, H-i, H-j, H-k, H-l, H-m, H-n, H-o, H-p, H-q, HN(CH_2_CH_3_)_3_^+^), 0.90 (t, ^3^*J*_HH_ = 6.8 Hz, 6H, H-r). ^13^C NMR (151 MHz, CD_3_OD): δ [ppm] = 166.7 (C-4), 152.9 (C-2), 138.8 (C-6), 136.0 (C-3′), 127.1 (C-2′), 112.1 (C-5), 90.9 (C-1′), 87.3 (d, ^3^*J*_CP_ = 9.2 Hz, C-4′), 69.6 (d, ^3^*J*_CP_ = 6.2 Hz, C-a), 67.8 (d, ^3^*J*_CP_ = 5.7 Hz, C-5′), 33.1 (C-b), 31.36, 31.31, 30.84, 30.82, 30.78, 30.77, 30.76, 30.5, 30.4, 23.8 (C-d, C-e, C-f, C-g, C-h, C-i, C-j, C-k, C-l, C-m, C-n, C-o, C-p, C-q), 26.7 (C–c), 14.5 (C-r), 12.5 (C-7). ^31^P NMR (243 MHz, CD_3_OD): δ [ppm] = −12.1 (d, ^2^*J*_pp_ = 19.1 Hz, *P*-δ), −12.9 (d, ^2^*J*_pp_ = 16.0 Hz, *P*-α), −24.0 – −25.0 (m, 2P, *P*-β, *P*-γ). MALDI-MS (*m*/*z*): calculated for C_46_H_88_N_2_O_16_P_4_ (M-H)^−^ 1047.5012; found: 1047.5390.

#### 4.2.6 δ-(Alkyl-C4; alkyl-C18)-d4T4P 4d

According to the general procedure, a mixture of 117 mg (alkyl-C4; alkyl-C18)-*H*-phosphonate **10d** (0.3 mmol, 1.0 equiv), 40 mg NCS (0.3 mmol, 1.0 equiv), and 378 mg d4TTP 3.3 × *n*Bu_4_N^+^ salt (0.3 mmol, 1.0 equiv) was stirred for 4 h at room temperature. Yield: 94 mg (0.1 mmol, 34%) white solid. HPLC-UV analysis confirmed purity: >95%. *t*_*R*_ = 14.5 min ^1^H NMR (600 MHz, CD_3_OD): δ [ppm] = 7.69 (d, ^4^*J*_HH_ = 1.3 Hz, 1H, H-6), 6.95 (dt, ^3^*J*_HH_ = 3.4 Hz, ^4^*J*_HH_ = 1.5 Hz, 1H, H-1′), 6.57 (dt, ^3^*J*_HH_ = 6.0 Hz, ^4^*J*_HH_ = 1.7 Hz, 1H, H-3′), 5.87 (ddd, ^3^*J*_HH_ = 6.0 Hz, ^3^*J*_HH_ = 2.2 Hz, ^4^*J*_HH_ = 1.3 Hz, 1H, H-2′), 5.02–4.98 (m, 1H, H-4′), 4.32–4.12 (m, 6H, H-a^1^, H-a^2^, H-5′), 3.20 (q, ^3^*J*_HH_ = 7.4 Hz, 1.26H, HN(CH_2_CH_3_)_3_^+^), 1.92 (d, ^4^*J*_HH_ = 1.1 Hz, 3H, H-7), 1.75–1.64 (m, 4H, H-b^1^, H-b^2^), 1.45–1.38 (m, 4H, H-c^1^, H-c^2^), 1.34–1.26 (m, 29.9H, H-d^2^, H-e, H-f, H-j, H-h, H-i, H-j, H-k, H-l, H-m, H-n, H-o, H-p, H-q, HN(CH_2_CH_3_)_3_^+^), 0.95 (t, ^3^*J*_HH_ = 7.4 Hz, 3H, H-d^1^), 0.90 (t, ^3^*J*_HH_ = 7.0 Hz, 3H, H-r). ^13^C NMR (151 MHz, CD_3_OD): δ [ppm] = 166.7 (C-4), 152.9 (C-2), 138.7 (C-6), 136.0 (C-3′), 127.0 (C-2′), 112.0 (C-5), 90.9 (C-1′), 87.2 (d, ^3^*J*_CP_ = 9.2 Hz, C-4′), 69.8, 69.4 (2 × d, ^3^*J*_CP_ = 6.4 Hz, ^3^*J*_CP_ = 6.4 Hz, C-a^1^, C-a^2^), 67.8 (d, ^3^*J*_CP_ = 5.7 Hz, C-5′), 47.5 [HN(CH_2_CH_3_)_3_^+^], 33.38, 33.33, 33.1, 31.32, 31.28, 30.79, 30.75, 30.72, 30.70, 30.5, 30.3, 23.7 (C-b^1^, C-b^2^, C-d^2^, C-e, C-f, C-g, C-h, C-i, C-j, C-k, C-l, C-m, C-n, C-o, C-p, C-q), 26.6 (C-c^2^), 19.8 (C-c^1^), 14.4 (C-r), 14.0 (C-d^1^), 12.5 (C-7), 9.1 [HN(CH_2_CH_3_)_3_^+^]. ^31^P NMR (243 MHz, CD_3_OD): δ [ppm] = −11.5 (d, ^2^*J*_pp_ = 17.6 Hz, *P*-δ), −14.7 (d, ^2^*J*_pp_ = 14.8 Hz, *P*-α), −22.8 (t, ^2^*J*_pp_ = 14.7 Hz, *P*-γ), −23.7 (t, ^2^*J*_pp_ = 14.7 Hz, *P*-β). MALDI-MS (*m*/*z*): calculated for C_32_H_60_N_2_O_16_P_4_ (M-H)^−^ 851.2821; found: 851.2781.

#### 4.2.7 δ-(Alkyl-C8; alkyl-C18)-d4T4P 4e

According to the general procedure, a mixture of 134 mg (alkyl-C4; alkyl-C18)-*H*-phosphonate **10e** (0.3 mmol, 1.0 equiv), 40 mg NCS (0.3 mmol, 1.0 equiv), and 378 mg d4TTP 3.3 × *n*Bu_4_N^+^ salt (0.3 mmol, 1.0 equiv) was stirred for 4 h at room temperature. Yield: 106 mg (0.11 mmol, 36%) white solid. HPLC-UV analysis confirmed purity: >96%. *t*_*R*_ = 15.2 min ^1^H NMR (400 MHz, CD_3_OD): δ [ppm] = 7.70 (s, 1H, H-6), 6.96 (dt, ^3^*J*_HH_ = 3.4 Hz, ^4^*J*_HH_ = 1.6 Hz, 1H, H-1′), 6.55 (dt, ^3^*J*_HH_ = 6.0 Hz, ^4^*J*_HH_ = 1.6 Hz, 1H, H-3′), 5.88 (ddd, ^3^*J*_HH_ = 6.1 Hz, ^3^*J*_HH_ = 2.2 Hz, ^4^*J*_HH_ = 1.6 Hz, 1H, H-2′), 5.02–4.96 (m, 1H, H-4′), 4.36–4.10 (m, 6H, H-a^1^, H-a^2^, H-5′), 3.20 (q, ^3^*J*_HH_ = 7.4 Hz, 1.38H, HN(CH_2_CH_3_)_3_^+^), 1.92 (d, ^4^*J*_HH_ = 1.0 Hz, 3H, H-7), 1.75–1.64 (m, 2H, H-b^2^), 1.62–1.54 (m, 1H, H-b^1^), 1.45–1.20 (m, 40H, H-c^1^, H-c^2^, H-d^1^, H-d^2^, H-e^1^, H-e^2^, H-f^2^, H-g^1^, H-g^2^, H-h^2^, H-i, H-j, H-k, H-l, H-m, H-n, H-o, H-p, H-q, HN(CH_2_CH_3_)_3_^+^), 0.96–0.86 (m, 9H, H-f^1^, H-h^1^, H-r). ^13^C NMR (101 MHz, CD_3_OD): δ [ppm] = 166.7 (C-4), 152.9 (C-2), 138.7 (C-6), 135.9 (C-3′),127.2 (C-2′), 112.1 (C-5), 90.9 (C-1′), 87.2 (d, ^3^*J*_CP_ = 9.1 Hz, C-4′), 71.5 (d, ^3^*J*_CP_ = 6.6 Hz, C-a^1^), 69.7 (d, ^3^*J*_CP_ = 6.1 Hz, C-a^2^), 67.9 (d, ^3^*J*_CP_ = 6.1 Hz, C-5′), 47.5 (HN(CH_2_CH_3_)_3_^+^), 41.4 (d, ^3^*J*_CP_ = 7.7 Hz, C-b^1^), 33.1, 31.39, 31.32, 31.1, 30.8, 30.7, 30.5, 30.4, 30.1, 24.33, 24.30, 24.1, 23.8 (C-b^2^, C-c^1^, C-d^1^, C-d^2^, C-e^1^, C-e^2^, C-f^2^, C-g^1^, C-g^2^, C-h^2^, C-i, C-j, C-k, C-l, C-m, C-n, C-o, C-p, C-q), 26.7 (C-c^2^), 14.5 (C-f^1^, C-r), 12.5 (C-7), 11.4 (C-h^1^), 9.1 (HN(CH_2_CH_3_)_3_^+^). ^31^P NMR (162 MHz, CD_3_OD): δ [ppm] = −11.5 (d, ^2^*J*_pp_ = 17.6 Hz, *P*-δ), −12.6 (d, ^2^*J*_pp_ = 17.4 Hz, *P*-α), −22.9 (t, ^2^*J*_pp_ = 17.6 Hz, *P*-γ), −23.6 (t, ^2^*J*_pp_ = 17.6 Hz, *P*-β). MALDI-MS (*m*/*z*): calculated for C_36_H_68_N_2_O_16_P_4_ [M-H]^−^ 907.3447; found: 907.3419.

#### 4.2.8 δ-(Alkyl-C4)-δ-C-(alkyl-C18)-d4T4P 5a

According to the general procedure, a mixture of 112 mg (alkyl-C4; alkyl-C18)-*H*-phosphinate **11a** (0.3 mmol, 1.0 equiv), 40 mg NCS (0.3 mmol, 1.0 equiv), and 378 mg d4TTP 3.3 × *n*Bu_4_N^+^ salt (0.3 mmol, 1.0 equiv) was stirred for 4 h at room temperature. Yield: 150 mg (0.17 mmol, 56%) white solid. HPLC-UV analysis confirmed purity: >95%. *t*_*R*_ = 14.4 min ^1^H NMR (400 MHz, CD_3_OD): δ [ppm] = 7.68 (d, ^4^*J*_HH_ = 1.3 Hz, 1H, H-6), 6.95 (dt, ^3^*J*_HH_ = 3.5 Hz, ^4^*J*_HH_ = 1.6 Hz, 1H, H-1′), 6.56 (dt, ^3^*J*_HH_ = 6.1 Hz, ^4^*J*_HH_ = 1.8 Hz, 1H, H-3′), 5.88 (ddd, ^3^*J*_HH_ = 6.0 Hz, ^3^*J*_HH_ = 3.2 Hz, ^4^*J*_HH_ = 1.8 Hz, 1H, H-2′), 5.02–4.98 (m, 1H, H-4′), 4.32–4.12 (m, 4H, H-5′, H-a^1^), 3.28–3.22 (m, 0.12H, H-A), 3.20 (q, ^3^*J*_HH_ = 7.4 Hz, 0.24H, HN(CH_2_CH_3_)_3_^+^), 2.05–1.95 (m, 2H, H-a^2^), 1.92 (d, ^4^*J*_HH_ = 1.1 Hz, 3H, H-7), 1.70–1.60 (m, 4.12H, H-b^1^, H-b^2^, H–B) 1.48–1.38 (m, 4.12H, H-c^1^, H-c^2^, H–C), 1.34–1.27 (m, 28.4H, H-d^2^, H-e, H-f, H-j, H-h, H-i, H-j, H-k, H-l, H-m, H-n, H-o, H-p, H-q, HN(CH_2_CH_3_)_3_^+^), 1.02 (t, ^3^*J*_HH_ = 7.6 Hz, 0.18H, H-D), 0.95 (dt, ^3^*J*_HH_ = 7.4 Hz, ^4^*J*_HH_ = 1.3 Hz, 3H, H-d^1^), 0.90 (t, ^3^*J*_HH_ = 7.0 Hz, 3H, H-r). ^13^C NMR (101 MHz, CD_3_OD): δ [ppm] = 166.6 (C-4), 152.8 (C-2), 138.7 (C-6), 135.9 (C-3′),127.1 (C-2′), 112.0 (C-5), 90.9 (C-1′), 87.2 (d, ^3^*J*_CP_ = 8.9 Hz, C-4′), 67.8 (d, ^3^*J*_CP_ = 5.5 Hz, C-5′), 67.0 (d, ^3^*J*_CP_ = 7.2 Hz, C-a^1^), 47.5 [HN(CH_2_CH_3_)_3_^+^], 33.1, 31.6, 31.5, 30.79, 30.75, 30.73, 30.6, 30.5, 30.3, 23.7 (C-c^2^, C-d^2^, C-e, C-f, C-g, C-h, C-i, C-j, C-k, C-l, C-m, C-n, C-o, C-p, C-q), 33.5 (d, ^3^*J*_CP_ = 6.5 Hz, C-b^1^), 27.4, 26.4 (C-a^2^), 23.4 (d, ^3^*J*_CP_ = 5.5 Hz, C-b^2^), 19.9 (C-c^1^), 14.4 (C-r), 14.0 (C-d^1^), 12.5 (C-7), 9.1 [HN(CH_2_CH_3_)_3_^+^]. ^31^P NMR (162 MHz, CD_3_OD): δ [ppm] = 24.4 (d, ^2^*J*_pp_ = 23.5 Hz, *P*-δ), −11.70 (d, ^2^*J*_pp_ = 17.6 Hz, *P*-α), −22.9 (t, ^2^*J*_pp_ = 17.7 Hz, *P*-γ), −23.6 (dd, ^2^*J*_pp_ = 23.5 Hz, ^2^*J*_pp_ = 17.7 Hz, *P*-β). MALDI-MS (*m*/*z*): calculated for C_32_H_60_N_2_O_15_P_4_ (M-H)^−^ 835.2871; found: 835.2901.

#### 4.2.9 δ-(Alkyl-C8)-δ-C-(alkyl-C18)-d4T4P 5b

According to the general procedure, a mixture of 112 mg (alkyl-C8; alkyl-C18)-*H*-phosphinate **11b** (0.3 mmol, 1.0 equiv), 40 mg NCS (0.3 mmol, 1.0 equiv), and 378 mg d4TTP 3.3 × *n*Bu_4_N^+^ salt (0.3 mmol, 1.0 equiv) was stirred for 4 h at room temperature. Yield: 111 mg (0.12 mmol, 39%) white solid. HPLC-UV analysis confirmed purity: >97%. *t*_*R*_ = 15.1 min ^1^H NMR (400 MHz, CD_3_OD): δ [ppm] = 7.69 (d, ^4^*J*_HH_ = 1.1 Hz, 1H, H-6), 6.95 (dt, ^3^*J*_HH_ = 3.5 Hz, ^4^*J*_HH_ = 1.6 Hz, 1H, H-1′), 6.57 (dt, ^3^*J*_HH_ = 6.0 Hz, ^4^*J*_HH_ = 1.6 Hz, 1H, H-3′), 5.87 (ddd, ^3^*J*_HH_ = 6.1 Hz, ^3^*J*_HH_ = 2.2 Hz, ^4^*J*_HH_ = 1.4 Hz, 1H, H-2′), 5.03–4.99 (m, 1H, H-4′), 4.32–3.98 (m, 4H, H-5′, H-a^1^), 3.28–3.22 (m, 0.08H, H-A), 3.20 (q, ^3^*J*_HH_ = 7.4 Hz, 0.18H, HN(CH_2_CH_3_)_3_^+^), 2.10–1.99 (m, 2H, H-a^2^), 1.92 (d, ^4^*J*_HH_ = 1.1 Hz, 3H, H-7), 1.70–1.60 (m, 2H, H-b^2^), 1.60–1.54 (m, 2.8H, H-b^1^, H–B) 1.45–1.25 (m, 38.3H, H–C, H-c^1^, H-c^2^, H-d^1^, H-d^2^, H-e^1^, H-e^2^, H-f^2^, H-g^1^, H-g^2^, H-h^2^, H-i, H-j, H-k, H-l, H-m, H-n, H-o, H-p, H-q, HN(CH_2_CH_3_)_3_^+^), 1.02 (t, ^3^*J*_HH_ = 7.4 Hz, 0.12H, H-D), 0.95–0.85 (m, 9H, H-h^1^, H-f^1^, H-r). ^13^C NMR (101 MHz, CD_3_OD): δ [ppm] = 166.7 (C-4), 152.9 (C-2), 138.7 (C-6), 136.0 (C-3′), 127.1 (C-2′), 112.0 (C-5), 90.9 (C-1′), 87.3 (d, ^3^*J*_CP_ = 8.9 Hz, C-4′), 69.0 (dd, ^2^*J*_CP_ = 17.5 Hz, ^3^*J*_CP_ = 8.0 Hz, C-a^1^), 67.8 (d, ^3^*J*_CP_ = 5.6 Hz, C-5′), 41.5 (d, ^3^*J*_CP_ = 7.3 Hz, C-b^1^), 33.1, 31.6, 31.5, 31.18, 31.15, 30.79, 30.76, 30.73, 30.54, 30.47, 30.3, 30.1, 30.0, 24.44, 24.37, 24.1, 23.7 (C-c^1^, C-c^2^, C-d^1^, C-d^2^, C-e^1^, C-e^2^, C-f^2^, C-g^1^, C-g^2^, C-h^2^, C-i, C-j, C-k, C-l, C-m, C-n, C-o, C-p, C-q), 27.4, 26.4 (C-a^2^), 23.4 (d, ^3^*J*_CP_ = 5.6 Hz, C-b^2^), 14.4, 11.45, 11.41 (C-h^1^, C-f^1^, C-r), 12.5 (C-7). ^31^P NMR (162 MHz, CD_3_OD): δ [ppm] = 24.4 (dd, ^2^*J*_pp_ = 23.5 Hz, ^3^*J*_pp_ = 5.9 Hz, *P*-δ), −11.5 (d, ^2^*J*_pp_ = 20.4 Hz, *P*-α), −22.8 (t, ^2^*J*_pp_ = 17.6 Hz, *P*-γ), −23.6 (dd, ^2^*J*_pp_ = 23.5 Hz, ^2^*J*_pp_ = 17.6 Hz, *P*-β). MALDI-MS (*m*/*z*): calculated for C_36_H_68_N_2_O_15_P_4_ (M-H)^−^ 891.3497; found: 891.3436.

#### 4.2.10 δ-(AB-C4; alkyl-C18)-d4T4P 6

According to the general procedure, a mixture of 157 mg (AB-C4; alkyl-C18)-*H*-phosphonate **12** (0.3 mmol, 1.0 equiv), 40 mg NCS (0.3 mmol, 1.0 equiv), and 378 mg d4TTP 3.3 × *n*Bu_4_N^+^ salt (0.3 mmol, 1.0 equiv) was stirred for 4 h at room temperature. Yield: 101 mg (0.1 mmol, 32%) white solid. HPLC-UV analysis confirmed purity: >95%. *t*_*R*_ = 15.3 min ^1^H NMR (400 MHz, CD_3_OD): δ [ppm] = 7.67 (d, ^4^*J*_HH_ = 1.0 Hz, 1H, H-6), 7.52–7.48 (m, 2H, H-c^1^), 7.12–7.06 (m, 2H, H-d^1^), 6.93 (dt, ^3^*J*_HH_ = 3.4 Hz, ^4^*J*_HH_ = 1.6 Hz, 1H, H-1′), 6.53 (dt, ^3^*J*_HH_ = 6.0 Hz, ^4^*J*_HH_ = 1.6 Hz, 1H, H-3′), 5.83 (ddd, ^3^*J*_HH_ = 6.1 Hz, ^3^*J*_HH_ = 2.2 Hz, ^4^*J*_HH_ = 1.6 Hz, 1H, H-2′), 5.25–5.18 (m, 2H, H-a^1^), 5.02–4.96 (m, 1H, H-4′), 4.36–4.08 (m, 4H, H-a^2^, H-5′), 3.28–3.22 (m, 0.32H, H-A), 3.20 (q, ^3^*J*_HH_ = 7.4 Hz, 0.6H, HN(CH_2_CH_3_)_3_^+^), 2.58 (t, ^3^*J*_HH_ = 7.3 Hz, 2H, H-g^1^), 1.91 (d, ^4^*J*_HH_ = 1.0 Hz, 3H, H-7), 1.73 (quint, ^3^*J*_HH_ = 7.4 Hz, 2H, H-h^1^), 1.65–1.55 (m, 2.32H, H–B, H-b^2^), 1.50–1.40 (m, 2.32H, H–C, H-i^1^), 1.35–1.20 (m, 30.9H, H-c^2^, H-d^2^, H-e^2^, H-f^2^, H-g^2^, H-h^2^, H-i^2^, H-j^2^, H-k, H-l, H-m, H-n, H-o, H-p, H-q, HN(CH_2_CH_3_)_3_^+^), 1.03 (t, ^3^*J*_HH_ = 7.6 Hz, 0.48H, H-D), 0.98 (t, ^3^*J*_HH_ = 7.4 Hz, 3H, H-j^1^), 0.90 (t, ^3^*J*_HH_ = 7.0 Hz, 3H, H-r). ^13^C NMR (101 MHz, CD_3_OD): δ [ppm] = 173.7 (C-f^1^), 166.6 (C-4), 152.8 (C-2), 152.4 (C-e^1^), 138.7 (C-6), 135.9 (C-3′), 135.0 (d, ^3^*J*_CP_ = 7.1 H_Z_, C-b^1^), 130.2 (C-c^1^), 127.6 (C-2′), 122.9 (C-d^1^), 112.0 (C-5), 90.9 (C-1′), 87.2 (d, ^3^*J*_CP_ = 8.9 H_Z_, C-4′), 70.3 (d, ^3^*J*_CP_ = 5.6 H_Z_, C-a^1^), 69.9 (d, ^3^*J*_CP_ = 6.0 H_Z_, C-a^2^), 67.9 (d, ^3^*J*_CP_ = 6.2 H_Z_, C-5′), 47.5 [HN(CH_2_CH_3_)_3_^+^], 34.8 (C-g^1^), 33.1, 31.24, 31.20, 30.82, 30.81, 30.80, 30.76, 30.74, 30.68, 30.5, 30.4, 30.3, 26.9, 26.5, 23.7 (C-c^2^, C-d^2^, C-e^2^, C-f^2^, C-g^2^, C-h^2^, C-i^2^, C-j^2^, C-k, C-l, C-m, C-n, C-o, C-p, C-q), 28.1 (C-h^1^), 24.8 (C–B), 23.3 (C-i^1^), 20.7 (C–C), 14.4 (C-r), 14.1 (C-j^1^), 13.9 (C–D), 12.5 (C-7), 9.1 [HN(CH_2_CH_3_)_3_^+^]. ^31^P NMR (162 MHz, CD_3_OD): δ [ppm] = −11.5 (d, ^2^*J*_pp_ = 17.5 Hz, *P*-δ), −12.9 (d, ^2^*J*_pp_ = 17.5 Hz, *P*-α), −23.0 (t, ^2^*J*_pp_ = 17.5 Hz, *P*-γ), −23.8 (t, ^2^*J*_pp_ = 16.2 Hz, *P*-β). MALDI-MS (*m*/*z*): calculated for C_40_H_66_N_2_O_18_P_4_ (M-H)^−^ 985.3188; found: 985.3105.

#### 4.2.11 δ-(AB-C4)-δ-C-(alkyl-C18)-d4T4P 7

According to the general procedure, a mixture of 153 mg (AB-C4; alkyl-C18)-*H*-phosphinate **13** (0.3 mmol, 1.0 equiv), 40 mg NCS (0.3 mmol, 1.0 equiv), and 378 mg d4TTP 3.3 × *n*Bu_4_N^+^ salt (0.3 mmol, 1.0 equiv) was stirred for 4 h at room temperature. Yield: 127 mg (0.12 mmol, 41%) white solid. HPLC-UV analysis confirmed purity: >95%. *t*_*R*_ = 15.2 min ^1^H NMR (400 MHz, CD_3_OD): δ [ppm] = 7.67 (d, ^4^*J*_HH_ = 1.0 Hz, 1H, H-6), 7.52–7.48 (m, 2H, H-c^1^), 7.12–7.06 (m, 2H, H-d^1^), 6.93 (dt, ^3^*J*_HH_ = 3.4 Hz, ^4^*J*_HH_ = 1.6 Hz, 1H, H-1′), 6.53 (dt, ^3^*J*_HH_ = 6.0 Hz, ^4^*J*_HH_ = 1.6 Hz, 1H, H-3′), 5.83 (ddd, ^3^*J*_HH_ = 6.1 Hz, ^3^*J*_HH_ = 2.2 Hz, ^4^*J*_HH_ = 1.6 Hz, 1H, H-2′), 5.28–5.18 (m, 2H, H-a^1^), 5.02–4.96 (m, 1H, H-4′), 4.32–4.15 (m, 2H, H-5′), 3.28–3.22 (m, 0.24H, H-A), 3.18 (q, ^3^*J*_HH_ = 7.4 Hz, 0.3H, HN(CH_2_CH_3_)_3_^+^), 2.58 (t, ^3^*J*_HH_ = 7.3 Hz, 2H, H-g^1^), 2.07–1.97 (m, 2H, H-a^2^), 1.91 (d, ^4^*J*_HH_ = 1.2 Hz, 3H, H-7), 1.71 (quint, ^3^*J*_HH_ = 7.4 Hz, 2H, H-h^1^), 1.68–1.52 (m, 2.24H, H–B, H-b^2^), 1.50–1.40 (m, 2.32H, H–C, H-i^1^), 1.38–1.20 [m, 30.5H, H-c^2^, H-d^2^, H-e^2^, H-f^2^, H-g^2^, H-h^2^, H-i^2^, H-j^2^, H-k, H-l, H-m, H-n, H-o, H-p, H-q, HN(CH_2_CH_3_)_3_^+^], 1.03 (t, ^3^*J*_HH_ = 7.6 Hz, 0.36H, H-D), 0.98 (t, ^3^*J*_HH_ = 7.4 Hz, 3H, H-j^1^), 0.90 (t, ^3^*J*_HH_ = 7.0 Hz, 3H, H-r). ^13^C NMR (101 MHz, CD_3_OD): δ [ppm] = 173.7 (C-f^1^), 166.6 (C-4), 152.8 (C-2), 152.2 (C-e^1^), 138.7 (C-6), 135.9 (C-3′), 135.6 (d, ^3^*J*_CP_ = 6.8 Hz, C-b^1^), 130.5 (C-c^1^), 127.1 (C-2′), 122.8 (C-d^1^), 112.0 (C-5), 90.9 (C-1′), 87.2 (d, ^3^*J*_CP_ = 9.1 Hz, C-4′), 68.0, 67.8 (2d, C-a^1^, C-5′), 47.5 [HN(CH_2_CH_3_)_3_^+^], 34.8 (C-g^1^), 31.5, 31.4, 30.80, 30.76, 30.73, 30.6, 30.5, 30.2, 23.7(C-c^2^, C-d^2^, C-e^2^, C-f^2^, C-g^2^, C-h^2^, C-i^2^, C-j^2^, C-k, C-l, C-m, C-n, C-o, C-p, C-q), 28.0 (C-h^1^), 27.5, 26.6 (C-a^2^), 24.8 (C–B), 23.2 (d, ^3^*J*_CP_ = 5.5 Hz, C-b^2^), 14.4 (C-r), 14.1 (C-j^1^), 13.9 (C–D), 12.5 (C-7), 9.1 [HN(CH_2_CH_3_)_3_^+^]. ^31^P NMR (162 MHz, CD_3_OD): δ [ppm] = 24.7 (d, ^2^*J*_pp_ = 23.5 Hz, *P*-δ), −11.4 (d, ^2^*J*_pp_ = 17.6 Hz, *P*-α), −22.7 (t, ^2^*J*_pp_ = 17.6 Hz, *P*-γ), −23.5 (dd, ^2^*J*_pp_ = 23.5 Hz, ^2^*J*_pp_ = 17.6 Hz, *P*-β). MALDI-MS (*m*/*z*): calculated for C_40_H_66_N_2_O_17_P_4_ (M-H)^−^ 969.3239; found: 969.3291.

#### 4.2.12 δ-(AB-C9; AB-C9)-d4T4P 8

According to the general procedure, a mixture of 181 mg (AB-C4; AB-C9)-*H*-phosphonate **14** (0.3 mmol, 1.0 equiv), 40 mg NCS (0.3 mmol, 1.0 equiv), and 378 mg d4TTP 3.3 × *n*Bu_4_N^+^ salt (0.3 mmol, 1.0 equiv) was stirred for 4 h at room temperature. Yield: 48 mg (0.04 mmol, 14%) white solid. HPLC-UV analysis confirmed purity: >85%. *t*_*R*_ = 15.2 min ^1^H NMR (600 MHz, CD_3_OD): δ [ppm] = 7.66 (d, ^4^*J*_HH_ = 1.2 Hz, 1H, H-6), 7.42–7.35 (m, 4H, H-c), 7.08–7.02 (m, 4H, H-d), 6.94–6.92 (m, 1H, H-1′), 6.50 (dt, ^3^*J*_HH_ = 6.0 Hz, ^4^*J*_HH_ = 1.6 Hz, 1H, H-3′), 5.80 (ddd, ^3^*J*_HH_ = 6.1 Hz, ^3^*J*_HH_ = 2.2 Hz, ^4^*J*_HH_ = 1.6 Hz, 1H, H-2′), 5.20–5.10 (m, 4H, H-a), 4.98–4.92 (m, 1H, H-4′), 4.30–4.12 (m, 2H, H-5′), 2.57 (t, ^3^*J*_HH_ = 7.4 Hz, 2H, H-g), 1.89 (d, ^4^*J*_HH_ = 1.0 Hz, 3H, H-7), 1.73 (quint, ^3^*J*_HH_ = 6.7 Hz, 4H, H–h), 1.46–1.25 (m, 24H, H-i, H-j, H-k, H-l, H-m, H-n), 0.90 (t, ^3^*J*_HH_ = 7.0 Hz, 6H, H-o). ^13^C NMR (151 MHz, CD_3_OD): δ [ppm] = 173.7 (C-f), 166.5 (C-4), 152.8 (C-2), 152.4 (C-e), 138.6 (C-6), 135.7 (C-3′), 134.9 (d, ^3^*J*_CP_ = 6.6 Hz, C-b), 130.5 (d, ^3^*J*_CP_ = 2.3 Hz, C–c), 127.2 (C-2′), 122.9 (C-d), 112.0 (C-5), 90.8 (C-1′), 87.1 (d, ^3^*J*_CP_ = 8.8 Hz, C-4′), 70.3 (dd, ^3^*J*_CP_ = 5.6 Hz, ^4^*J*_CP_ = 1.6 Hz, C-a), 67.9 (C-5′), 35.0 (C-g), 33.0, 30.58, 30.43, 30.42, 30.2, 23.7 (C-i, C-j, C-k, C-l, C-m, C-n), 26.0 (C-h), 14.4 (C-o), 12.5 (C-7). ^31^P NMR (243 MHz, CD_3_OD): δ [ppm] = −10.3 (d, ^2^*J*_pp_ = 17.6 Hz, *P*-δ), −11.7 (d, ^2^*J*_pp_ = 17.4 Hz, *P*-α), −21.8 (t, ^2^*J*_pp_ = 17.3 Hz, *P*-γ), −22.6 (t, ^2^*J*_pp_ = 16.1 Hz, *P*-β). MALDI-MS (*m*/*z*): calculated for C_44_H_64_N_2_O_20_P_4_ (M-H)^−^ 1063.2930; found: 1063.2846.

#### 4.2.13 δ-(AB-C4; ACB-C16)-d4T4P 9

According to the general procedure, a mixture of 194 mg (AB-C4; ACB-C16)-*H*-phosphonate **15** (0.3 mmol, 1.0 equiv), 40 mg NCS (0.3 mmol, 1.0 equiv), and 378 mg d4TTP 3.3 × *n*Bu_4_N^+^ salt (0.3 mmol, 1.0 equiv) was stirred for 4 h at room temperature. Yield: 84 mg (0.07 mmol, 24%) white solid. HPLC-UV analysis confirmed purity: >95%. *t*_*R*_ = 16.0 min ^1^H NMR (600 MHz, CD_3_OD): δ [ppm] = 7.64 (d, ^4^*J*_HH_ = 1.2 Hz, 1H, H-6), 7.42–7.37 (m, 4H, H-c^1^, H-c^2^), 7.16–7.12 (m, 2H, H-d^2^), 7.08–7.02 (m, 2H, H-d^1^), 6.94–6.90 (m, 1H, H-1′), 6.50 (dt, ^3^*J*_HH_ = 6.0 Hz, ^4^*J*_HH_ = 1.6 Hz, 1H, H-3′), 5.80 (ddd, ^3^*J*_HH_ = 6.1 Hz, ^3^*J*_HH_ = 2.2 Hz, ^4^*J*_HH_ = 1.6 Hz, 1H, H-2′), 5.24–5.14 (m, 4H, H-a^1^, H-a^2^), 4.98–4.94 (m, 1H, H-4′), 4.32–4.15 (m, 2H, H-5′), 4.22 (t, ^3^*J*_HH_ = 6.6 Hz, 2H, H-g^2^), 3.28–3.20 (m, 0.2H, H-A), 3.16 (q, ^3^*J*_HH_ = 7.4 Hz, 0.48H, HN(CH_2_CH_3_)_3_^+^), 2.58 (t, ^3^*J*_HH_ = 7.4 Hz, 2H, H-g^1^), 1.90 (d, ^4^*J*_HH_ = 1.0 Hz, 3H, H-7), 1.76–1.66 (m, 4.2H, H–B, H-h^1^, H-h^2^), 1.50–1.40 (m, 4.2H, H–C, H-i^1^, H-i^2^), 1.39–1.25 (m, 24.72H, H-j^2^, H-k, H-l, H-m, H-n, H-o, H-p, H-q, H-r, H-s, H-t, H-u, HN(CH_2_CH_3_)_3_^+^), 1.02 (t, ^3^*J*_HH_ = 7.3 Hz, 0.3H, H-D), 0.99 (t, ^3^*J*_HH_ = 7.4 Hz, 3H, H-j^1^), 0.90 (t, ^3^*J*_HH_ = 7.0 Hz, 3H, H-v). ^13^C NMR (151 MHz, CD_3_OD): δ [ppm] = 173.7 (C-f^1^), 166.6 (C-4), 155.1 (C-f^2^), 152.8 (C-2), 152.7 (C-e^2^), 152.4 (C-e^1^), 138.7 (C-6), 135.9 (C-3′), 135.2 (d, ^3^*J*_CP_ = 7.6 Hz, C-b^2^), 134.9 (d, ^3^*J*_CP_ = 6.6 Hz, C-b^1^), 130.58, 130.56, 129.65, 129.63 (C-c^1^, C-c^2^), 127.2 (C-2′), 122.9 (C-d^1^), 122.3 (C-d^2^), 112.0 (C-5), 90.9 (C-1′), 87.1 (d, ^3^*J*_CP_ = 8.8 Hz, C-4′), 70.5, 70.4 (C-a^1^, C-a^2^), 70.0 (C-g^2^), 67.8 (C-5′), 47.5 [HN(CH_2_CH_3_)_3_^+^], 34.8 (C-g^1^), 33.1, 30.79, 30.77, 30.75, 30.68, 30.63, 30.5, 30.3, 23.7 (C-j^2^, C-k, C-l, C-m, C-n, C-o, C-p, C-q, C-r, C-s, C-t, C-u), 29.7 (C-h^2^), 28.1 (C-h^1^), 26.8 (C-i^2^), 23.3 (C-i^1^), 14.5 (C-v), 14.1 (C-j^1^), 12.5 (C-7), 9.1 [HN(CH_2_CH_3_)_3_^+^]. ^31^P NMR (243 MHz, CD_3_OD): δ [ppm] = −10.0 (d, ^2^*J*_pp_ = 18.5 Hz, *P*-δ), −11.8 (d, ^2^*J*_pp_ = 16.2 Hz, *P*-α), −21.2 (t, ^2^*J*_pp_ = 17.2 Hz, *P*-γ), −22.2 (t, ^2^*J*_pp_ = 16.1 Hz, *P*-β). MALDI-MS (*m*/*z*): calculated for C_46_H_68_N_2_O_21_P_4_ (M-H)^−^ 1107.3192; found: 1107.3231.

#### 4.2.14 δ-(Alkyl-C18)-d4T4P 20

According to the general procedure, a mixture of 154 mg (Fm; alkyl-C18)-*H*-phosphonate **18** (0.3 mmol, 1.0 equiv), 40 mg NCS (0.3 mmol, 1.0 equiv), and 378 mg d4TTP 3.3 × *n*Bu_4_N^+^ salt (0.3 mmol, 1.0 equiv) was stirred for 4 h at room temperature. After the crude product was concentrated in a vacuum, the cleavage of the Fm-moiety was achieved in a mixture of 10 mL CH_3_CN/TEA (10:1) and then stirred for 3 h at room temperature. The counterion was exchanged to the ammonium form with Dowex 50WX8 ion-exchange resin and then purified with rp18 chromatography. Product-containing fractions were collected, and the organic solvent was evaporated. The remaining aqueous solutions were freeze-dried, and the product was obtained. Yield: 22 mg (0.02 mmol, 7%) white solid. HPLC-UV analysis confirmed purity: >95%. *t*_*R*_ = 13.3 min ^1^H NMR (400 MHz, CD_3_OD): δ [ppm] = 7.70 (d, ^4^*J*_HH_ = 1.0 Hz, 1H, H-6), 6.95 (dt, ^3^*J*_HH_ = 3.4 Hz, ^4^*J*_HH_ = 1.6 Hz, 1H, H-1′), 6.58 (dt, ^3^*J*_HH_ = 6.0 Hz, ^4^*J*_HH_ = 1.6 Hz, 1H, H-3′), 5.87 (ddd, ^3^*J*_HH_ = 6.1 Hz, ^3^*J*_HH_ = 2.2 Hz, ^4^*J*_HH_ = 1.6 Hz, 1H, H-2′), 5.05–4.96 (m, 1H, H-4′), 4.40–4.18 (m, 2H, H-5′), 4.05 (q, ^3^*J*_HH_ = 6.6 Hz, 2H, H-a), 3.20 (q, ^3^*J*_HH_ = 7.4 Hz, 14.8H, HN(CH_2_CH_3_)_3_^+^), 1.92 (d, ^4^*J*_HH_ = 1.0 Hz, 3H, H-7), 1.63 (quint, ^3^*J*_HH_ = 7.4 Hz, 2H, H-b), 1.45–1.20 (m, 52H, H-c, H-d, H-e, H-f, H-g, H–h, H-i, H-j, H-k, H-l, H-m, H-n, H-o, H-p, H-q, HN(CH_2_CH_3_)_3_^+^), 1.03 (t, ^3^*J*_HH_ = 7.6 Hz, 0.48H, H-D), 0.90 (t, ^3^*J*_HH_ = 7.0 Hz, 3H, H-r). ^13^C NMR (101 MHz, CD_3_OD): δ [ppm] = 166.7 (C-4), 152.9 (C-2), 138.7 (C-6), 135.9 (C-3′), 127.1 (C-2′), 112.1 (C-5), 90.9 (C-1′), 87.1 (d, ^3^*J*_CP_ = 9.1 Hz, C-4′), 68.0 (C-5′), 66.1 (C-a), 47.6 [HN(CH_2_CH_3_)_3_^+^], 33.1, 30.79, 30.76, 30.53, 30.47, 23.7 (C-d, C-e, C-f, C-g, C-h, C-i, C-j, C-k, C-l, C-m, C-n, C-o, C-p, C-q), 31.6 (d, ^3^*J*_CP_ = 7.9 Hz, C-b), 26.8 (C–c), 14.5 (C-r), 12.5 (C-7), 9.1 (HN(CH_2_CH_3_)_3_^+^). ^31^P NMR (162 MHz, CD_3_OD): δ [ppm] = −11.5 (d, ^2^*J*_pp_ = 17.5 Hz, *P*-δ), −12.9 (d, ^2^*J*_pp_ = 17.5 Hz, *P*-α), −23.3 – −24.5 (m, 2P, *P*-β, *P*-γ). MALDI-MS (*m*/*z*): calculated for C_28_H_52_N_2_O_16_P_4_ (M-H)^−^ 795.2195; found: 795.0643.

#### 4.2.15 δ-C-(Alkyl-C18)-d4T4P 24

According to the general procedure, a mixture of 149 mg (Fm; alkyl-C18)-*H*-phosphinate **22** (0.3 mmol, 1.0 equiv), 40 mg NCS (0.3 mmol, 1.0 equiv), and 378 mg d4TTP 3.3 × *n*Bu_4_N^+^ salt (0.3 mmol, 1.0 equiv) was stirred for 4 h at room temperature. After the crude product was concentrated in a vacuum, the cleavage of the Fm-moiety was achieved in a mixture of 10 mL CH_3_CN/TEA (4:1) and then stirred for 4 h at room temperature. The counterion was exchanged to the ammonium form with Dowex 50WX8 ion-exchange resin and then purified with rp18 chromatography. Product-containing fractions were collected, and the organic solvent was evaporated. The remaining aqueous solutions were freeze-dried, and the product was obtained. Yield: 65 mg (0.07 mmol, 22%) white solid. HPLC-UV analysis confirmed purity: >99%. *t*_*R*_ = 13.2 min ^1^H NMR (400 MHz, CD_3_OD): δ [ppm] = 7.71 (d, ^4^*J*_HH_ = 1.0 Hz, 1H, H-6), 6.95 (dt, ^3^*J*_HH_ = 3.4 Hz, ^4^*J*_HH_ = 1.6 Hz, 1H, H-1′), 6.58 (dt, ^3^*J*_HH_ = 6.0 Hz, ^4^*J*_HH_ = 1.6 Hz, 1H, H-3′), 5.86 (ddd, ^3^*J*_HH_ = 6.1 Hz, ^3^*J*_HH_ = 2.2 Hz, ^4^*J*_HH_ = 1.6 Hz, 1H, H-2′), 5.04–4.98 (m, 1H, H-4′), 4.38–4.15 (m, 2H, H-5′), 3.28–3.22 (m, 3.84H, H-A), 3.18 (q, ^3^*J*_HH_ = 7.4 Hz, 1.5H, HN(CH_2_CH_3_)_3_^+^), 1.92 (d, ^4^*J*_HH_ = 1.2 Hz, 3H, H-7), 1.85–1.75 (m, 2H, H-a), 1.70–1.60 (m, 5.84H, H–B, H-b), 1.48–1.38 (m, 5.84H, H–C, H-c), 1.38–1.20 [m, 30.25H, H-d, H-e, H-f, H-g, H–h, H-i, H-j, H-k, H-l, H-m, H-n, H-o, H-p, H-q, HN(CH_2_CH_3_)_3_^+^], 1.03 (t, ^3^*J*_HH_ = 7.6 Hz, 5.76H, H-D), 0.90 (t, ^3^*J*_HH_ = 7.0 Hz, 3H, H-r). ^13^C NMR (101 MHz, CD_3_OD): δ [ppm] = 166.7 (C-4), 152.9 (C-2), 138.8 (C-6), 136.1 (C-3′), 127.0 (C-2′), 112.0 (C-5), 90.9 (C-1′), 87.3 (d, ^3^*J*_CP_ = 9.1 Hz, C-4′), 66.7 (d, ^3^*J*_CP_ = 5.7 Hz, C-5′), 59.5 (t, ^3^*J*_CP_ = 2.8 Hz, C-A), 47.4 [HN(CH_2_CH_3_)_3_^+^], 33.1, 32.4, 32.3, 30.84, 30.82, 30.79, 30.75, 30.56, 30.47, 30.3, 23.7(C–c, C-d, C-e, C-f, C-g, C-h, C-i, C-j, C-k, C-l, C-m, C-n, C-o, C-p, C-q), 27.1 (C-a), 24.8 (C–B), 24.5 (d, ^3^*J*_CP_ = 4.5 Hz, C-b^2^), 20.7 (t, ^4^*J*_CP_ = 1.7 Hz, C–C), 14.4 (C-r), 13.9 (C–D), 12.5 (C-7), 9.1 [HN(CH_2_CH_3_)_3_^+^]. ^31^P NMR (162 MHz, CD_3_OD): δ [ppm] = 19.5 (d, ^2^*J*_pp_ = 23.5 Hz, *P*-δ), −11.5 (d, ^2^*J*_pp_ = 17.6 Hz, *P*-α), −22.2 – −22.8 (m, 2P, *P*-β, *P*-γ). MALDI-MS (*m*/*z*): calculated for C_28_H_52_N_2_O_15_P_4_ (M-H)^−^ 779.2245; found: 779.0970.

#### 4.2.16 D4T4P

Method A. According to the general procedure, a mixture of 132 mg (Fm; Fm)-*H*-phosphonate **26** (0.3 mmol, 1.0 equiv), 40 mg NCS (0.3 mmol, 1.0 equiv), and 378 mg d4TTP 3.3 × *n*Bu_4_N^+^ salt (0.3 mmol, 1.0 equiv) was stirred for 4 h at room temperature. Subsequently, TEA (10 equiv) was added, and the reaction mixture was stirred at room temperature for 10 min. The crude product was purified by automated RP-flash chromatography using an CH_3_CN gradient in H_2_O. After freeze-drying of the product-containing fractions (mono-Fm-protected nucleoside 5′-tetraphosphate and di-Fm-protected nucleoside 5′-tetraphosphate), the residue was redissolved in a mixture of 10 mL CH_3_CN/TEA (4:1) and then stirred for 24 h at room temperature. Then, the solvent was evaporated, and the crude product was purified by automated RP-flash chromatography with an CH_3_CN gradient in H_2_O. Subsequently, the product was eluted using a linear gradient of 0–1 M TEAB, and the fractions containing the product were pooled, evaporated, and coevaporated with H_2_O. Yield: 46 mg (0.06 mmol, 19%) white solid. HPLC-UV analysis confirmed purity: >95%. *t*_*R*_ = 10.2 min.

Method B. To a stirred solution of 0.22 g d4T (1 mmol, 1.0 equiv), 0.21 g proton sponge (1 mmol, 1.0 equiv), and 5 mL trimethylphosphate at −5 °C, 0.14 mL phosphorousoxychloride (1.5 mmol, 1.5 equiv) was added, and the mixture was stirred for 10 min. Another portion of 0.14 mL phosphorus oxychloride (1.5 mmol, 1.5 equiv) was added to the reaction mixture and was further stirred for 20 min. Then, the mixture containing 1.85 g tetra-*n*-butylammonium triphosphate (2.5 mmol, 1.5 equiv), 1.3 mL tributylamine (5.5 mmol, 5.5 equiv), and 10 mL CH_3_CN was added to the reaction mixture and kept under stirring for 10 min. The reaction mixture was quenched by the slow addition of 100 mL of water, followed by extraction with dichloromethane (3 × 150 mL). After the crude product was concentrated in a vacuum, the desired product was eluted using a linear gradient of 0–1 M TEAB, and the fractions containing the product were pooled, evaporated, and coevaporated with H_2_O. Yield: 24 mg (0.03 mmol, 3%) white solid. ^1^H NMR (400 MHz, CD_3_OD): δ [ppm] = 7.71 (d, ^4^*J*_HH_ = 1.0 Hz, 1H, H-6), 6.95 (dt, ^3^*J*_HH_ = 3.4 Hz, ^4^*J*_HH_ = 1.6 Hz, 1H, H-1′), 6.58 (dt, ^3^*J*_HH_ = 6.0 Hz, ^4^*J*_HH_ = 1.6 Hz, 1H, H-3′), 5.86 (ddd, ^3^*J*_HH_ = 6.1 Hz, ^3^*J*_HH_ = 2.2 Hz, ^4^*J*_HH_ = 1.6 Hz, 1H, H-2′), 5.04–4.98 (m, 1H, H-4′), 4.36–4.15 (m, 2H, H-5′), 3.20 (q, ^3^*J*_HH_ = 7.3 Hz, 13.86H, HN(CH_2_CH_3_)_3_^+^), 1.92 (d, ^4^*J*_HH_ = 1.2 Hz, 3H, H-7), 1.31 (t, ^3^*J*_HH_ = 7.3 Hz, 20.79H, HN(CH_2_CH_3_)_3_^+^). ^13^C NMR (101 MHz, CD_3_OD): δ [ppm] = 166.7 (C-4), 152.9 (C-2), 138.8 (C-6), 136.0 (C-3′), 127.1 (C-2′), 112.1 (C-5), 90.9 (C-1′), 87.2 (d, ^3^*J*_CP_ = 9.1 Hz, C-4′), 67.8 (C-5′), 47.5 [HN(CH_2_CH_3_)_3_^+^], 12.5 (C-7), 9.1 (HN(CH_2_CH_3_)_3_^+^). ^31^P NMR (162 MHz, CD_3_OD): δ [ppm] = −10.3 (d, ^2^*J*_pp_ = 21.1 Hz, *P*-δ), −12.0 (d, ^2^*J*_pp_ = 17.8 Hz, *P*-α), −22.6 (t, ^2^*J*_pp_ = 17.6 Hz, *P*-γ), −23.7 (t, ^2^*J*_pp_ = 19.1 Hz, *P*-β). MALDI-MS (*m*/*z*): calculated for C_10_H_16_N_2_O_16_P_4_ (M-H)^−^ 542.9378; found: 542.8568.

### 4.3 Chemical Hydrolysis of Tetra*PPPP*ro-compounds 4–9 and Monomasked Compounds 20 and 24

Stock solutions (50 mM in DMSO) of compounds **4**–**9**, **20**, and **24** were prepared. 1.9 mM hydrolysis solutions of compounds **4**–**9**, **20**, and **24** were prepared from 22 μL of 50 mM solutions, 378 μL of DMSO, 200 μL of Milli-Q water, and 600 μL of phosphate buffered saline (PBS, pH 7.3 or 8.0). The solution was incubated at 37 °C in a thermomixer. 40 mL for each extraction and stored at −30 °C. When testing, samples were warmed up to room temperature, and 25 or 20 μL of the liquid was taken and injected into the analytical RP-18-HPLC instrument. Further aliquots were taken for monitoring the kinetic hydrolysis. The exponential decay curves were calculated with OriginPro 9.0G and yielded the half-lives (*t*_1/2_) of the compounds via one determination.

### 4.4 Preparation of Citrate Buffer, pH 2.0

5.88 g of citric acid and 3.58 g of NaCl were dissolved in 82 mL of hydrochloric acid (0.1 mol/L). Then, sodium hydroxide was added to adjust the pH to 2.0.

### 4.5 Preparation of Cell Extracts

CEM cells were grown in RPMI-1640-based cell culture medium to a final density of ∼3 × 10^6^ cells/mL. Cells were centrifuged for 10 min at 1250 rpm at 4 °C and washed twice with cold PBS, and the pellet was resuspended at 10^8^ cells/mL and sonicated (Hielscher Ultrasound Techn., 100% amplitude, 3·times for 10 s) to destroy cell integrity. Next, the resulting cell suspension was centrifuged at 10,000 rpm to remove cell debris. Finally, the supernatant was divided into aliquots and frozen at −80 °C.

### 4.6 Preparation of Human Plasma

Fresh human plasma was obtained from citrate (0.106 mol/L) and lithium heparin (25 IU/mL) anticoagulated whole blood samples upon centrifugation (3,000*g* for 10 min at RT). Finally, the supernatant was divided into aliquots and frozen at −80 °C.

#### 4.6.1 Enzymatic Hydrolysis of Tetra*PPPP*ro-compounds 4–9, 20, and 24 with PLE

10 μL of 50 mM DMSO stock solutions of compounds **4**–**9**, **20**, and **24** were diluted to 6.0 mM hydrolysis solutions by the addition of 31.7 μL of DMSO and 41.7 μL of ultrapure water. Then, 833 μL of 50 mM phosphate buffer (pH 7.3) and 125 μL of DMSO were added to the 6.0 mM hydrolysis solutions. The reaction was started by addition of 62.5 μL of PLE in phosphate buffer (100 units/mL), and the mixture was incubated at 800 rpm at 37 °C in a thermomixer. (1). At different times, aliquots (75 μL) were taken and stopped by the addition of 79.6 mL of MeOH. The mixture was stored in liquid nitrogen. When testing, samples were warmed up to room temperature, and 60 μL of the liquid was directly injected into HPLC analysis. (2). 40 μL for each extraction was stored in liquid nitrogen. When testing, samples were warmed up to room temperature, and 30 μL of the liquid was taken and injected into the analytical RP-18-HPLC instrument. The calculation of *t*_1/2_ was performed analogously to that for the chemical hydrolysis studies.

### 4.7 Enzyme-Catalyzed Hydrolysis of Tetra*PPPP*ro-compounds 4–9,20,24 in CEM/0 Cell Extracts and Human Citrate Plasma

6.0 mM hydrolysis solutions of compounds **4**–**9** were prepared from 21 μL of 50 mM DMSO stock solutions and 154 μL of DMSO. Two different samples, including 10 μL of water and 10 μL of 6.0 mM hydrolysis solution, were prepared in 2 mL Eppendorf vials. Next, 50 μL of human CEM cell extracts or 50 μL of human citrate plasma were added to the mixture, the reaction was started, and the mixture was incubated at 37 °C for different time periods. The reactions were stopped by the addition of 150 μL of MeOH. The resulting suspension was kept on ice for 5 min, followed by defrosting and centrifugation at 14,000 rpm (Heraeus, Biofuge Pico) for 22 min. The supernatants (80 or 60 μL) were directly injected into HPLC. The calculation of *t*_1/2_ was performed analogously to that for the chemical hydrolysis studies.

#### 4.7.1 Anti-HIV Replication Assay

Inhibition of HIV-1(HE)- and HIV-2(ROD)-induced cytopathogenicity in wild-type CEM/0 CEM CD4^+^ T-cells and thymidine kinase-deficient CEM/TK^–^ cell cultures was measured in microtiter 96-well plates containing ∼3 × 10^5^ CEM cells/mL infected with 100 CCID_50_ of HIV per milliliter and containing appropriate dilutions of the test compounds. After 4–5 days of incubation at 37 °C in a CO_2_-controlled humidified atmosphere, virus-induced cellular effects and syncytia cell formation were examined microscopically. The EC_50_ (50% effective concentration) was defined as the compound concentration required to inhibit HIV-induced giant cell formation by 50%.

#### 4.7.2 Primer-Extension Assays

HIV-RT and human polymerases α, β, and γ were bought from Roboklon and Chimerx. The fluorescent-labeled primer and template were obtained from Metabion. The gel size was adjusted to the electrophoresis chamber (450 × 200 × 1.0 mm).

Primer sequence for the Cy3-fluorescent-labeled primer extension experiment:
25nt primer sequence: 5′-Cy3-CGTTG GTCCT GAAGG AGGAT AGGTT-3′
30nt template sequence: 3′-GCAAC CAGGA CTTCC TCCTA TCCAA AGACA-5′
The following conditions were used in the primer extension experiments:

For the annealing of the primer/template mixture, 40 μL of the 10 μM primer and 60 μL of the 10 μM template were incubated for 5 min at 95 °C. Then, for optimal annealing, the temperature was reduced over a period of 2 h to 20 °C and incubated again for 20 min at 20 °C.

For the primer extension, the reaction mixture was incubated for 30 min for the HIV-RT, 60 min for the human DNA polymerase α, and 120 min for the human DNA polymerases β and γ at 37 °C, followed by 7 min at 80 °C. For the gel, 2 μL of loading dye (6X DNA from Thermo Scientific) was added to the reaction mixture, analyzed by denaturing PAGE (15%), and visualized via fluorescence.

## Supporting Information Available

Molecular formula strings of all presented compounds (CSV)
PBS hydrolysis of compounds **4d**, **4e**, **5a**, **5b**, **d4TTP**, d4TDP, **6**, **7**, **8**, **9**, and **d4T4P**, PLE hydrolysis of prodrugs **5a** and **8**, CEM/0 cell extracts hydrolysis of prodrugs **5a**, **6**, **7**, **8**, **20**, and **24**, and human plasma hydrolysis of prodrugs **5a**, **24**, and **8**, and NMR-spectra and HPLC-data of all presented compounds **4–9**, **20**, **24**, and **d4T4P** (PDF)

## Abbreviations

AB: acyloxybenzyl
ACB: alkoxycarbonyloxybenzyl
CC_50_: 50% cytotoxic concentration
Di*PP*ro: nucleoside diphosphate prodrugs
d4T: 3′-deoxy-2′,3′-didehydrothymidine
d4T4P: 2′,3′-didehydro-2′,3′-dideoxythymidine-5′-tetraphosphate
DPP: diphenyl hydrogen phosphonate
EC_50_: 50% effective concentration
HPLC: High-Performance Liquid Chromatography
HIV-RT: HIV reverse transcriptase
NCS: *N*-chlorosuccinimide
NDP: nucleoside diphosphate
NMP: nucleoside monophosphate
NTP: nucleoside triphosphate
NRTI: nucleoside reverse transcriptase inhibitor
PBS: phosphate buffered saline
PLE: pig liver esterase
RdRp: RNA-dependent RNA polymerase
TMP: trimethyl phosphate
Tri*PPP*ro: nucleoside triphosphate prodrugs
Tetra*PPPP*ro: nucleoside tetraphosphate prodrugs
TK: thymidine kinase
TMP-K: thymidylate kinase
