# Supplementary material for: Antiviral Activity of Lipophilic Nucleoside Tetraphosphate Compounds
Source: J Med Chem. 2024 Feb 12;67(4):2864–83. doi: 10.1021/acs.jmedchem.3c02022 (PMC10895676; doi:10.1021/acs.jmedchem.3c02022)
Supplement: Supplementary file 2 — jm3c02022_si_002.pdf [file jm3c02022_si_002.pdf]

Supporting Information

Antiviral Activity of Lipophilic Nucleoside Tetraphosphate Compounds

Xiao Jia,[1] *Dominique Schols,*[2] *Chris Meier*[1*]

[1]Organic Chemistry, Department of Chemistry, Faculty of Mathematics, Informatics and Natural Sciences, Universität Hamburg, Martin-Luther-King-Platz 6, D-20146 Hamburg, Germany.

[2]Laboratory of Virology and Chemotherapy, Department of Microbiology and Immunology and Transplantation, Rega Institute for Medical Research, KU Leuven, Herestraat 49, B-3000 Leuven, Belgium.

Corresponding authors email: chris.meier@uni-hamburg.de

Contents

Figure S1-S22. HPLC profiles of compounds **4-9**,**20**,**24** after incubation in PBS (pH 7.3), PLE, CEM cell extracts and citrate-stabilized human plasma............................S2

Spectral Data for New Compounds and HPLC Data....S9

Figure S1-S22. HPLC profiles of compounds **4-9**,**20**,**24** after incubation in PBS (pH 7.3), PLE, CEM cell extracts and citrate-stabilized human plasma.

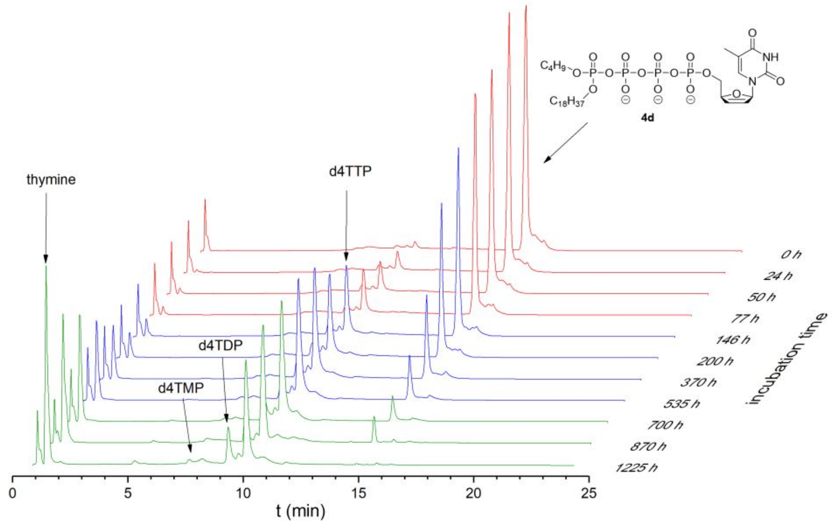

Figure S1. HPLC profiles of **4d** after incubation in PBS (pH 7.3).

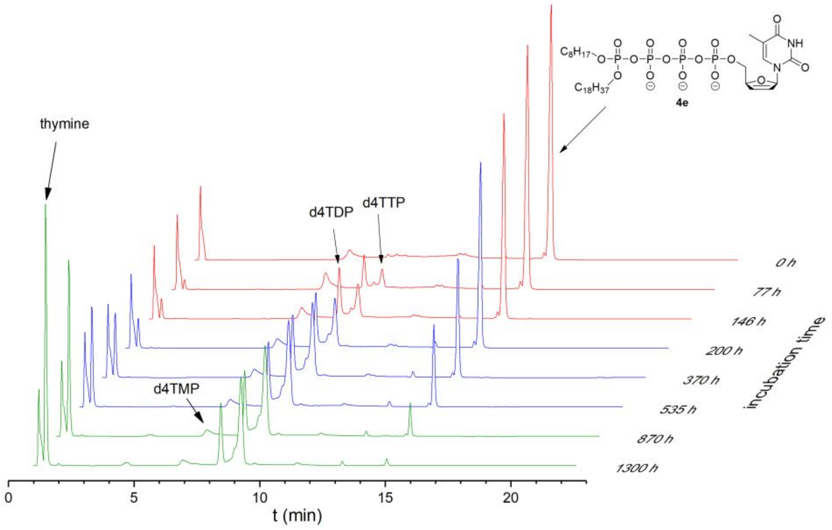

Figure S2. HPLC profiles of **4e** after incubation in PBS (pH 7.3).

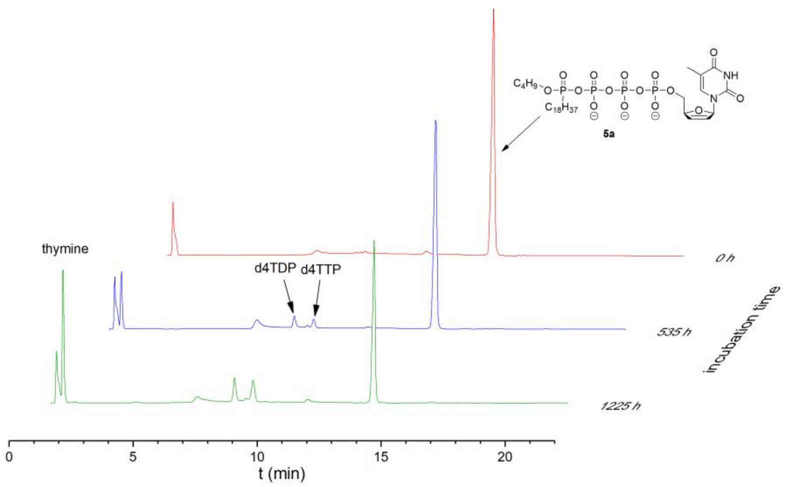

Figure S3. HPLC profiles of **5a** after incubation in PBS (pH 7.3).

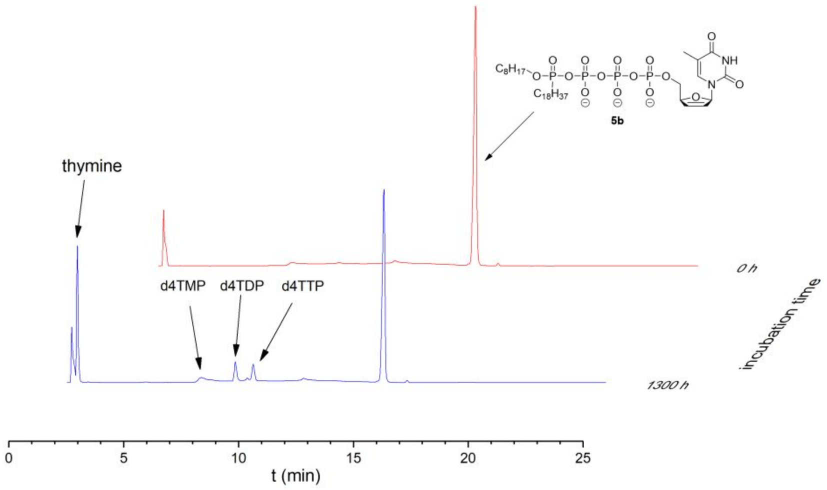

Figure S4. HPLC profiles of **5b** after incubation in PBS (pH 7.3).

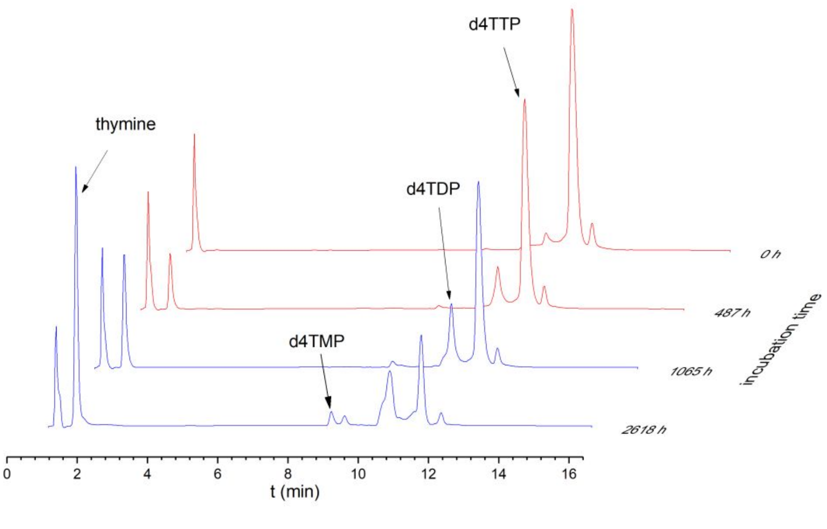

Figure S5. HPLC profiles of d4TTP after incubation in PBS (pH 7.3).

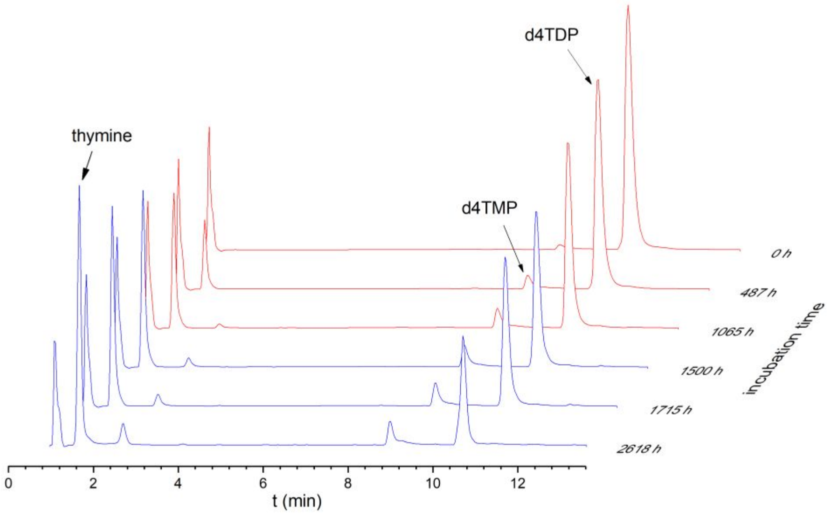

Figure S6. HPLC profiles of d4TDP after incubation in PBS (pH 7.3).

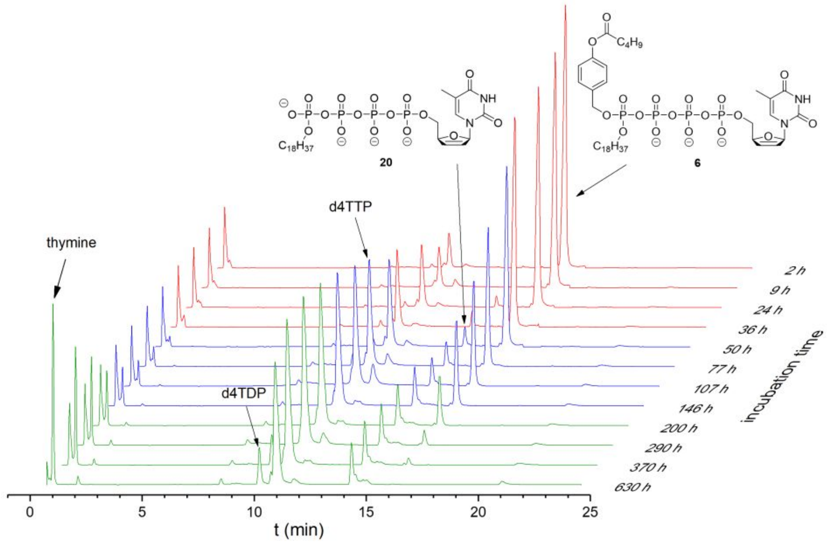

Figure S7. HPLC profiles of **6** after incubation in PBS (pH 7.3).

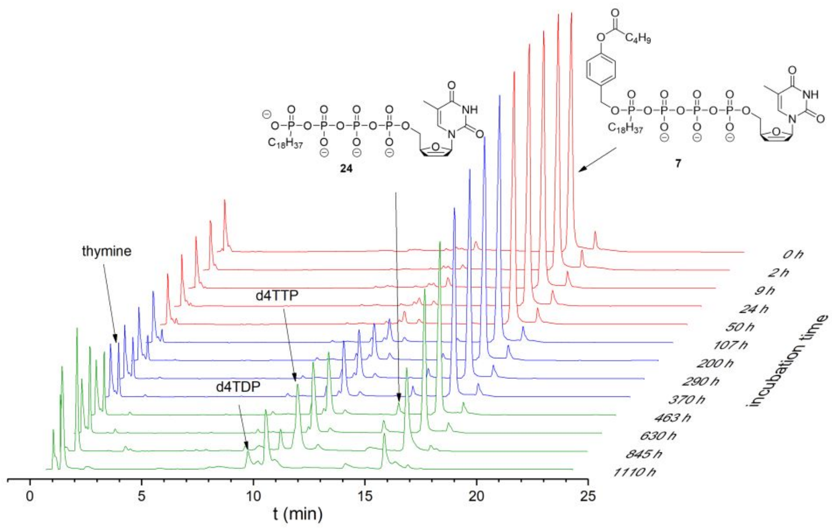

Figure S8. HPLC profiles of **7** after incubation in PBS (pH 7.3).

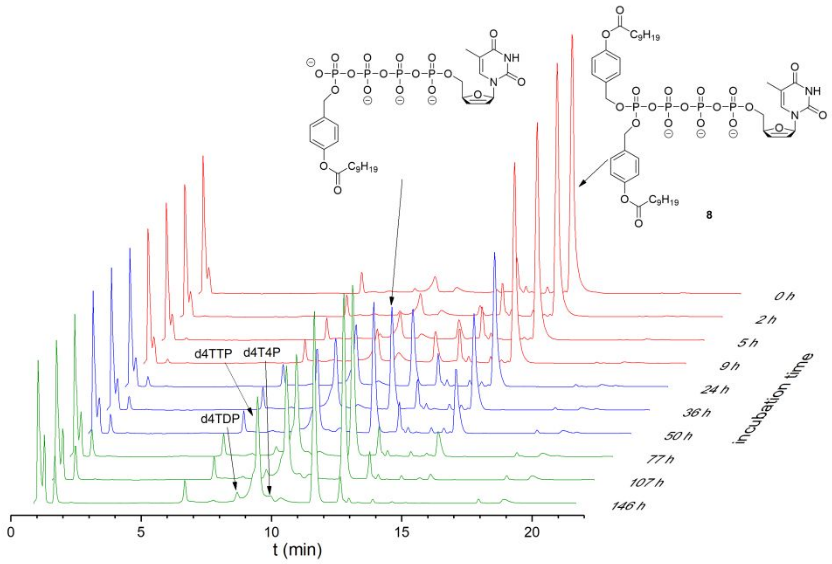

Figure S9. HPLC profiles of **8** after incubation in PBS (pH 7.3).

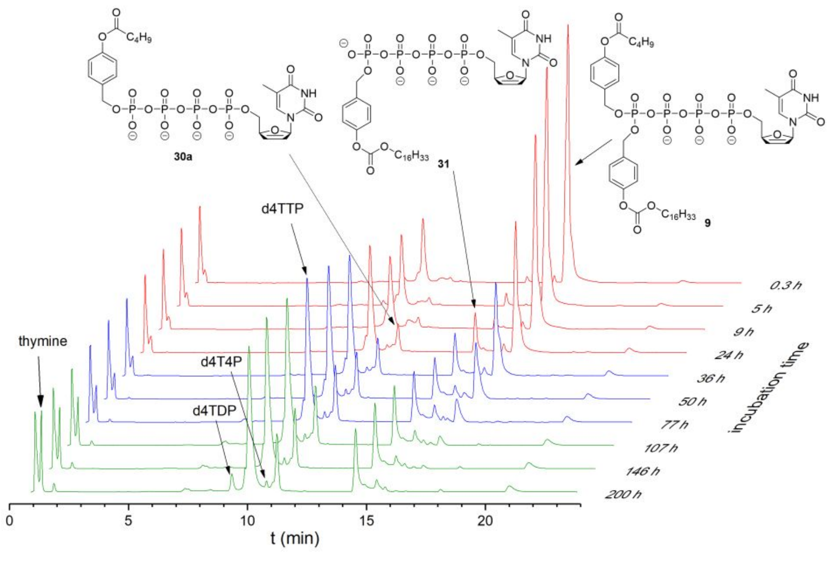

Figure S10. HPLC profiles of **9** after incubation in PBS (pH 7.3).

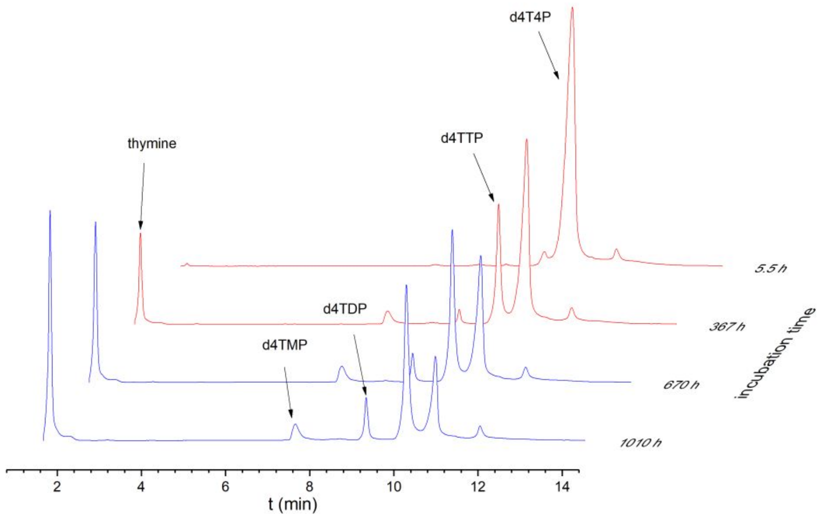

Figure S11. HPLC profiles of d4T4P after incubation in PBS (pH 7.3).

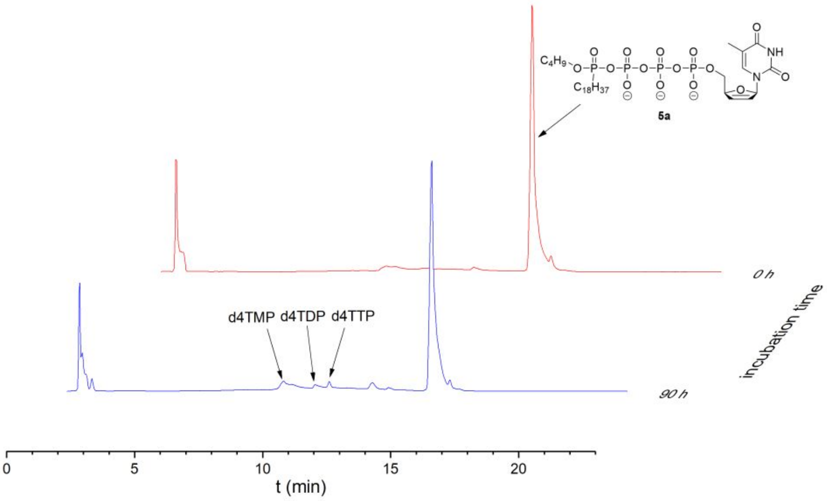

Figure S12. HPLC profiles of **5a** after incubation with PLE.

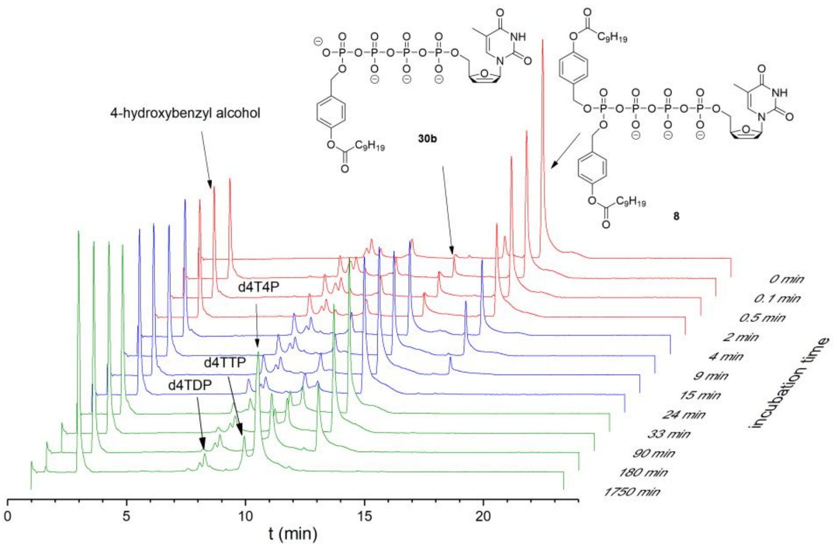

Figure S13. HPLC profiles of **8** after incubation with PLE.

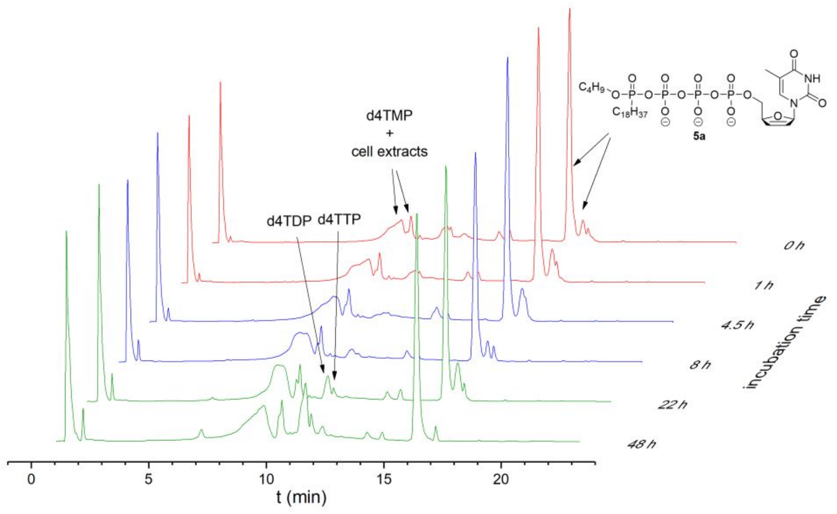

Figure S14. HPLC profiles of **5a** after incubation in CEM/0 cell extracts.

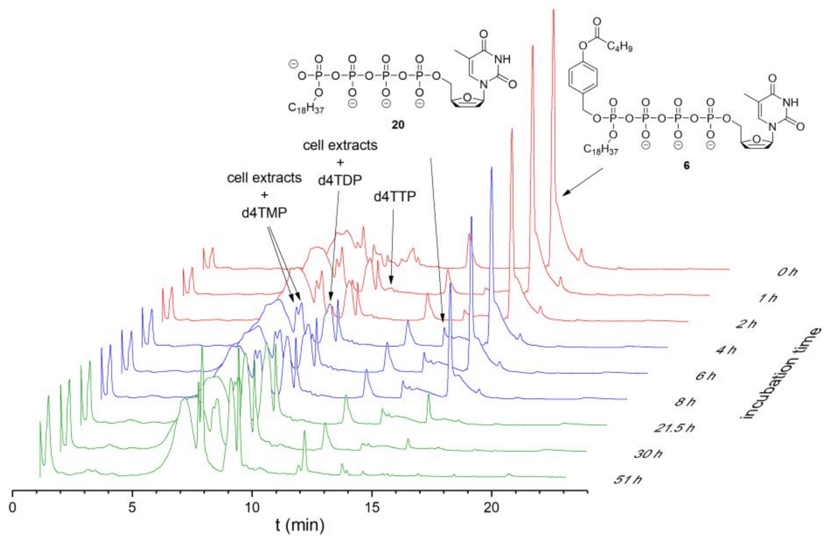

Figure S15. HPLC profiles of **6** after incubation in CEM/0 cell extracts.

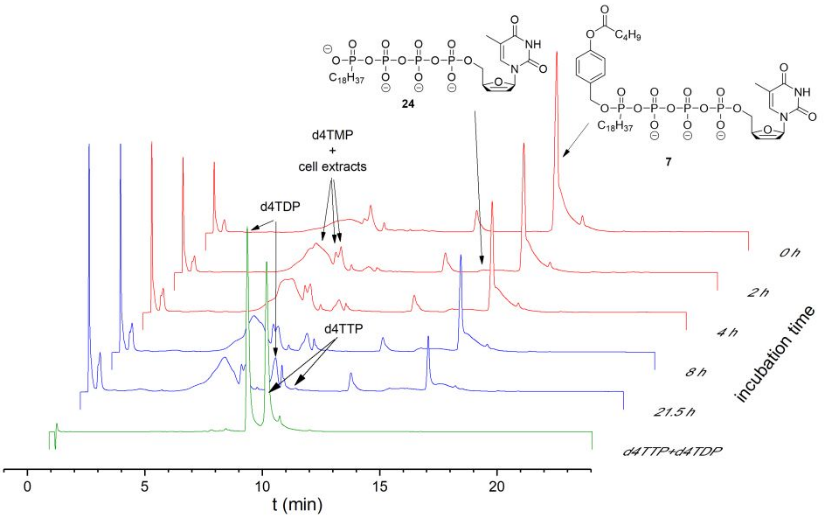

Figure S16. HPLC profiles of **7** after incubation in CEM/0 cell extracts.

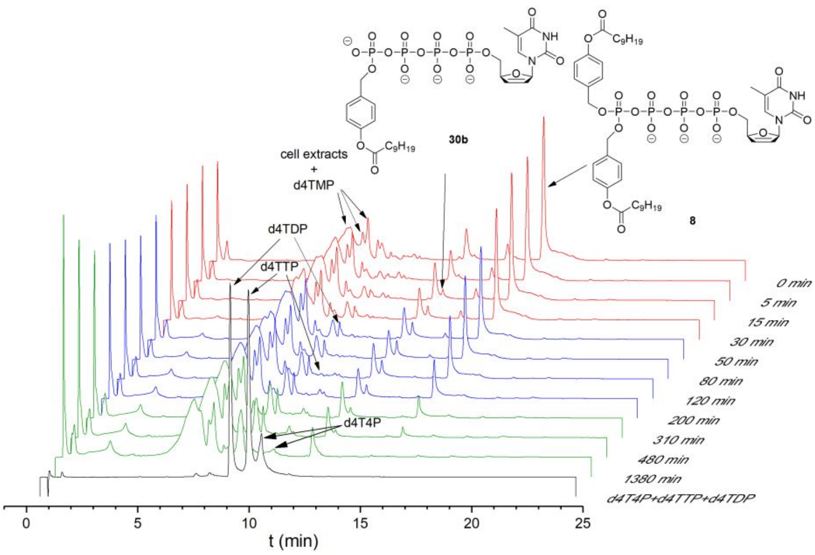

Figure S17. HPLC profiles of **8** after incubation in CEM/0 cell extracts.

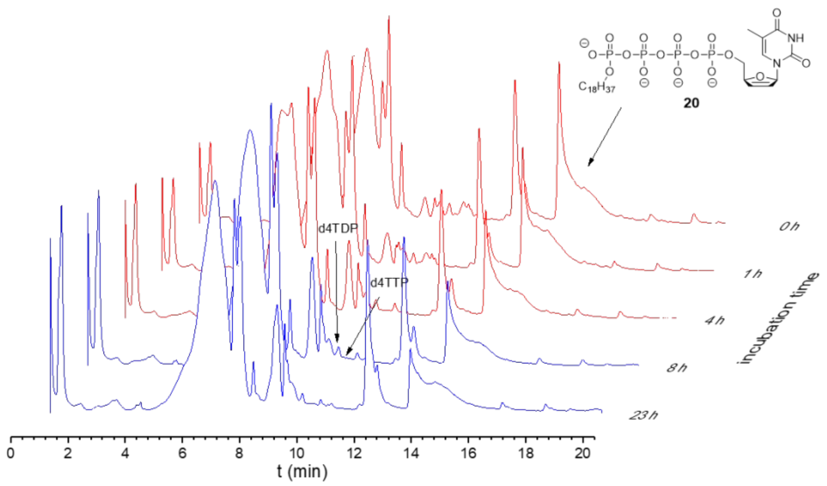

Figure S18. HPLC profiles of **20** after incubation in CEM/0 cell extracts.

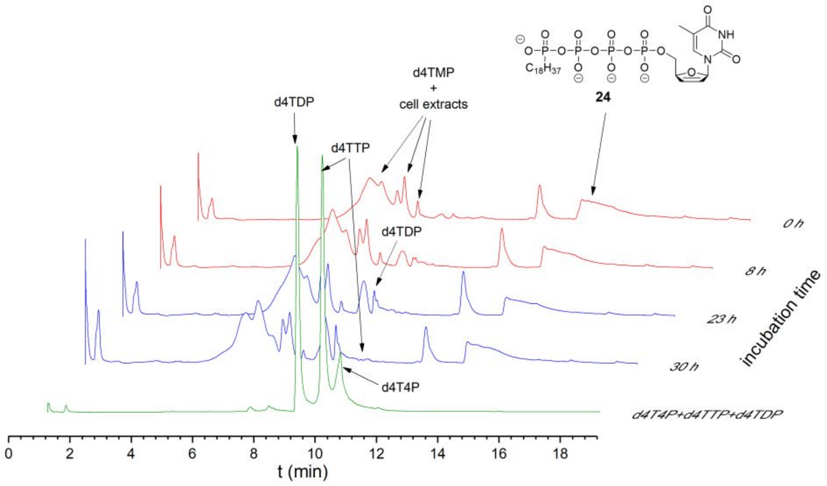

Figure S19. HPLC profiles of **24** after incubation in CEM/0 cell extracts.

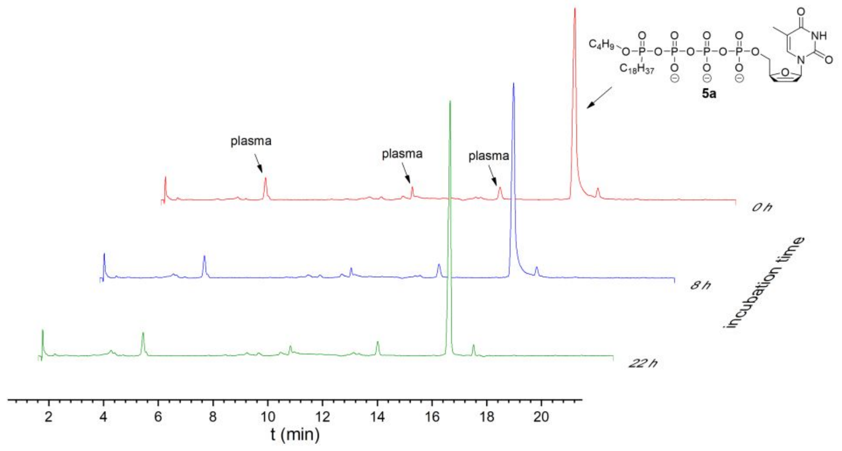

Figure S20. HPLC profiles of **5a** after incubation in citrate-stabilized human plasma.

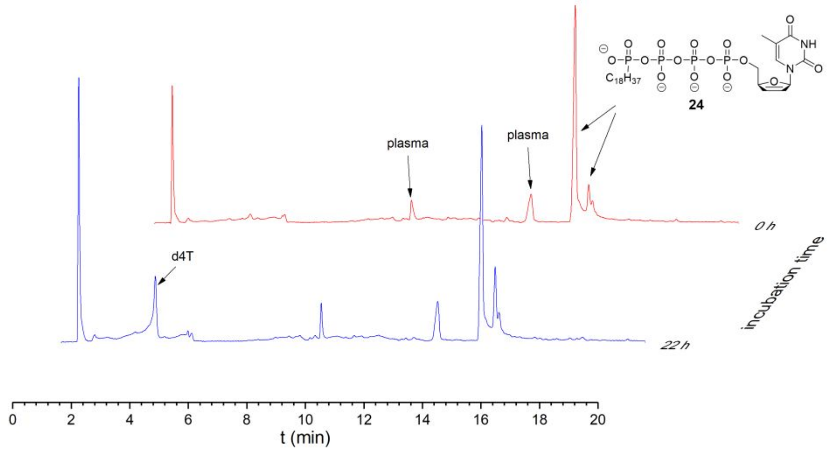

Figure S21. HPLC profiles of **24** after incubation in citrate-stabilized human plasma.

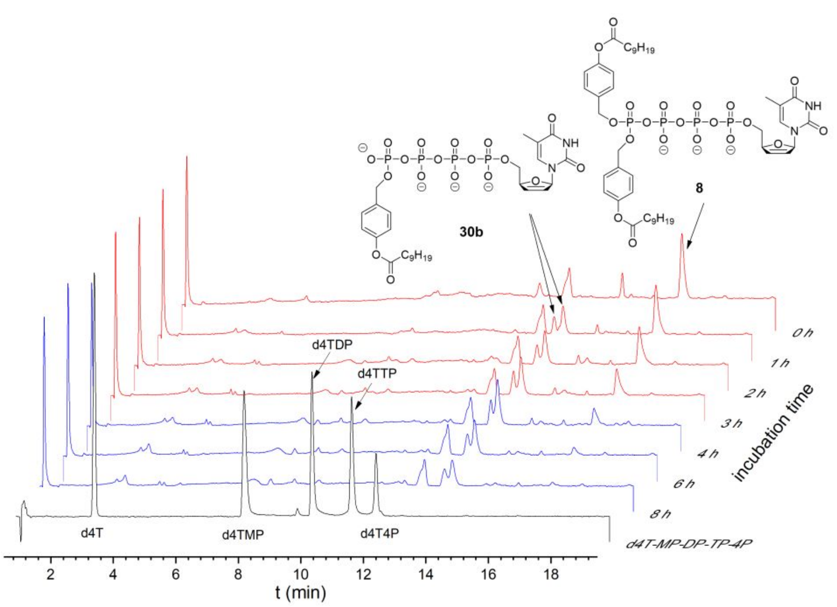

Figure S22. HPLC profiles of **6** after incubation in citrate-stabilized human plasma.

Spectral Data for New Compounds and HPLC Data.

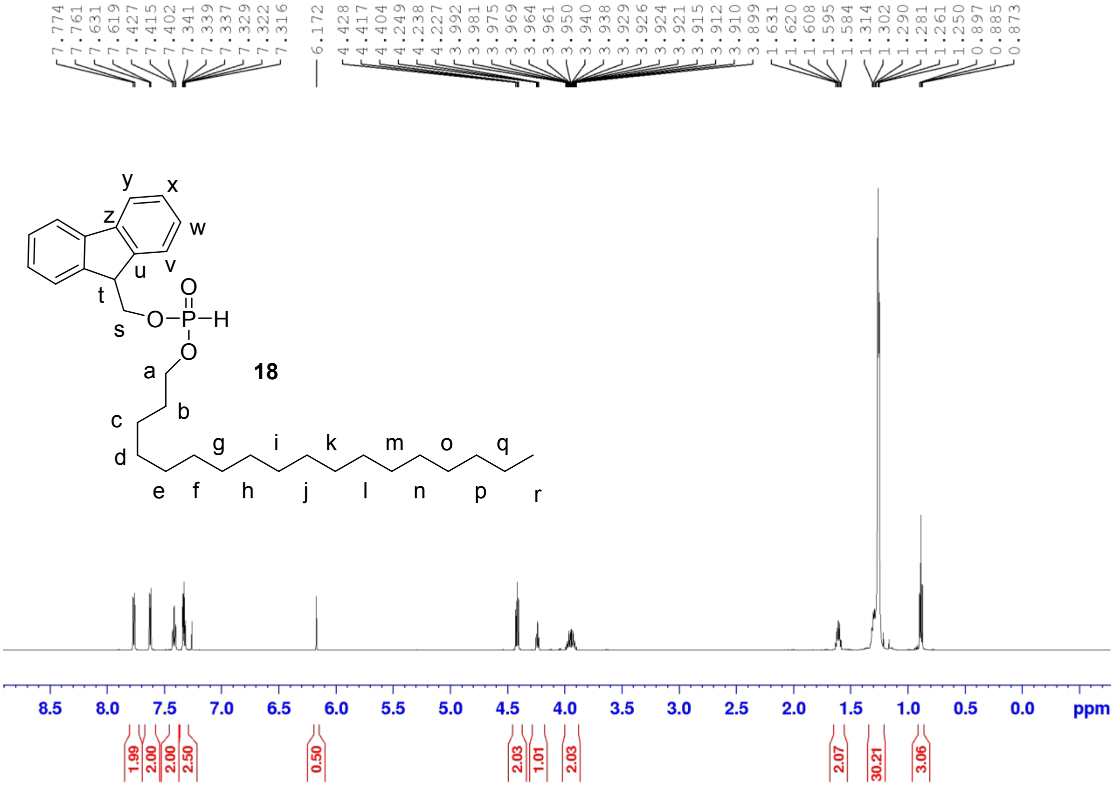

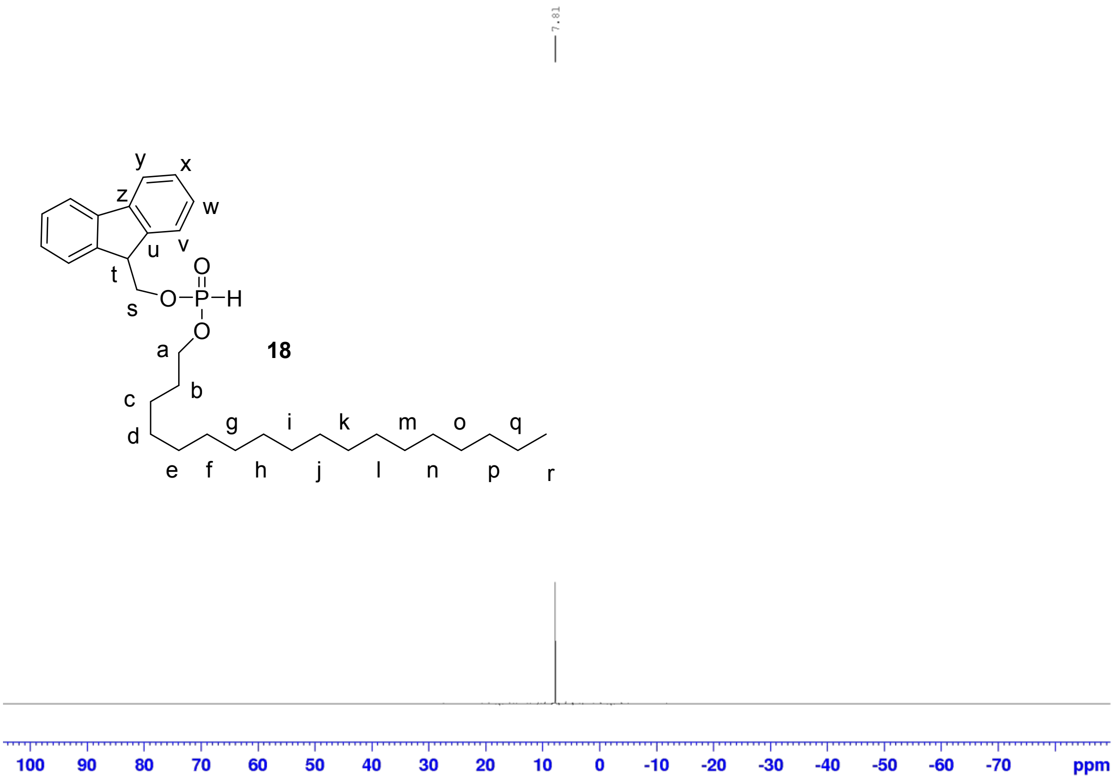

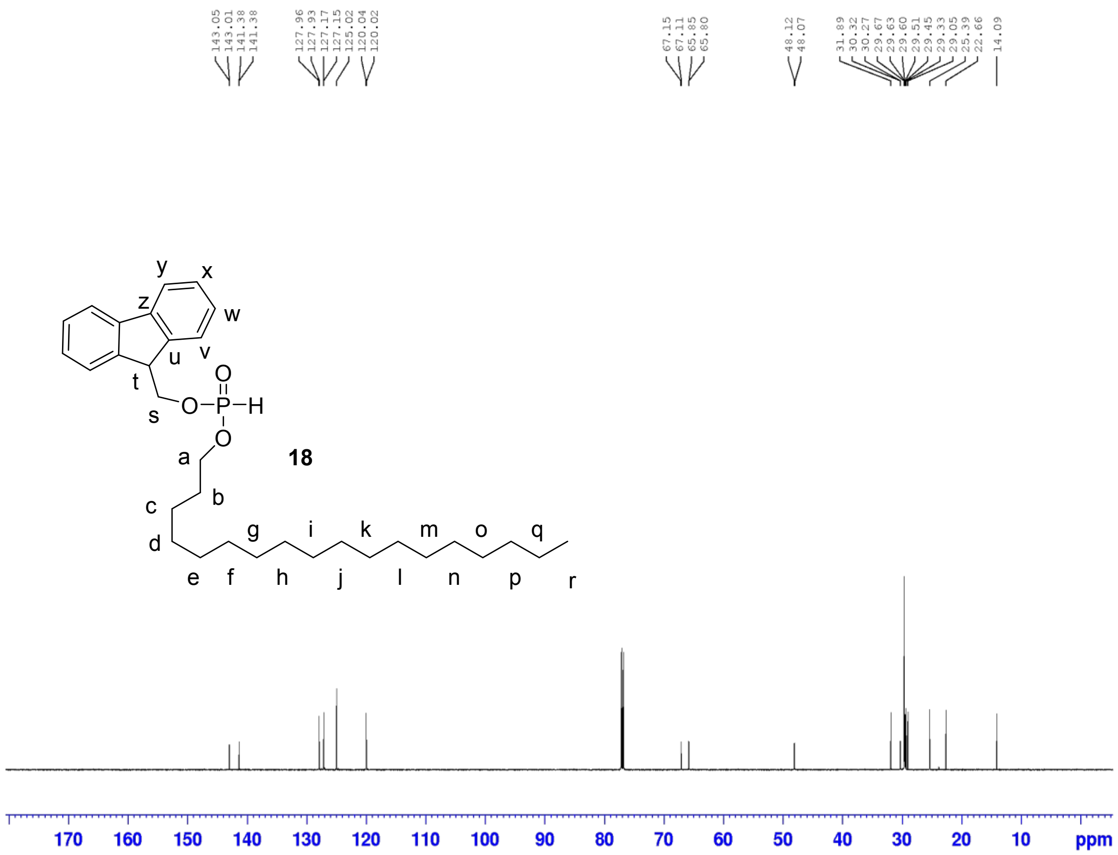
143.05
143.01
141.38
141.38
127.96
127.93
127.17
127.15
125.02
120.04
120.02
67.15
67.11
65.85
65.80
48.12
48.07
31.89
30.32
30.27
29.67
29.63
29.60
29.51
29.45
29.33
29.05
25.39
22.66
14.09
18
170 160 150 140 130 120 110 100 90 80 70 60 50 40 30 20 10 ppm

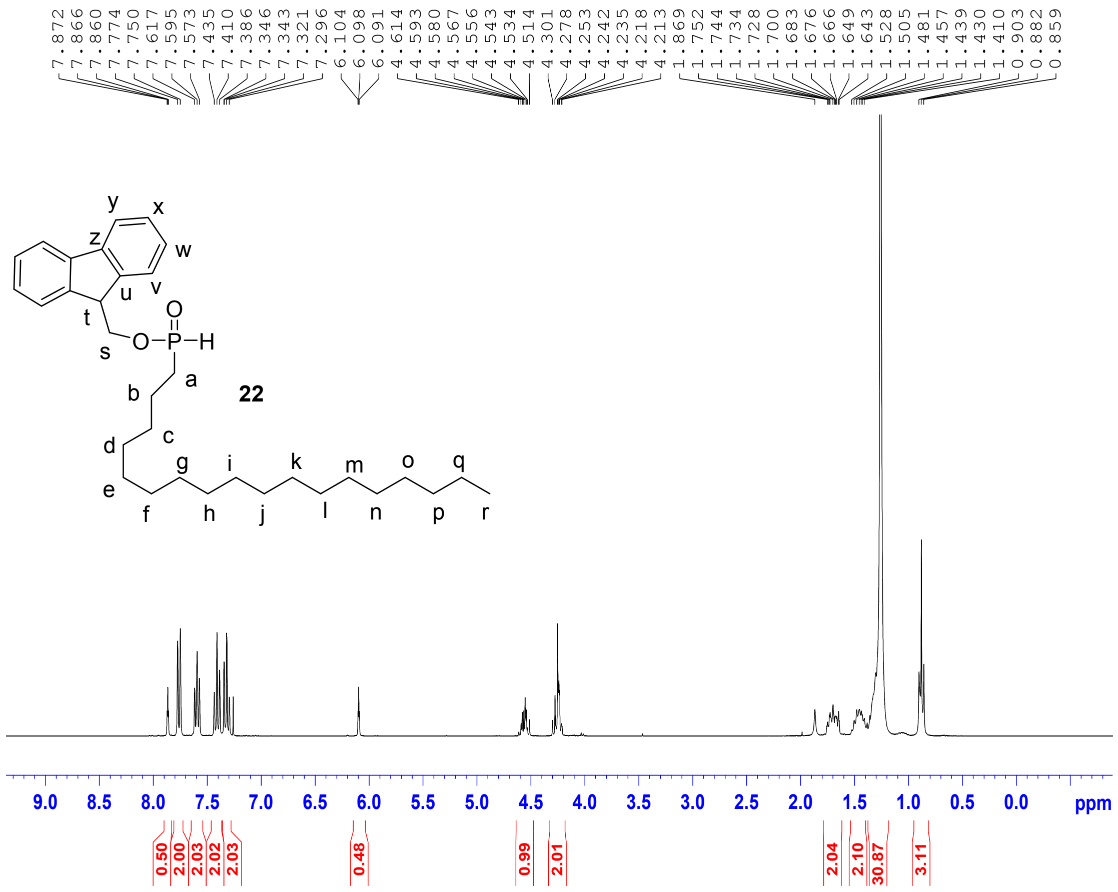
7.872
7.866
7.860
7.774
7.750
7.617
7.595
7.573
7.435
7.410
7.386
7.346
7.343
7.321
7.296
6.104
6.098
6.091
4.614
4.593
4.580
4.567
4.556
4.543
4.534
4.514
4.301
4.278
4.253
4.242
4.235
4.218
4.213
1.869
1.752
1.744
1.734
1.728
1.700
1.683
1.676
1.666
1.649
1.643
1.528
1.505
1.481
1.457
1.439
1.430
1.410
0.903
0.882
0.859
22
9.0 8.5 8.0 7.5 7.0 6.5 6.0 5.5 5.0 4.5 4.0 3.5 3.0 2.5 2.0 1.5 1.0 0.5 0.0 ppm
0.50
2.00
2.03
2.02
2.03
0.48
0.99
2.01
2.04
2.10
30.87
3.11

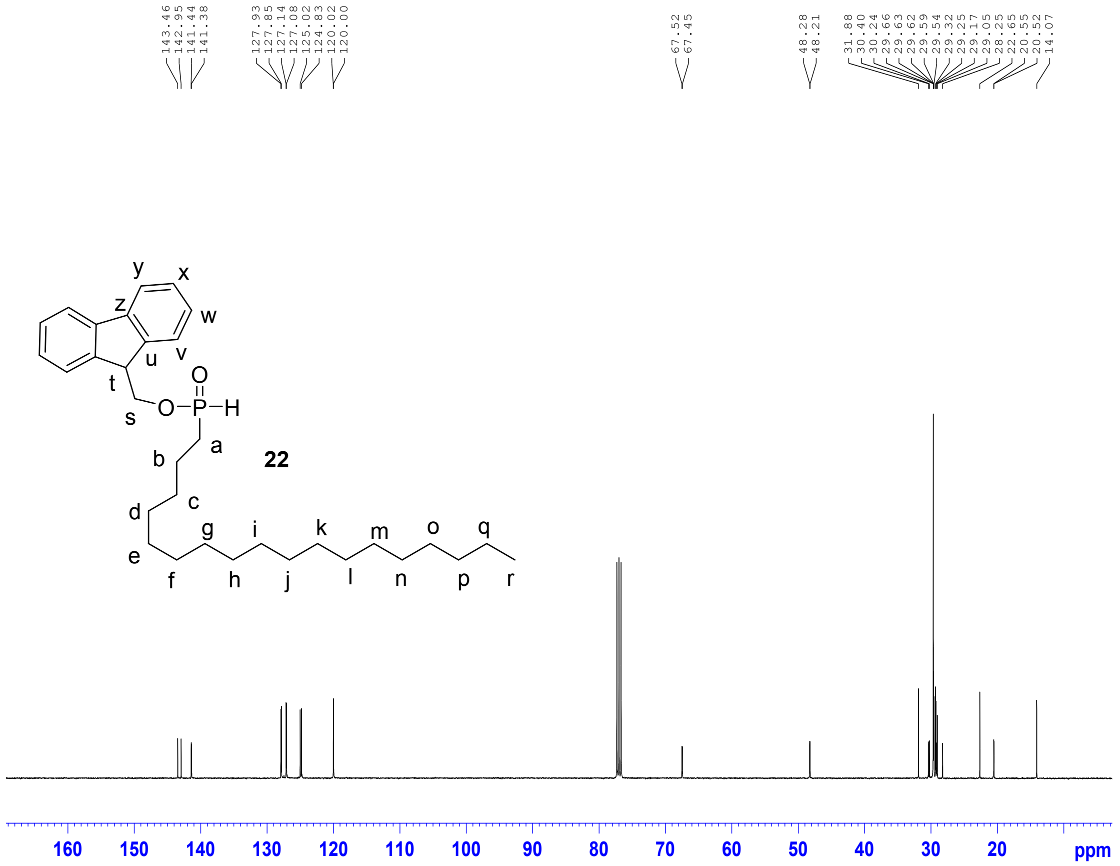
143.46
142.95
141.44
141.38
127.93
127.85
127.14
127.08
125.02
124.83
120.02
120.00
67.52
67.45
48.28
48.21
31.88
30.40
30.24
29.66
29.63
29.62
29.59
29.54
29.32
29.25
29.17
29.05
28.25
22.65
20.55
20.52
14.07
22
160 150 140 130 120 110 100 90 80 70 60 50 40 30 20 ppm

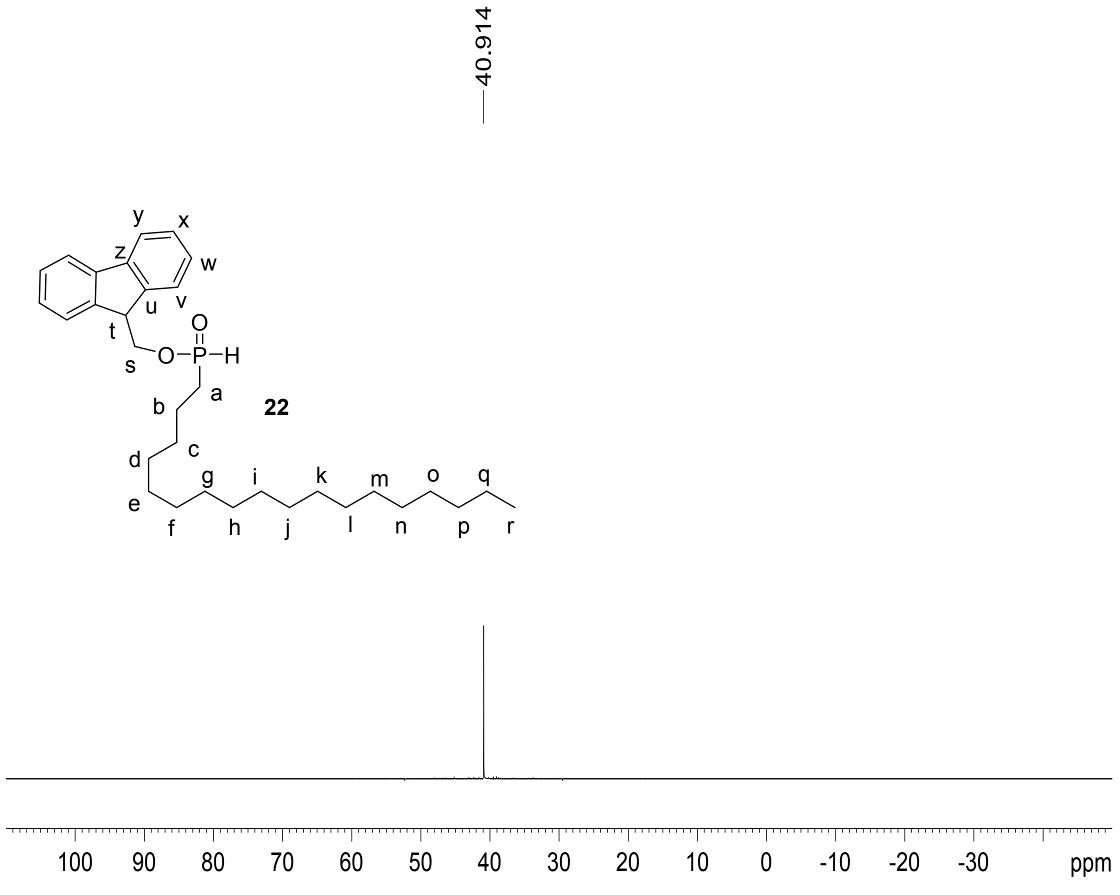
40.914
22
100 90 80 70 60 50 40 30 20 10 0 -10 -20 -30 ppm

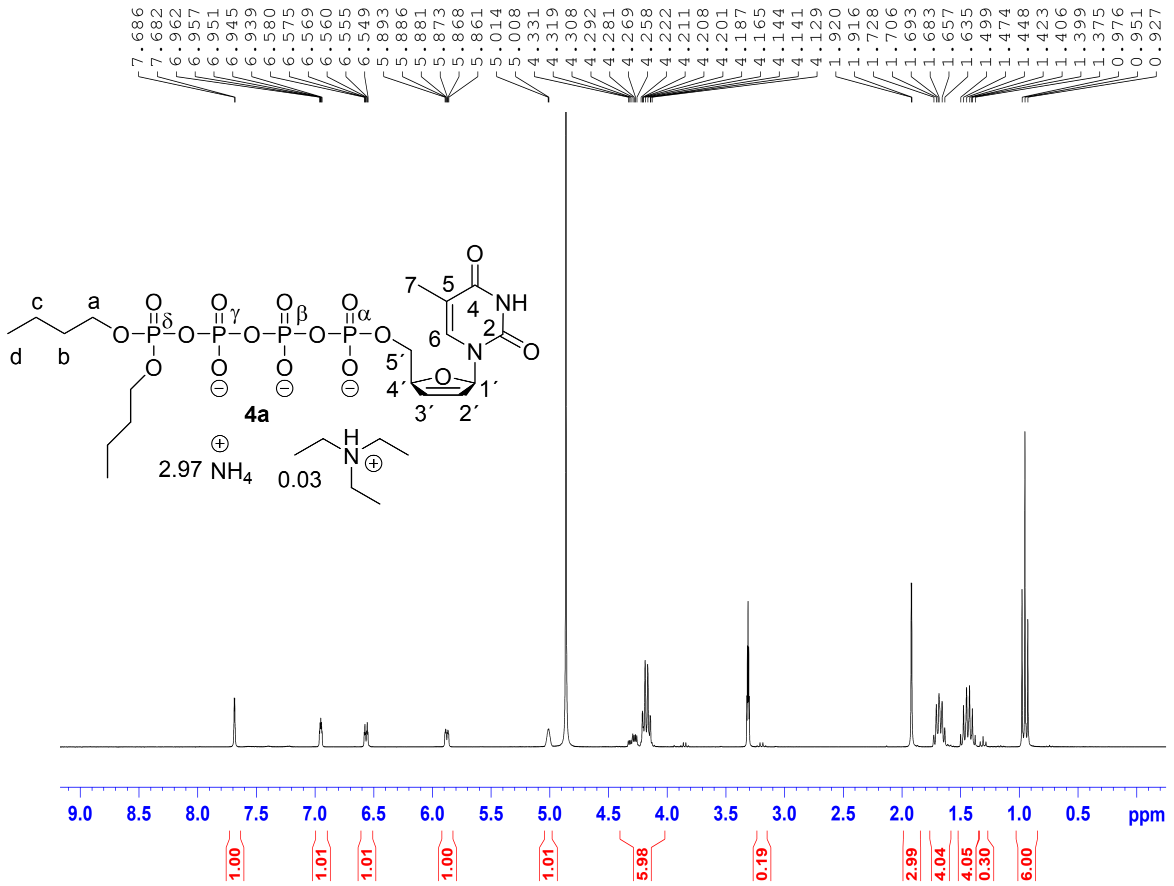
4a
2.97 NH4
0.03
ppm

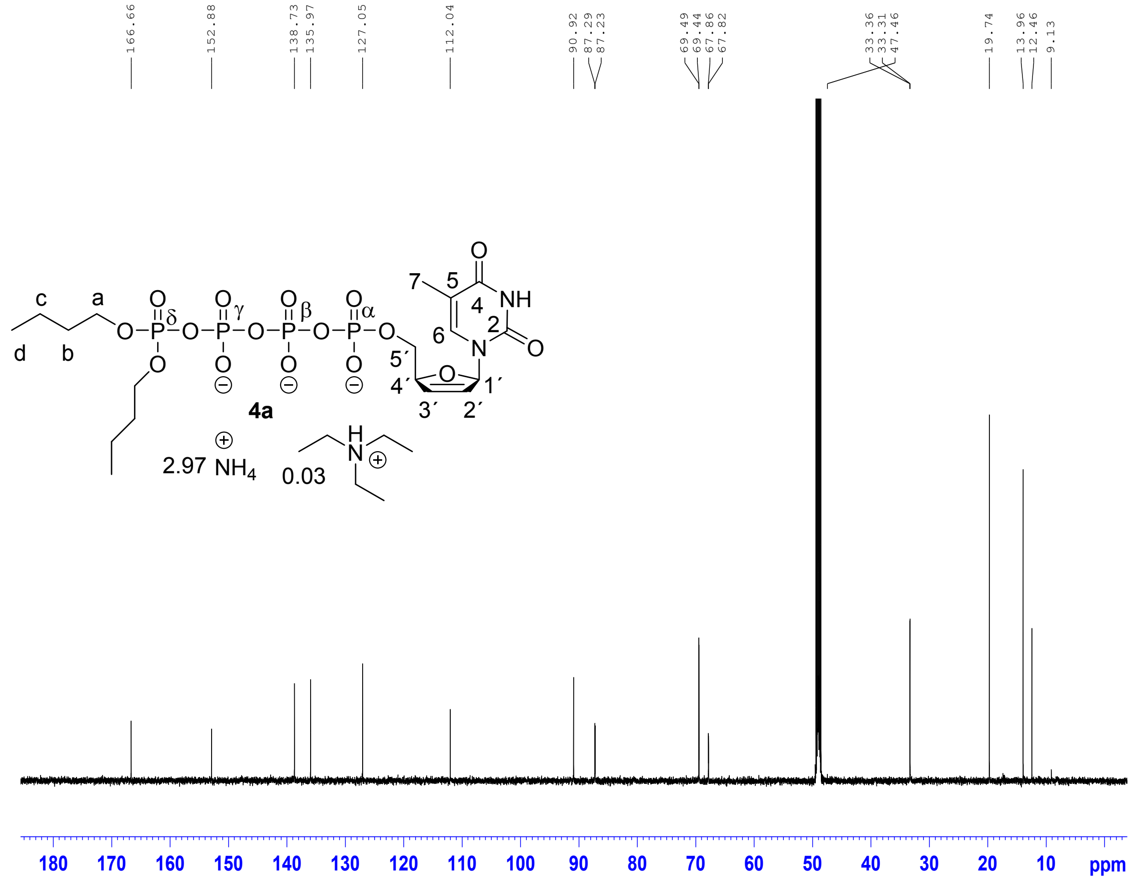
4a
2.97 NH4
0.03
ppm

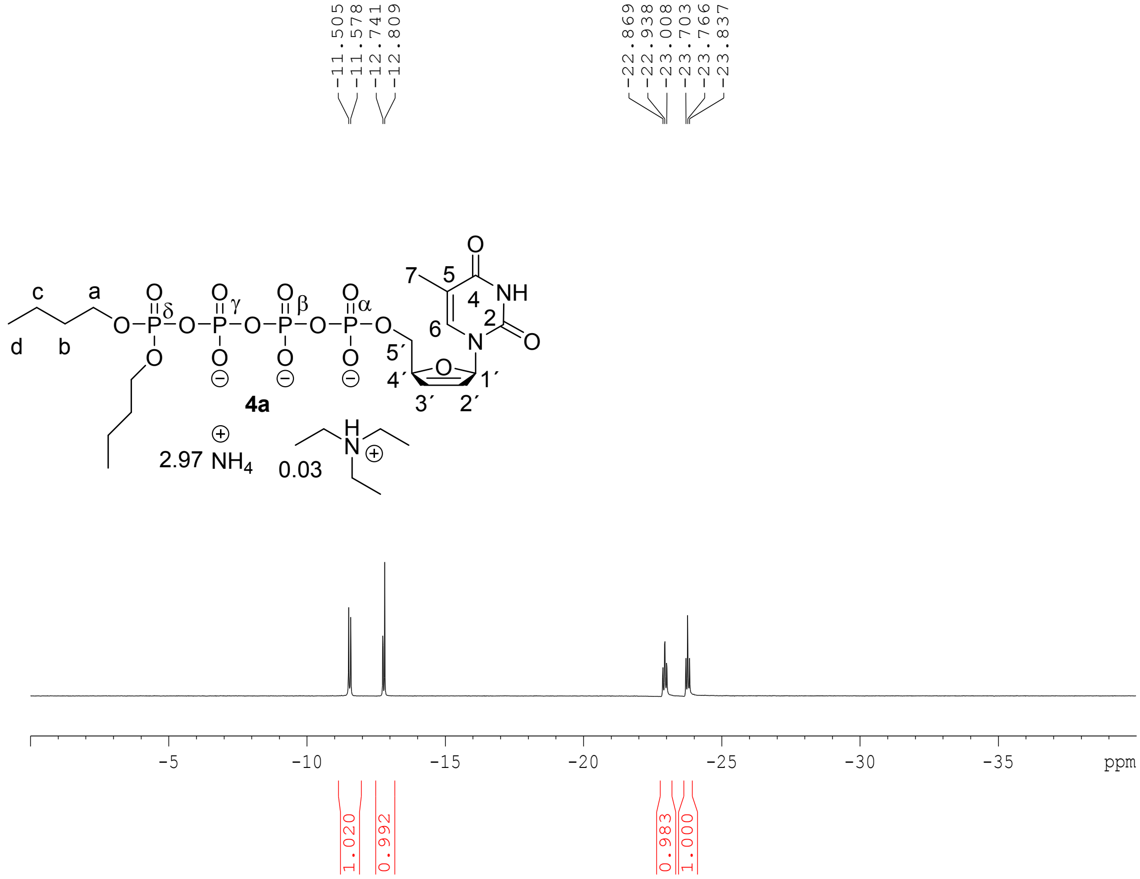
-11.505
-11.578
-12.741
-12.809
-22.869
-22.938
-23.008
-23.703
-23.766
-23.837
4a
2.97 NH4
0.03
-5
-10
-15
-20
-25
-30
-35
ppm
1.020
0.992
0.983
1.000

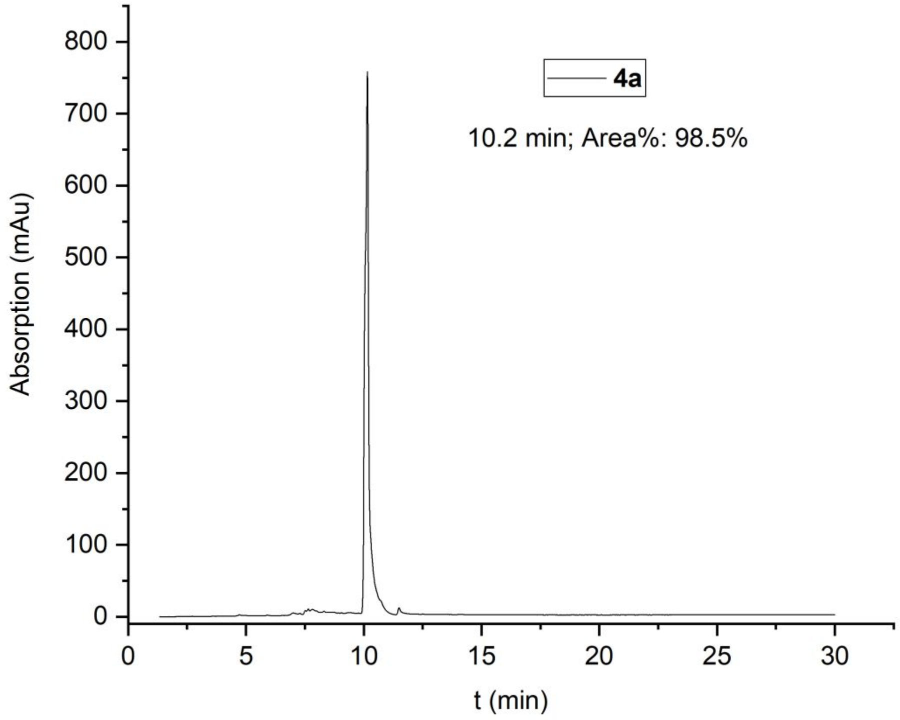
4a
10.2 min; Area%: 98.5%
Absorption (mAu)
t (min)

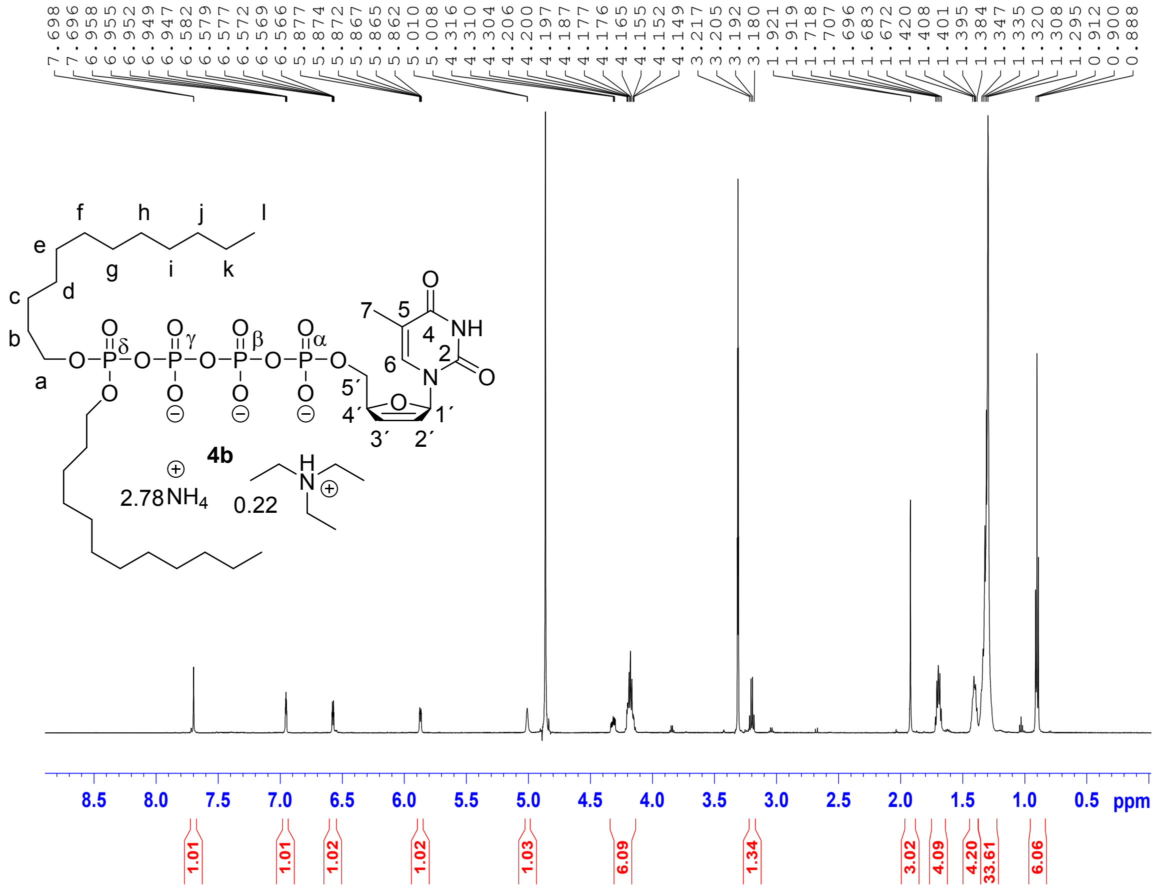

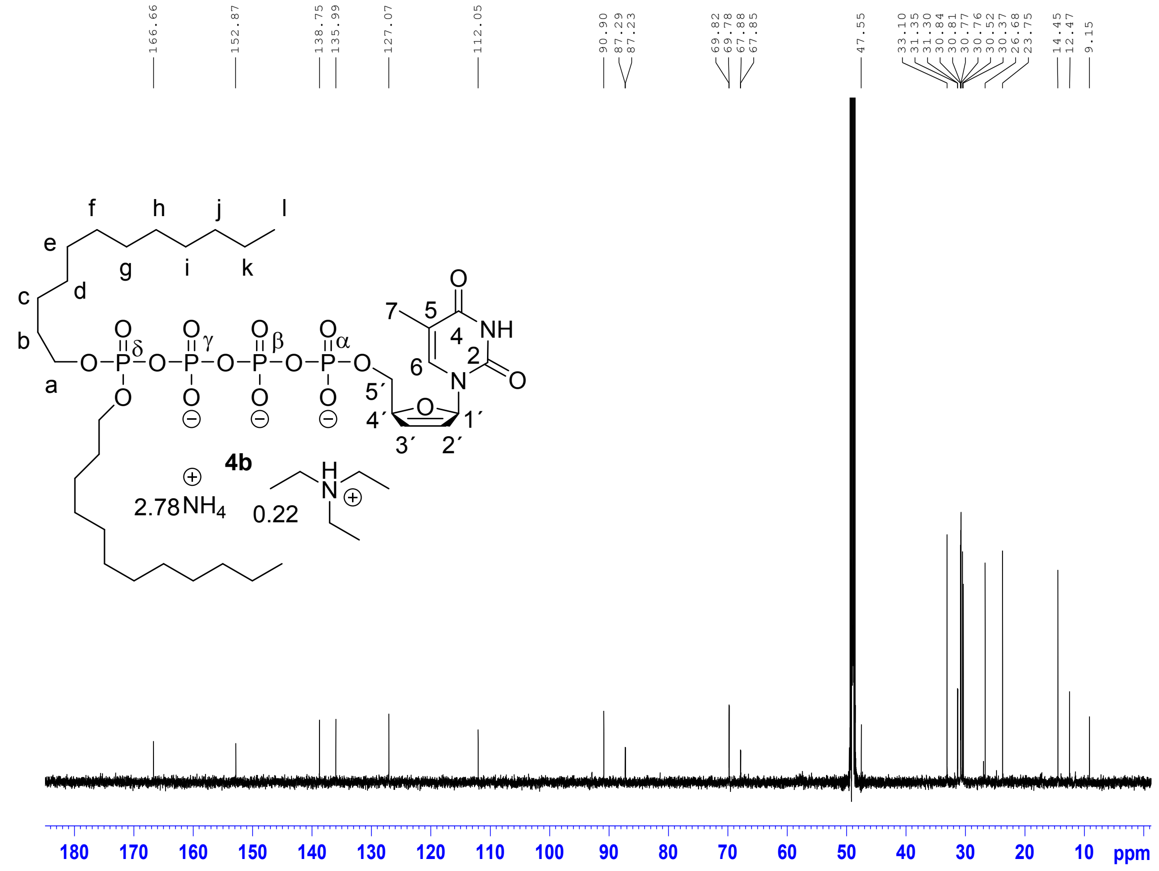

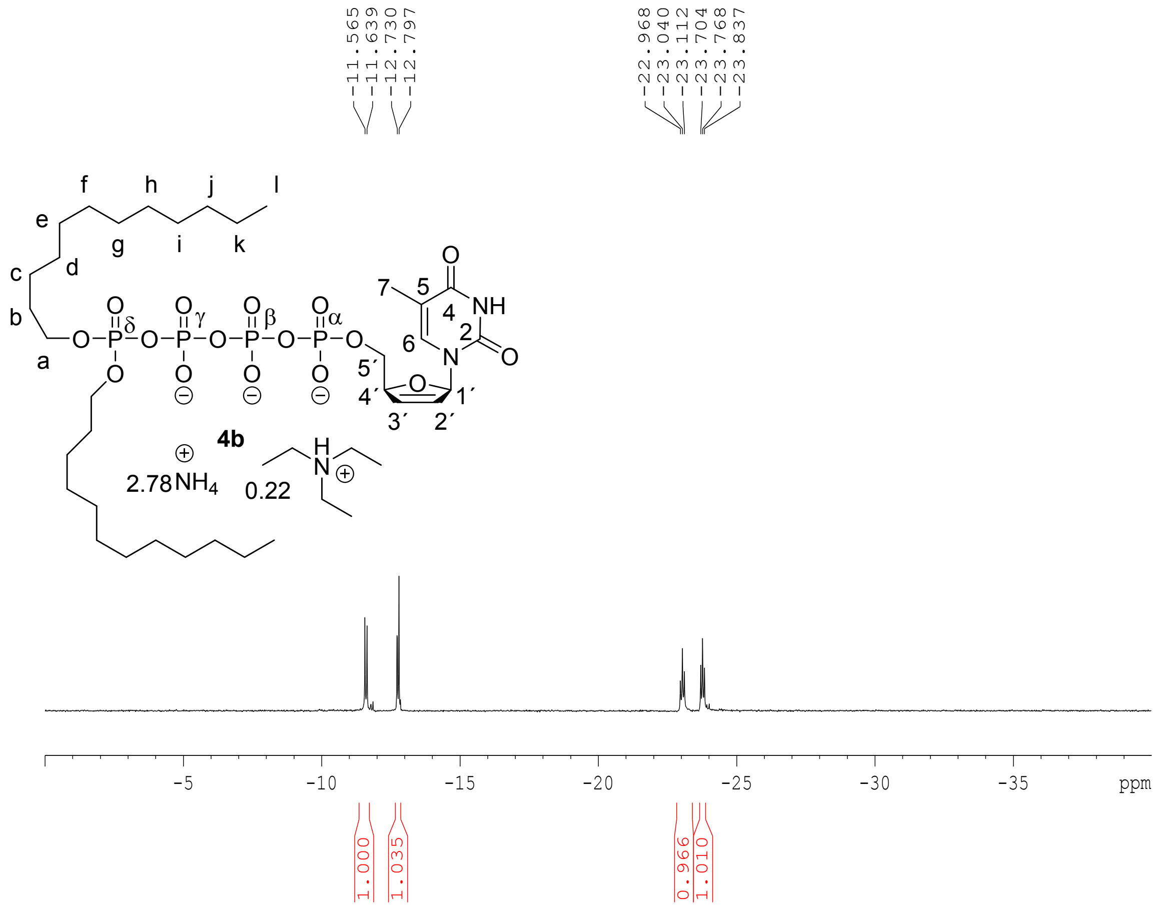

-11.565
-11.639
-12.730
-12.797
-22.968
-23.040
-23.112
-23.704
-23.768
-23.837
4b
2.78 NH4
0.22
-5
-10
-15
-20
-25
-30
-35
ppm
1.000
1.035
0.966
1.010

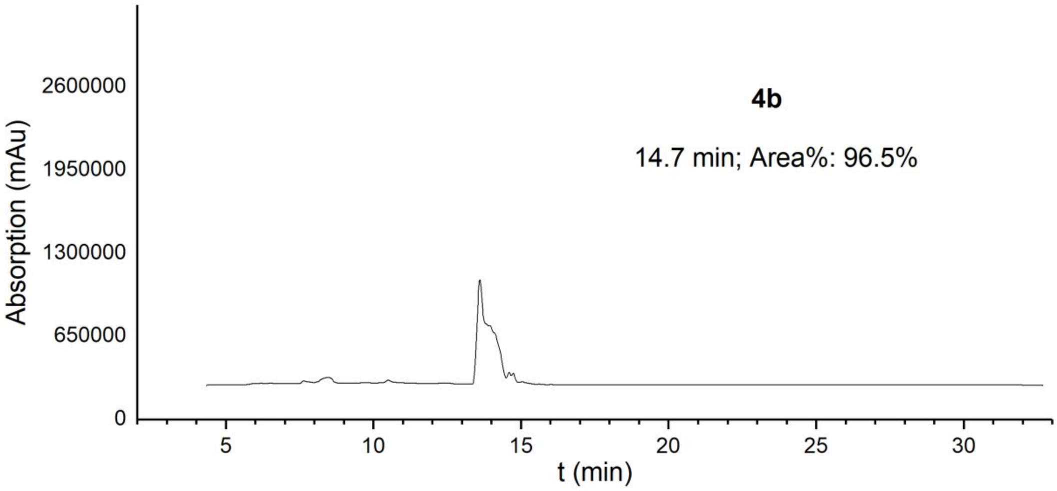

4b
14.7 min; Area%: 96.5%
Absorption (mAu)
2600000
1950000
1300000
650000
0
5
10
15
20
25
30
t (min)

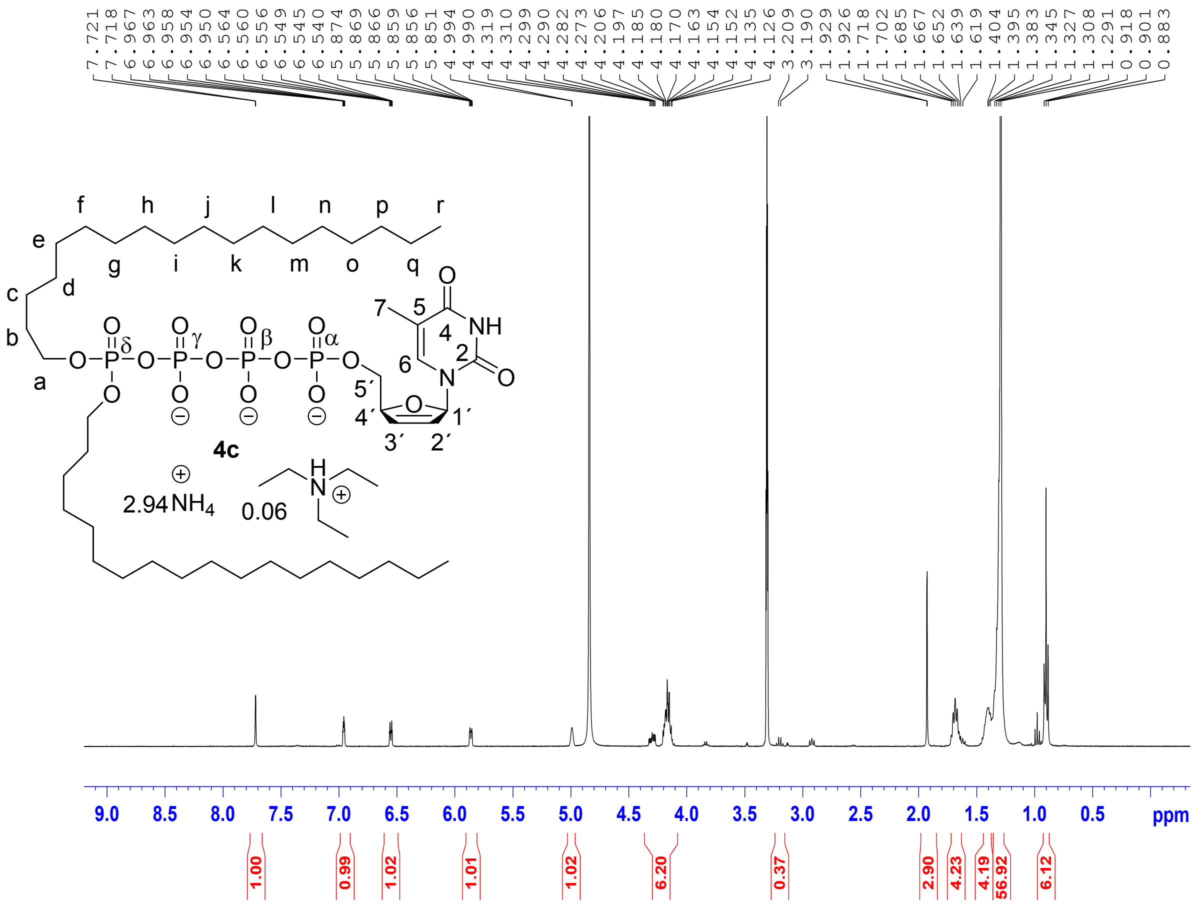
7.721 7.718 6.967 6.963 6.958 6.954 6.950 6.564 6.560 6.556 6.549 6.545 6.540 5.874 5.869 5.866 5.859 5.856 5.851 4.994 4.990 4.319 4.310 4.299 4.290 4.282 4.273 4.206 4.197 4.185 4.180 4.170 4.163 4.154 4.152 4.135 4.126 3.209 3.190 1.929 1.926 1.718 1.702 1.685 1.667 1.652 1.639 1.619 1.404 1.395 1.383 1.345 1.327 1.308 1.291 0.918 0.901 0.883
4c
2.94 NH4
0.06
ppm
1.00 0.99 1.02 1.01 1.02 6.20 0.37 2.90 4.23 4.19 56.92 6.12

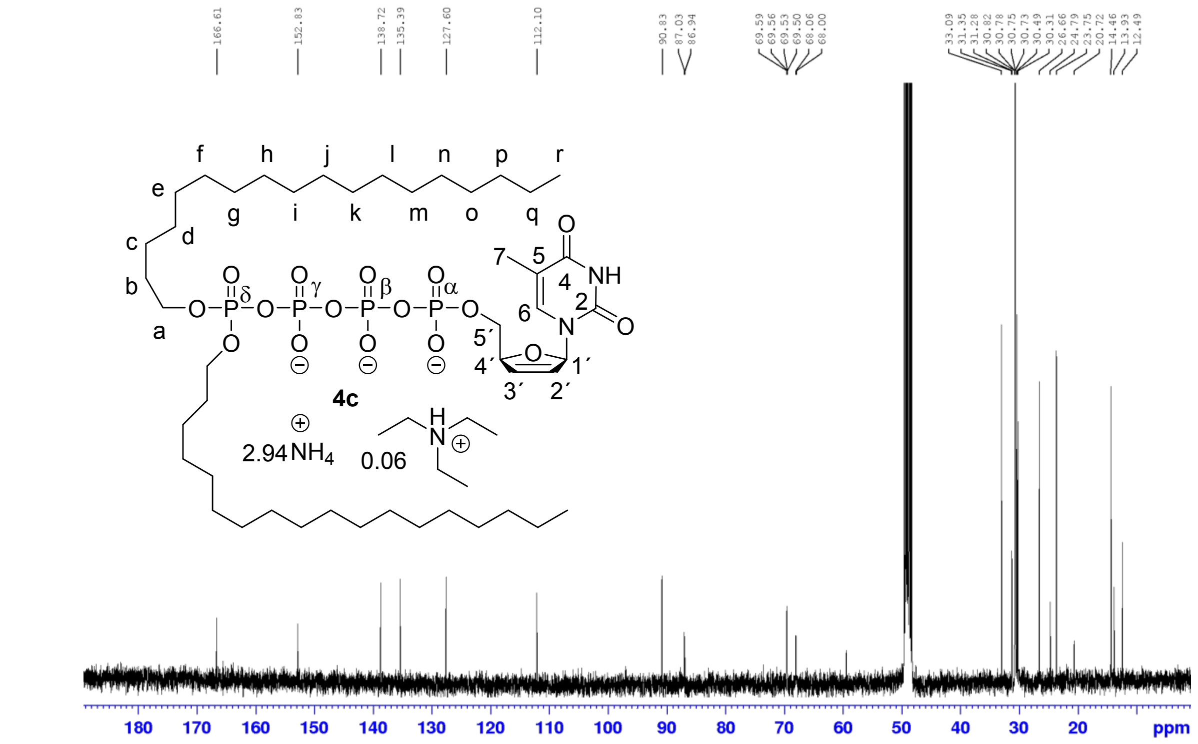
166.61 152.83 138.72 135.39 127.60 112.10 90.83 87.03 86.94 69.59 69.56 69.53 69.50 68.06 68.00 33.09 31.35 31.28 30.82 30.78 30.75 30.73 30.49 30.31 26.66 24.79 23.75 20.72 14.46 13.93 12.49
4c
2.94 NH4
0.06
ppm

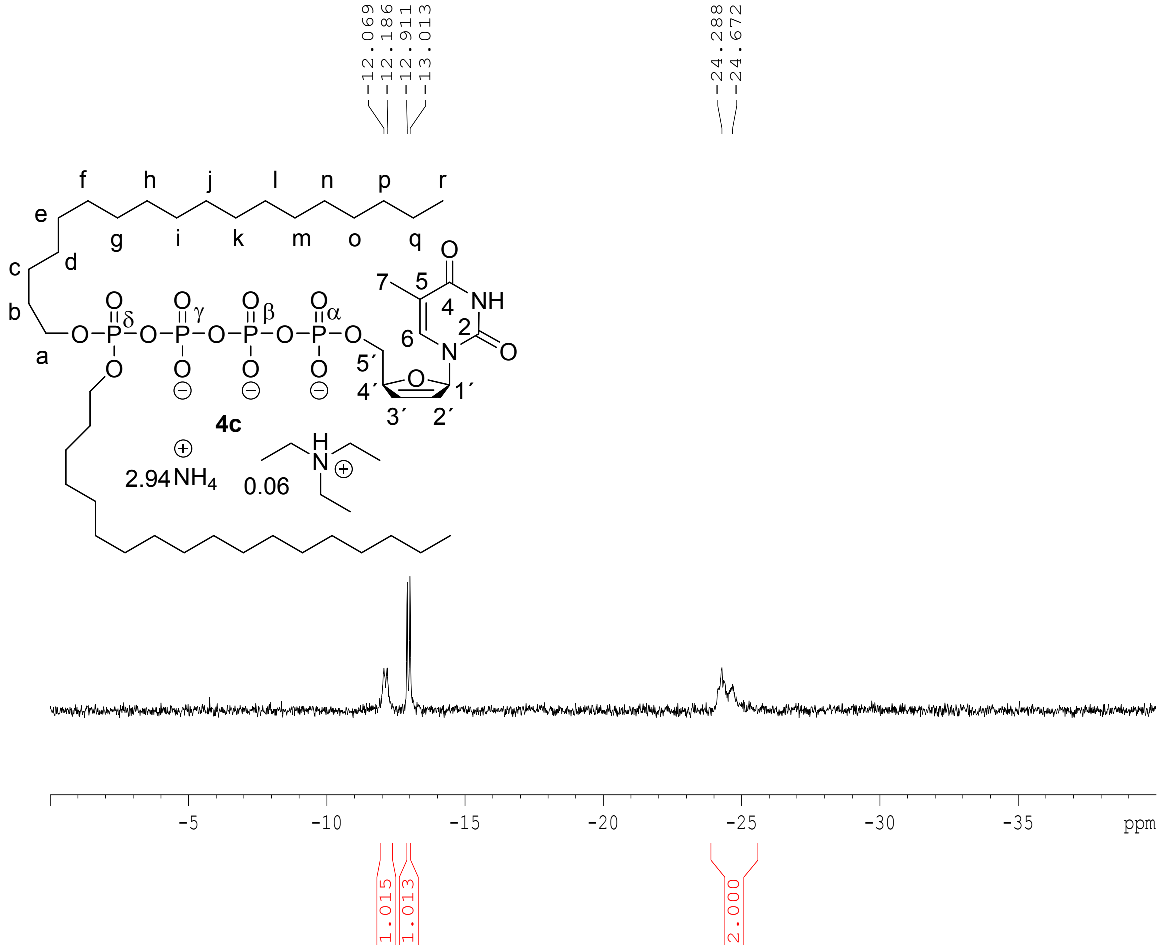
-12.069
-12.186
-12.911
-13.013
-24.288
-24.672
f h j l n p r
e g i k m o q
c d
b a
7 5 4 NH 2 6 N O
δ γ β α
5´ 4´ 3´ 2´ 1´
4c
2.94 NH4
0.06
-5 -10 -15 -20 -25 -30 -35 ppm
1.015
1.013
2.000

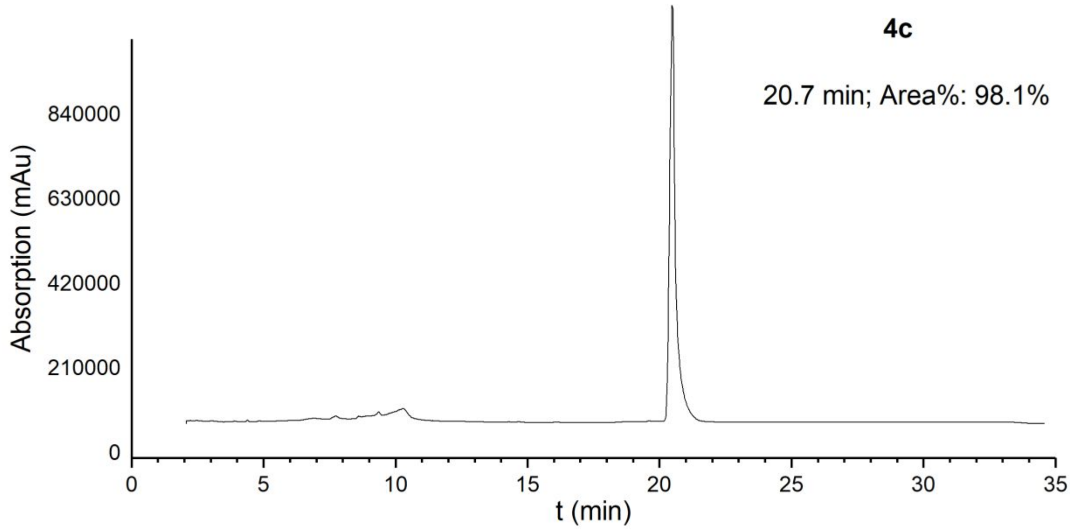
4c
20.7 min; Area%: 98.1%
Absorption (mAu)
840000
630000
420000
210000
0
0 5 10 15 20 25 30 35
t (min)

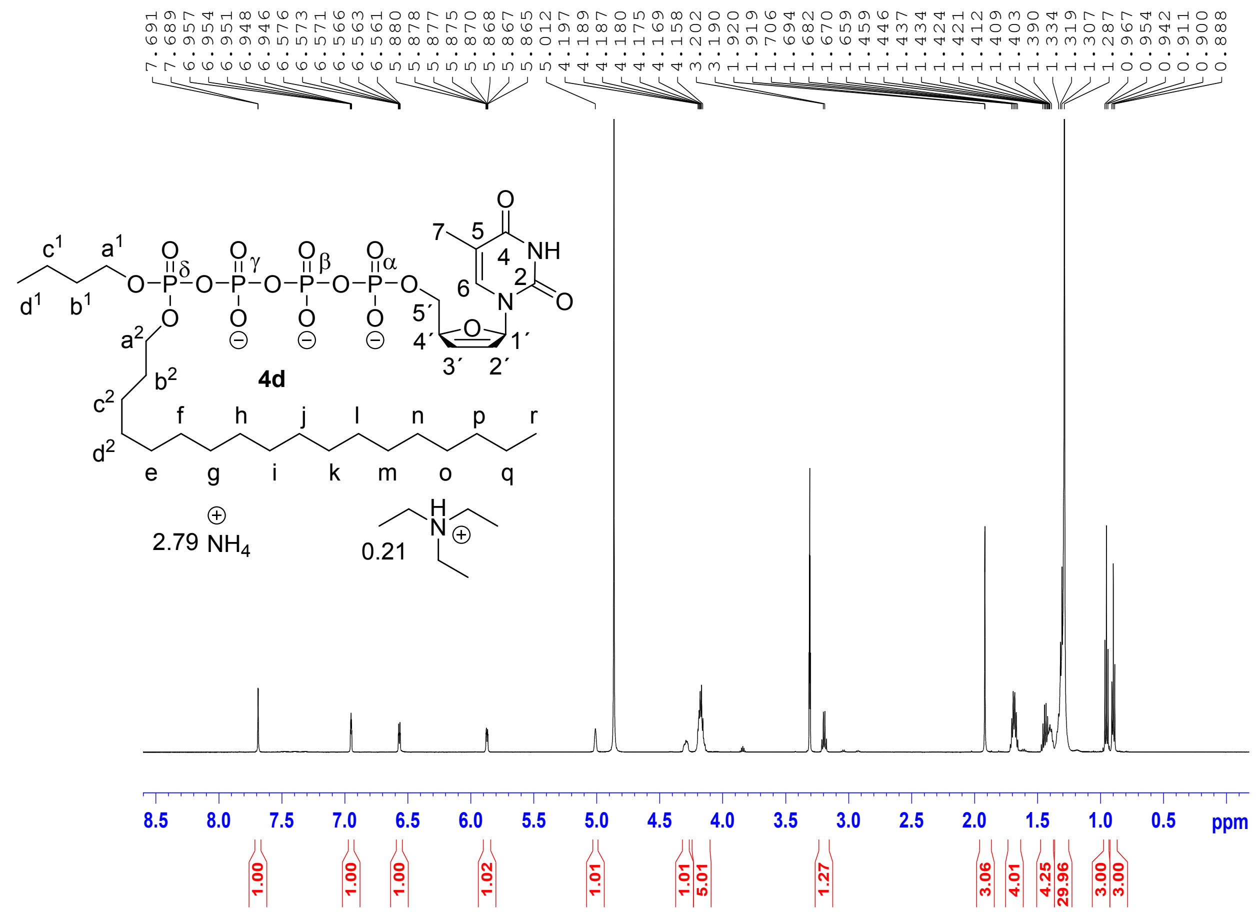
7.691 7.689 6.957 6.954 6.951 6.948 6.946 6.576 6.573 6.571 6.566 6.563 6.561 5.880 5.878 5.877 5.875 5.870 5.868 5.867 5.865 5.012 4.197 4.189 4.187 4.180 4.175 4.169 4.158 3.202 3.190 1.920 1.919 1.706 1.694 1.682 1.670 1.659 1.459 1.446 1.437 1.434 1.424 1.421 1.412 1.409 1.403 1.390 1.334 1.319 1.307 1.287 0.967 0.954 0.942 0.911 0.900 0.888
4d
2.79 NH4
0.21
8.5 8.0 7.5 7.0 6.5 6.0 5.5 5.0 4.5 4.0 3.5 3.0 2.5 2.0 1.5 1.0 0.5 ppm
1.00 1.00 1.00 1.02 1.01 1.01 5.01 1.27 3.06 4.01 4.25 29.96 3.00 3.00

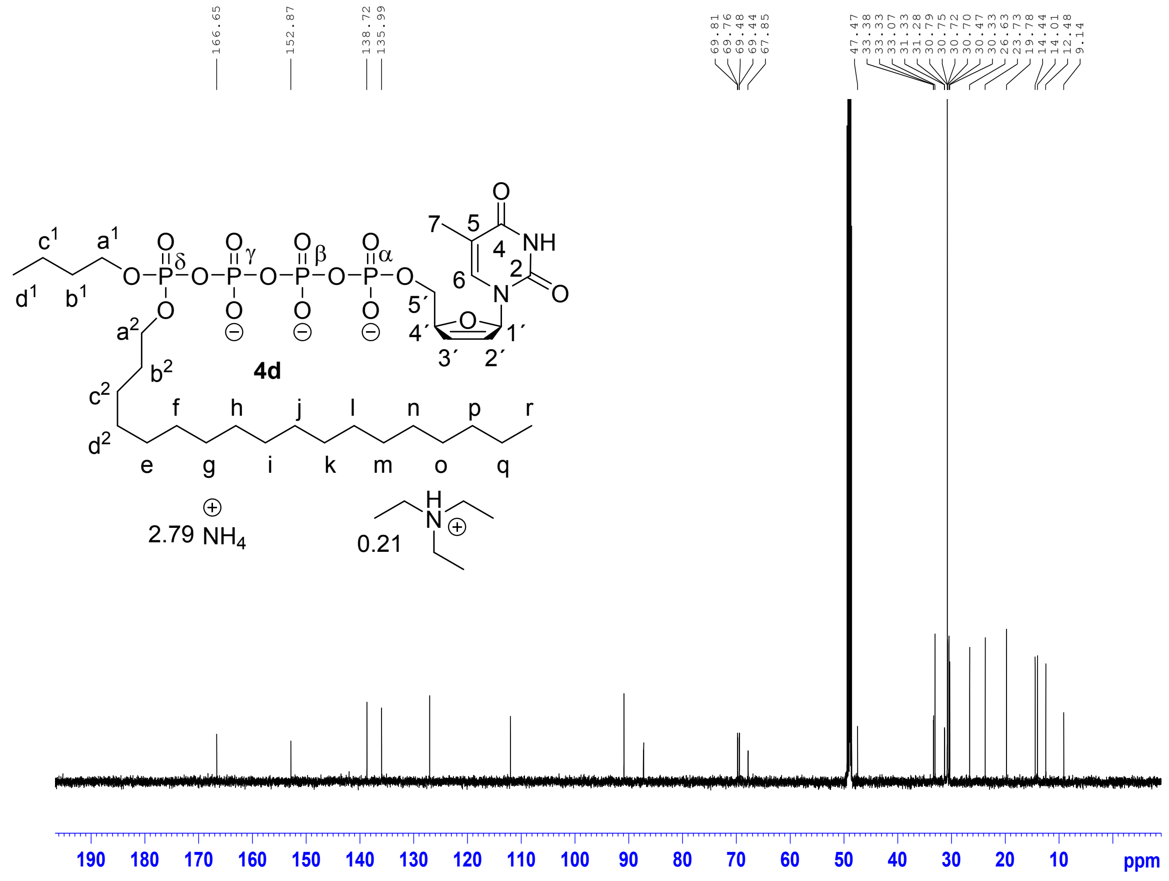
166.65 152.87 138.72 135.99 69.81 69.76 69.48 69.44 67.85 47.47 33.38 33.33 33.07 31.33 31.28 30.79 30.75 30.72 30.70 30.47 30.33 26.63 23.73 19.78 14.44 14.01 12.48 9.14
4d
2.79 NH4
0.21
190 180 170 160 150 140 130 120 110 100 90 80 70 60 50 40 30 20 10 ppm

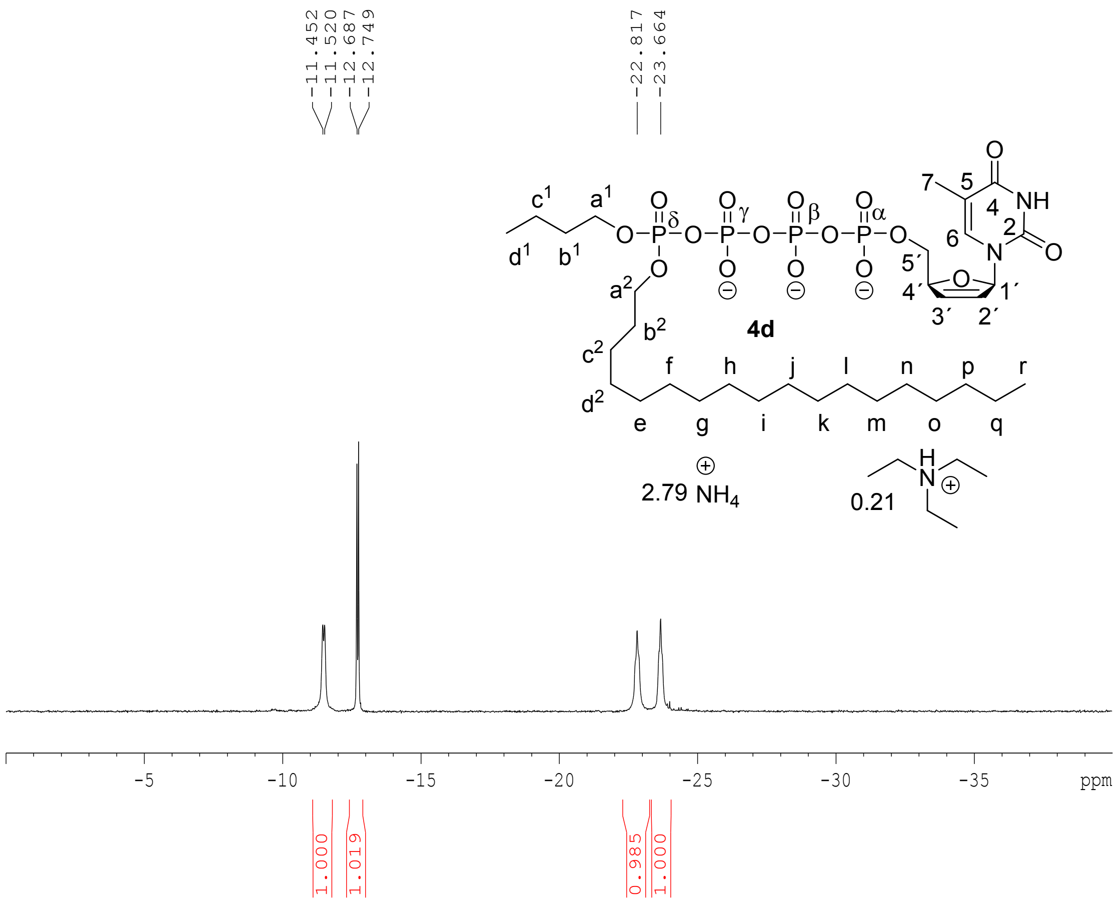
-11.452
-11.520
-12.687
-12.749
-22.817
-23.664
4d
2.79 NH4
0.21
ppm
1.000
1.019
0.985
1.000

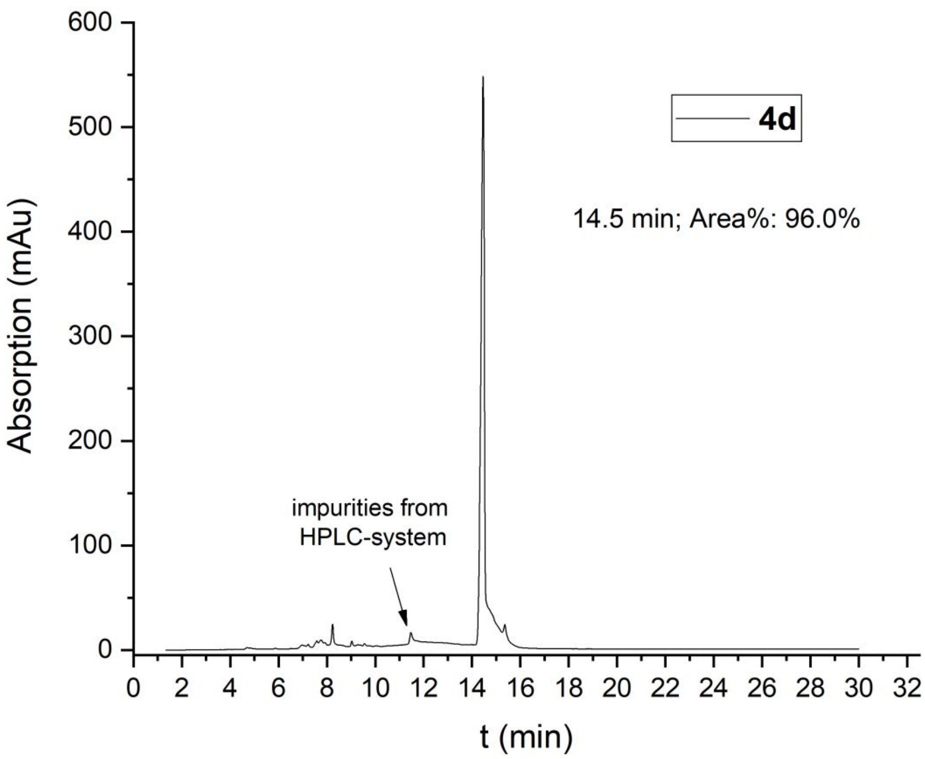
4d
14.5 min; Area%: 96.0%
impurities from
HPLC-system
Absorption (mAu)
t (min)

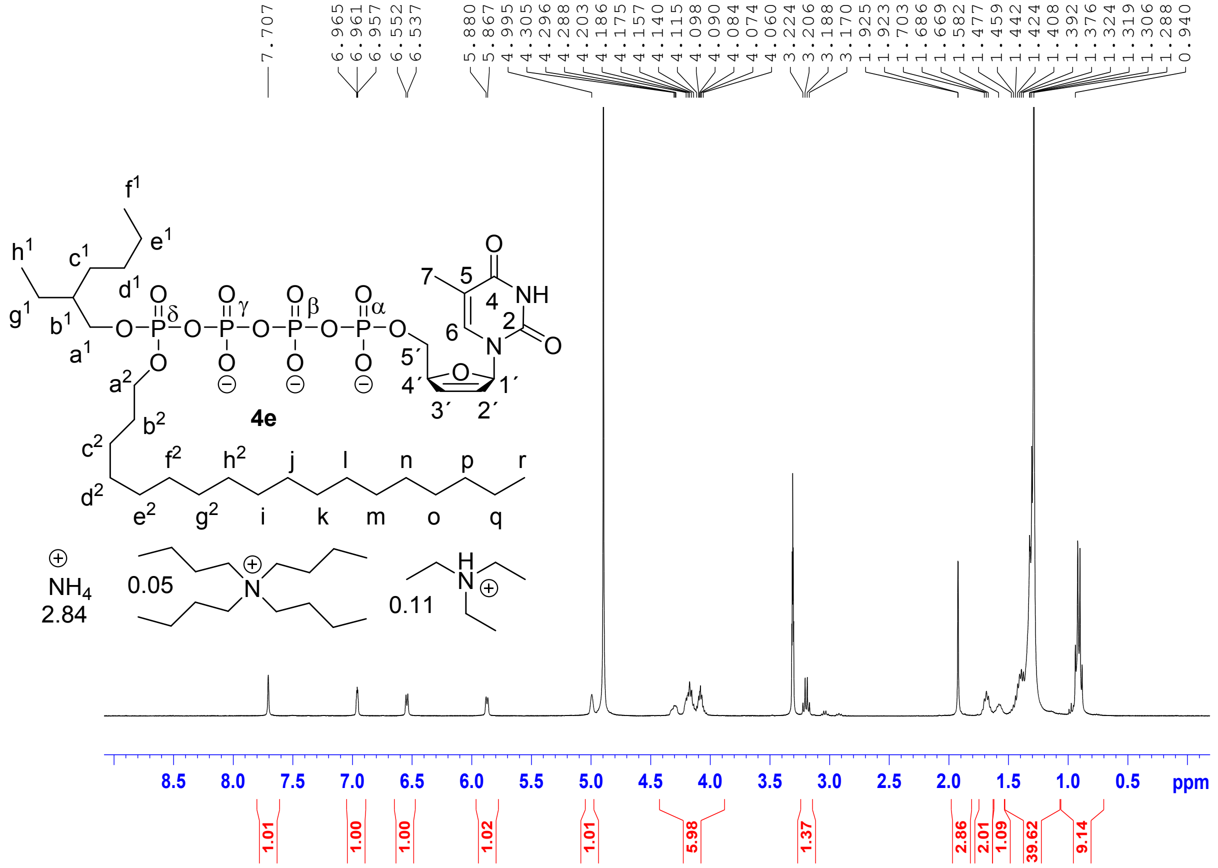

7.707
6.965
6.961
6.957
6.552
6.537
5.880
5.867
4.995
4.305
4.296
4.288
4.203
4.186
4.175
4.157
4.140
4.115
4.098
4.090
4.084
4.074
4.060
3.224
3.206
3.188
3.170
1.925
1.923
1.703
1.686
1.669
1.582
1.477
1.459
1.442
1.424
1.408
1.392
1.376
1.324
1.319
1.306
1.288
0.940
4e
NH4
2.84
0.05
0.11
ppm
1.01
1.00
1.00
1.02
1.01
5.98
1.37
2.86
2.01
1.09
39.62
9.14

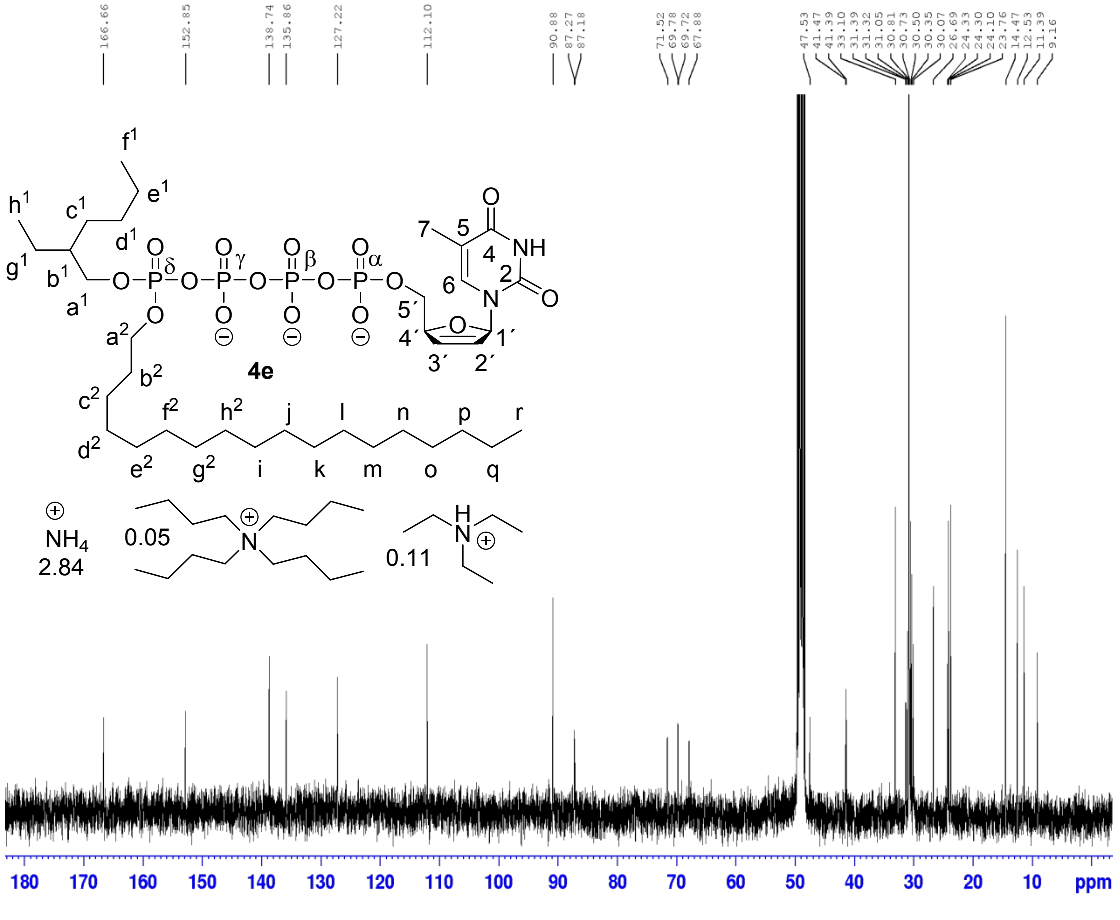

166.66
152.85
138.74
135.86
127.22
112.10
90.88
87.27
87.18
71.52
69.78
69.72
67.88
47.53
41.47
41.39
33.10
31.39
31.32
31.05
30.81
30.73
30.50
30.35
30.07
26.69
24.33
24.30
24.10
23.76
14.47
12.53
11.39
9.16
4e
NH4
2.84
0.05
0.11
ppm

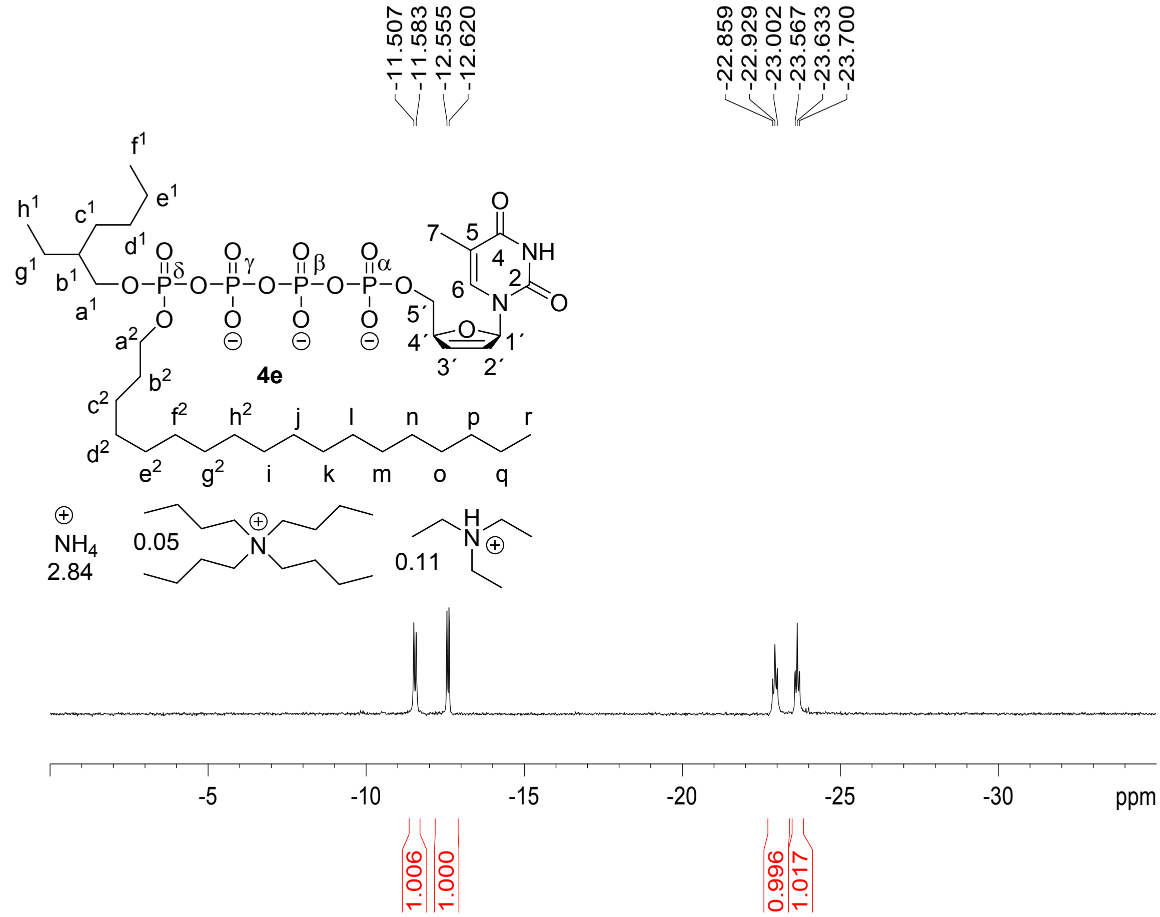
-11.507
-11.583
-12.555
-12.620
-22.859
-22.929
-23.002
-23.567
-23.633
-23.700
4e
NH4
2.84
0.05
0.11
-5
-10
-15
-20
-25
-30
ppm
1.006
1.000
0.996
1.017

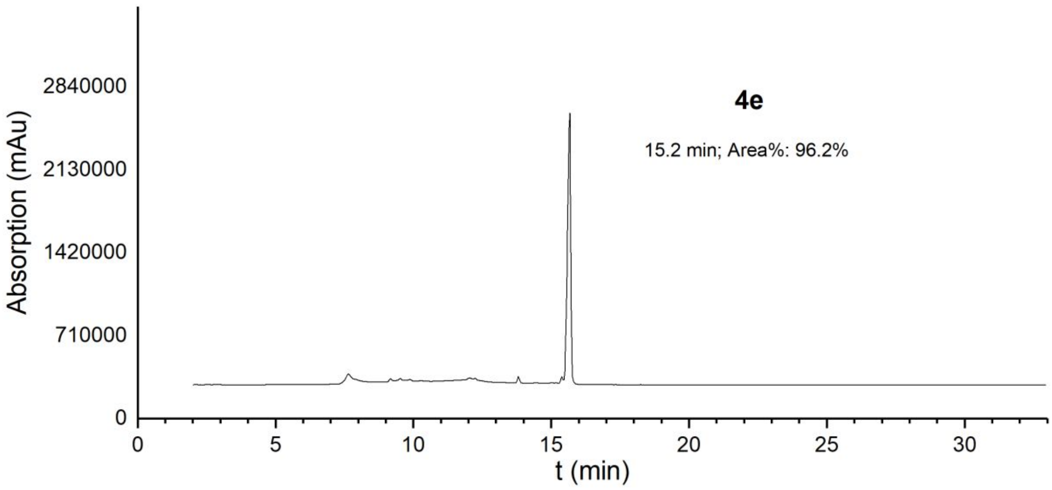
4e
15.2 min; Area%: 96.2%
Absorption (mAu)
2840000
2130000
1420000
710000
0
0
5
10
15
20
25
30
t (min)

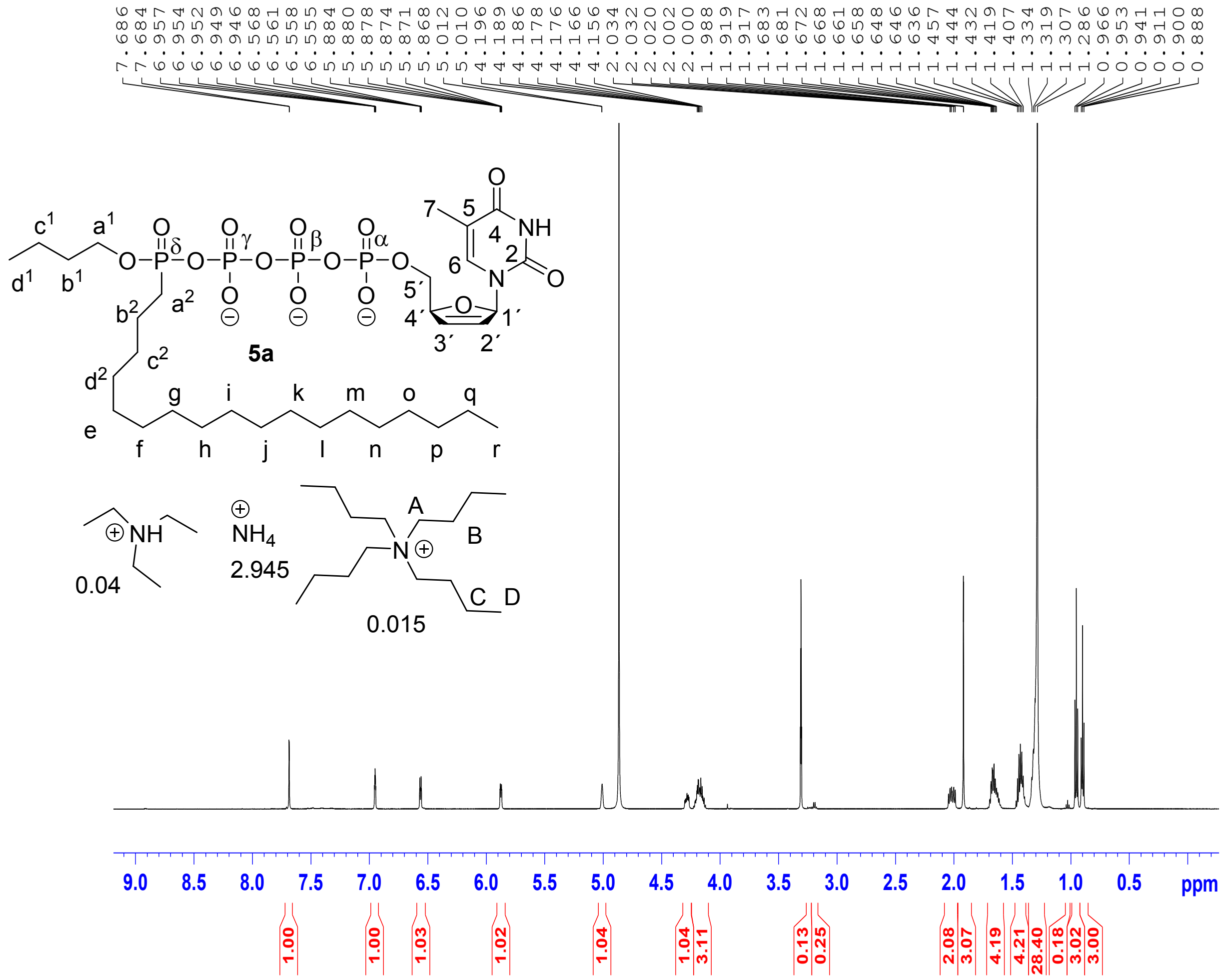

5a
0.04
2.945
0.015
ppm

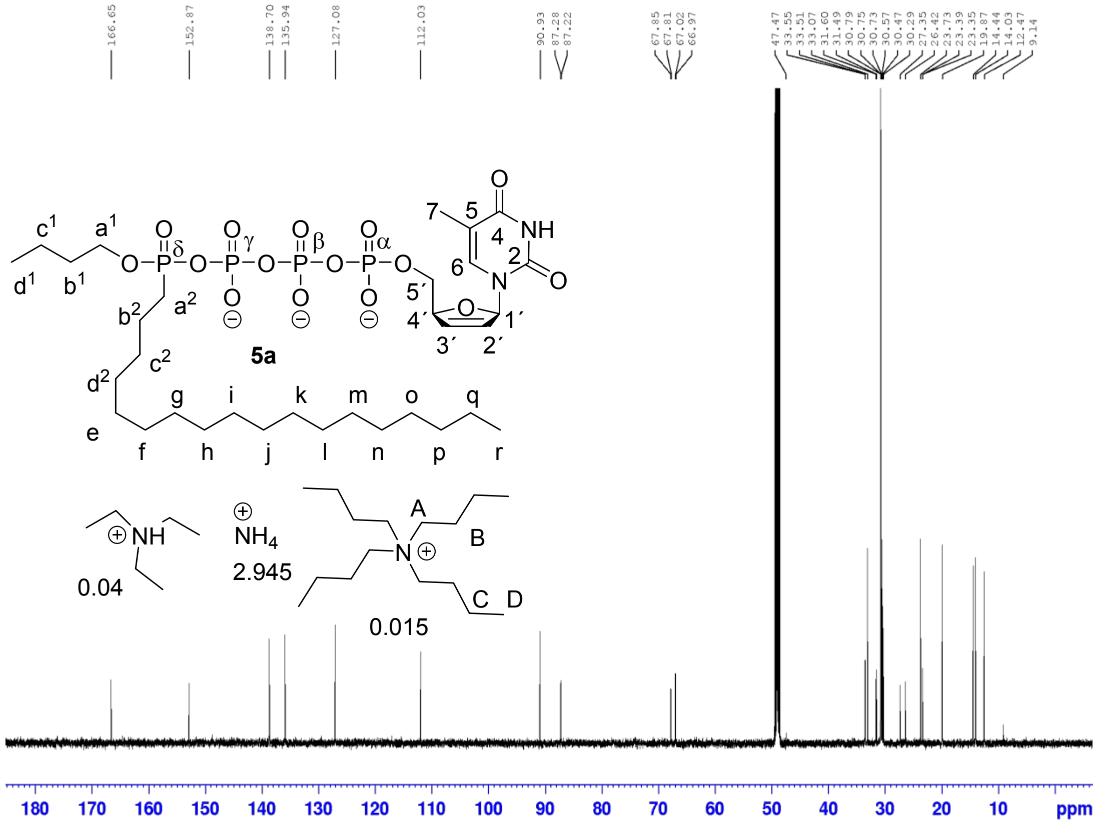

5a
0.04
2.945
0.015
ppm

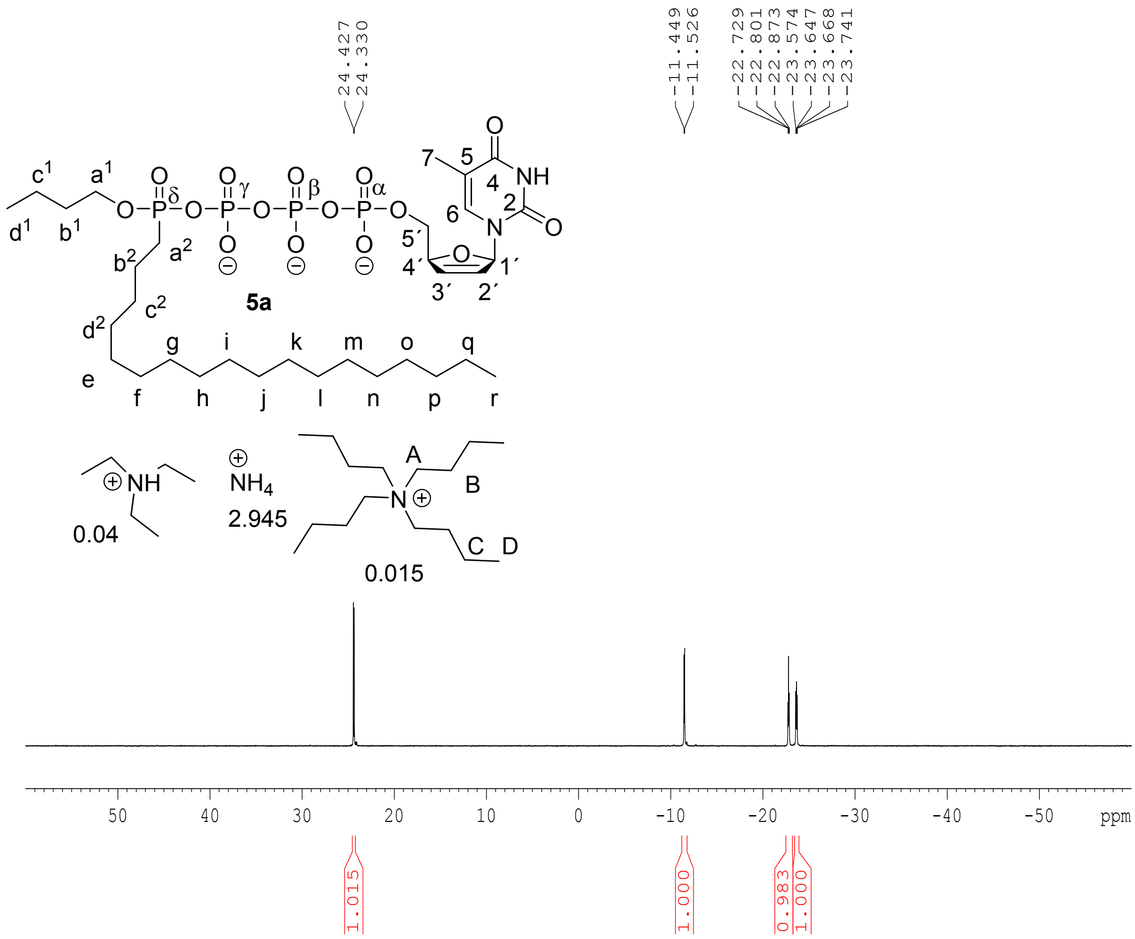
24.427
24.330
-11.449
-11.526
-22.729
-22.801
-22.873
-23.574
-23.647
-23.668
-23.741
5a
0.04
2.945
0.015
ppm
1.015
1.000
0.983
1.000

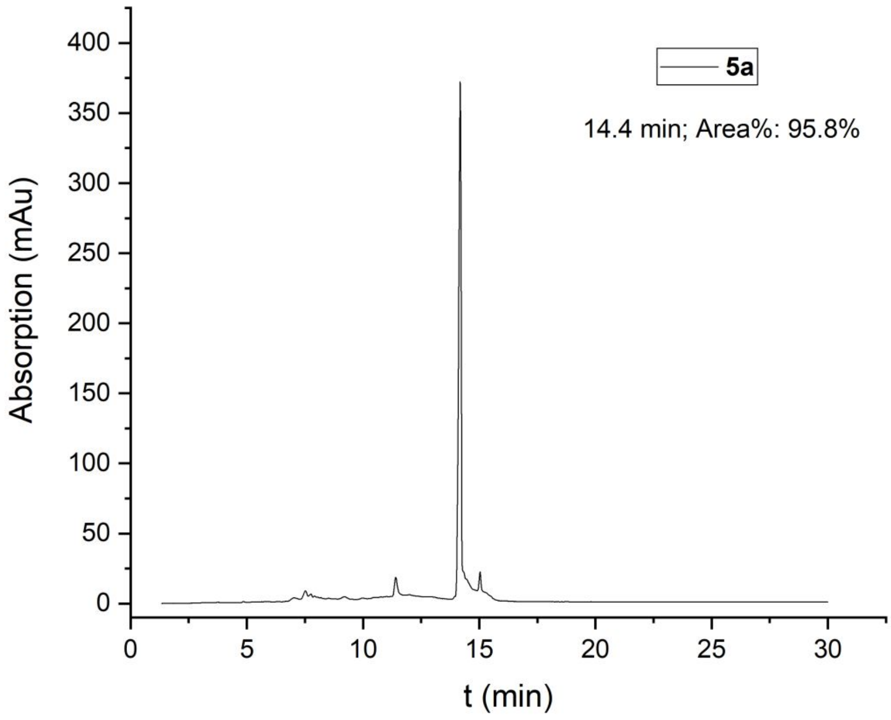
5a
14.4 min; Area%: 95.8%
Absorption (mAu)
t (min)

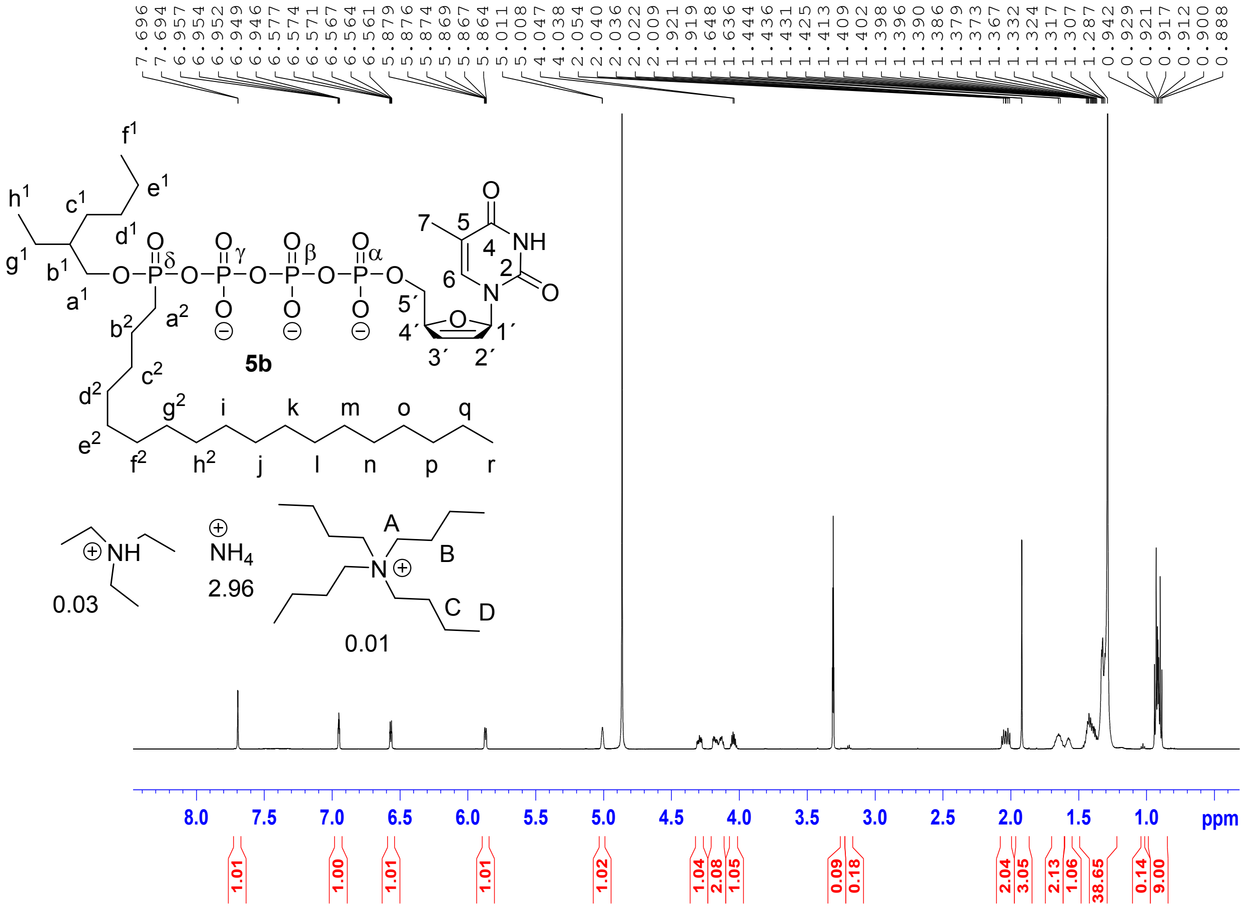

7.696 7.694 6.957 6.954 6.952 6.949 6.946 6.577 6.574 6.571 6.567 6.564 6.561 5.879 5.876 5.874 5.869 5.867 5.864 5.011 5.008 4.047 4.038 2.054 2.040 2.036 2.022 2.009 1.921 1.919 1.648 1.636 1.444 1.436 1.431 1.425 1.413 1.409 1.402 1.398 1.396 1.390 1.386 1.379 1.373 1.367 1.332 1.324 1.317 1.307 1.287 0.942 0.929 0.921 0.917 0.912 0.900 0.888
5b
0.03
2.96
0.01
8.0 7.5 7.0 6.5 6.0 5.5 5.0 4.5 4.0 3.5 3.0 2.5 2.0 1.5 1.0 ppm
1.01 1.00 1.01 1.01 1.02 1.04 2.08 1.05 0.09 0.18 2.04 3.05 2.13 1.06 38.65 0.14 9.00

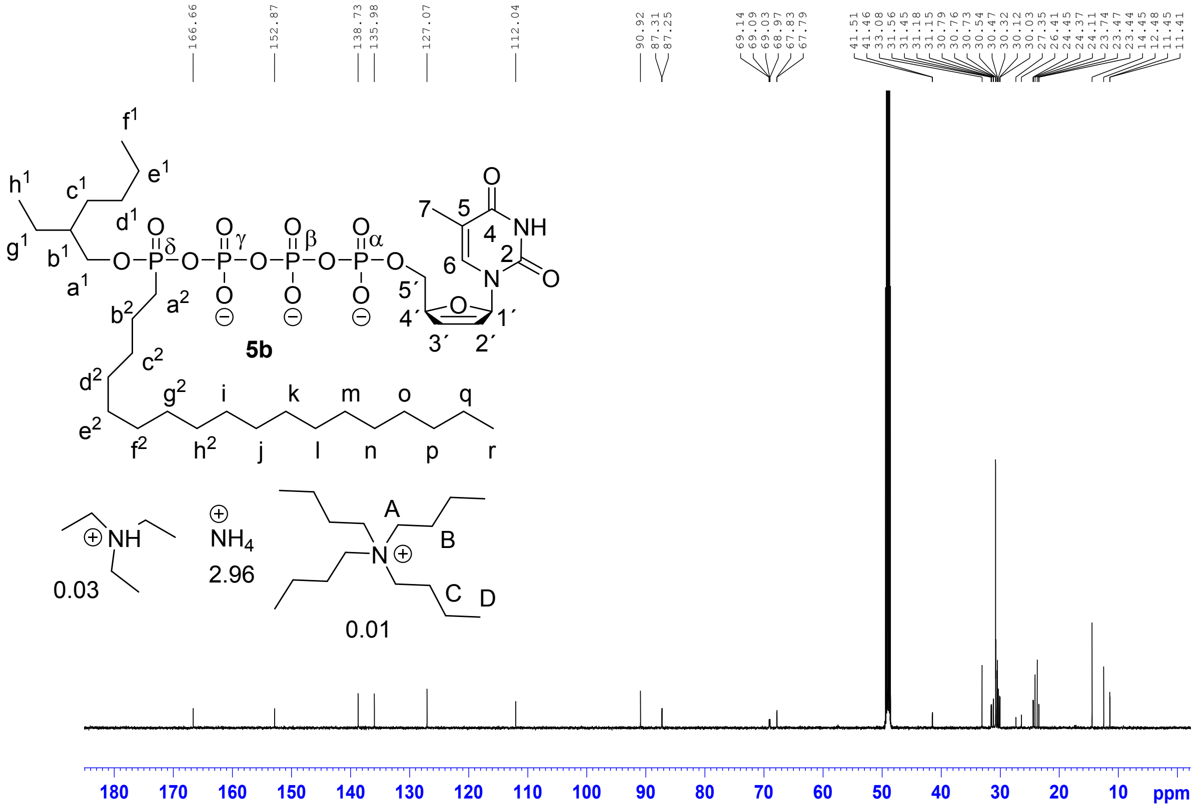

166.66 152.87 138.73 135.98 127.07 112.04 90.92 87.31 87.25 69.14 69.09 69.03 68.97 67.83 67.79 41.51 41.46 33.08 31.56 31.45 31.18 31.15 30.79 30.76 30.73 30.54 30.47 30.32 30.12 30.03 27.35 26.41 24.45 24.37 24.11 23.74 23.47 23.44 14.45 12.48 11.45 11.41
5b
0.03
2.96
0.01
180 170 160 150 140 130 120 110 100 90 80 70 60 50 40 30 20 10 ppm

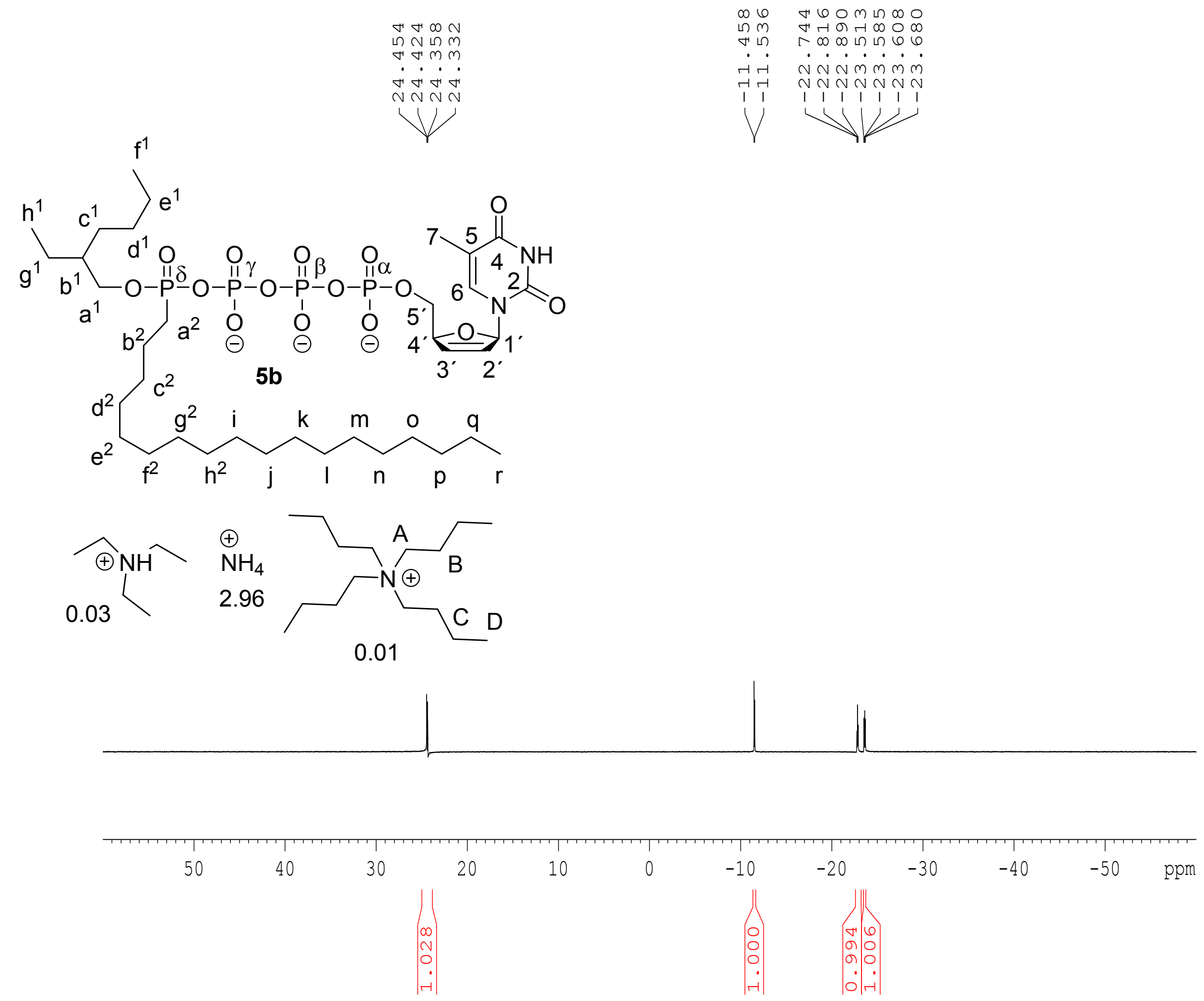
24.454
24.424
24.358
24.332
-11.458
-11.536
-22.744
-22.816
-22.890
-23.513
-23.585
-23.608
-23.680
5b
0.03
2.96
0.01
ppm
1.028
1.000
0.994
1.006

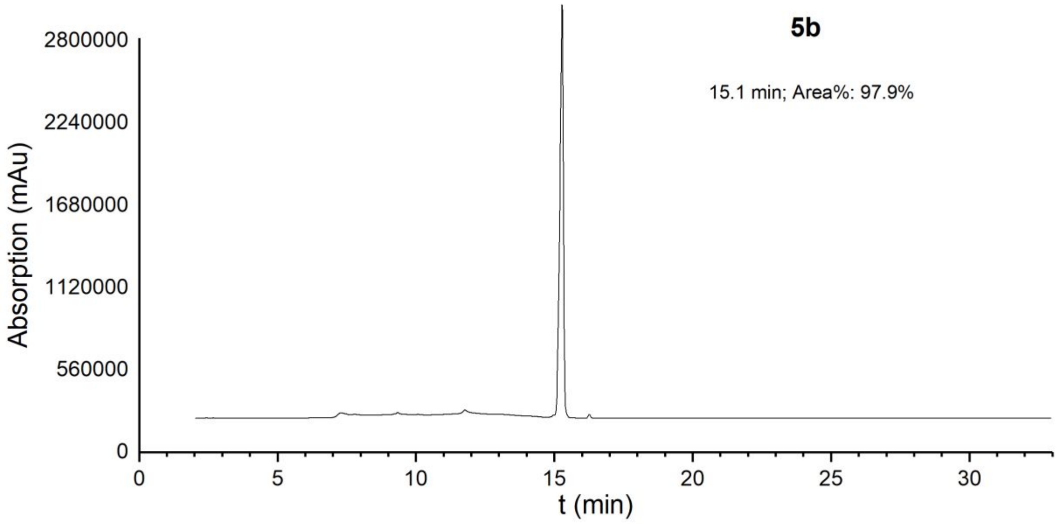
5b
15.1 min; Area%: 97.9%
Absorption (mAu)
t (min)

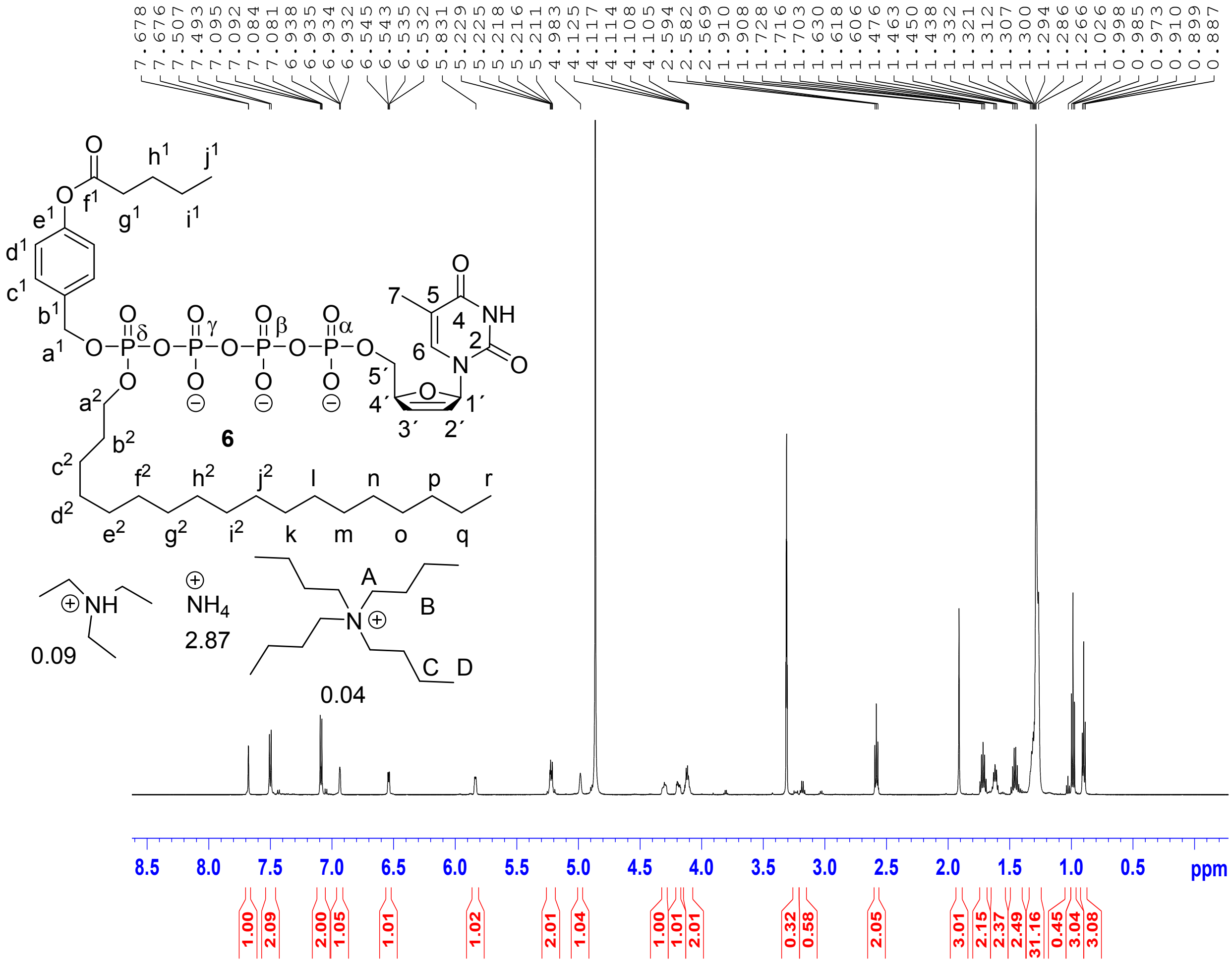
6
0.09
2.87
0.04
ppm
1.00
2.09
2.00
1.05
1.01
1.02
2.01
1.04
1.00
1.01
2.01
0.32
0.58
2.05
3.01
2.15
2.37
2.49
31.16
0.45
3.04
3.08

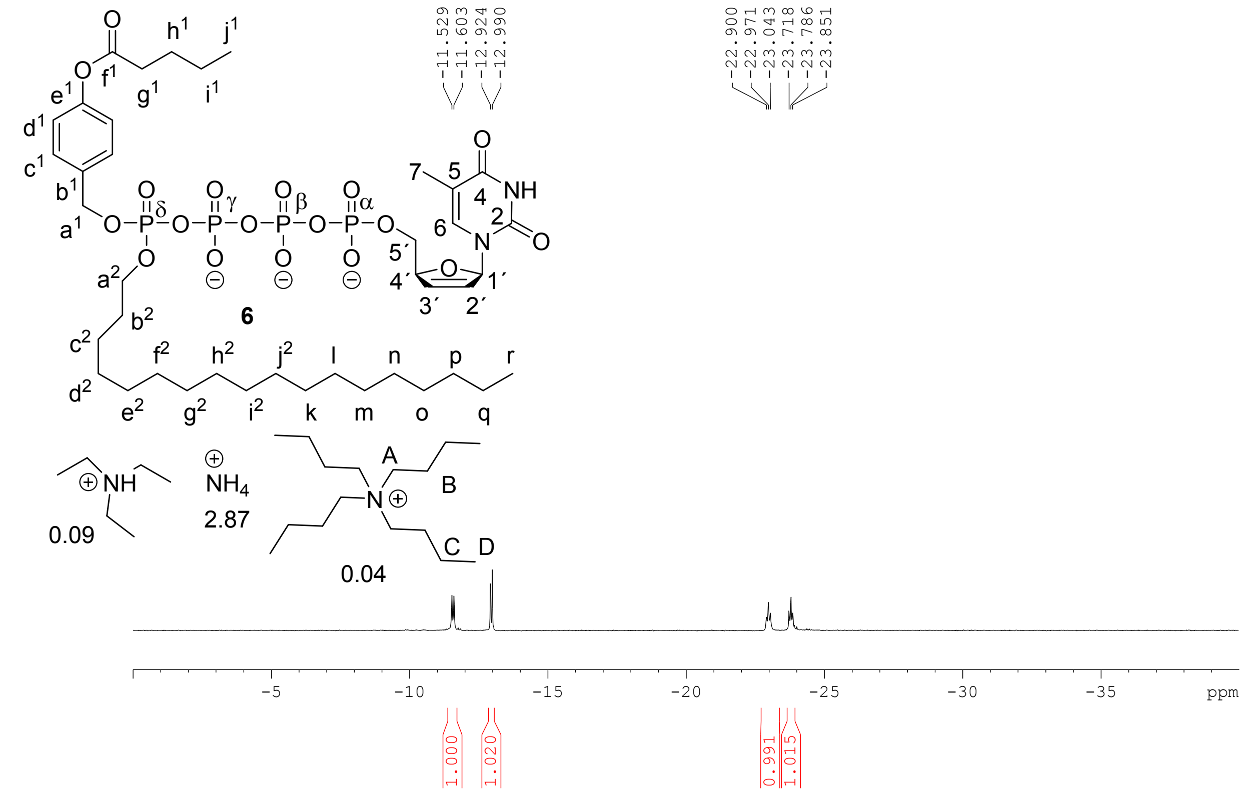
6
0.09
2.87
0.04
-11.529
-11.603
-12.924
-12.990
-22.900
-22.971
-23.043
-23.718
-23.786
-23.851
ppm
1.000
1.020
0.991
1.015

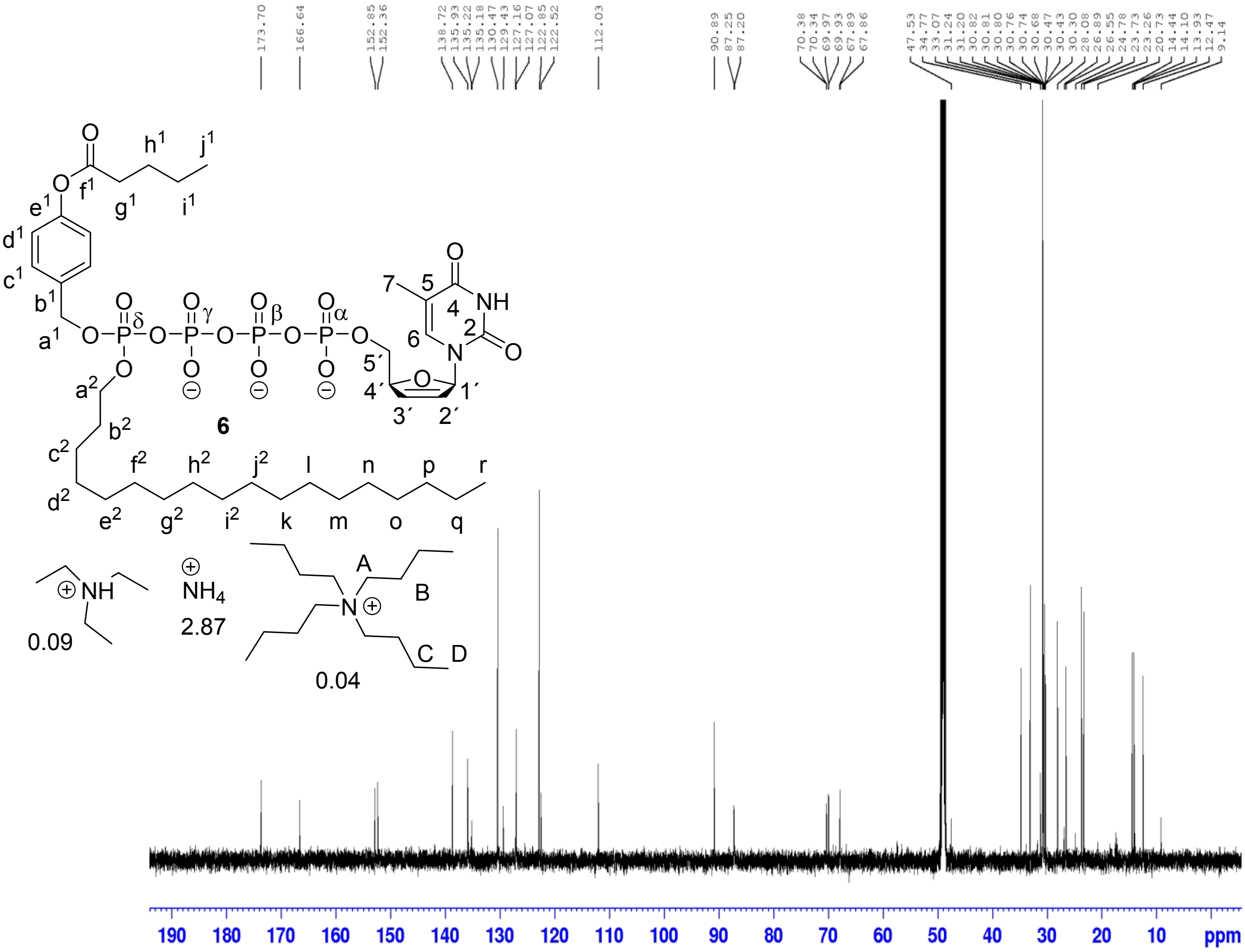
173.70 166.64 152.85 152.36 138.72 135.93 135.22 135.18 130.47 129.43 127.16 127.07 122.85 122.52 112.03 90.89 87.25 87.20 70.38 70.34 69.97 69.93 67.89 67.86 47.53 34.77 33.07 31.24 31.20 30.82 30.81 30.80 30.76 30.74 30.68 30.47 30.43 30.30 28.08 26.89 26.55 24.78 23.73 23.26 20.73 14.44 14.10 13.93 12.47 9.14
6
0.09
2.87
0.04
ppm

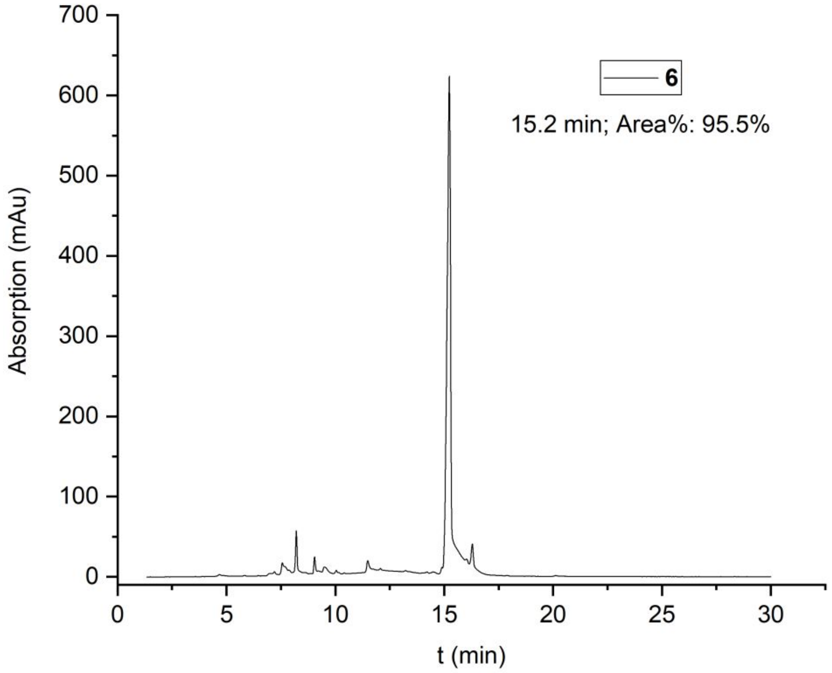
6
15.2 min; Area%: 95.5%
Absorption (mAu)
t (min)

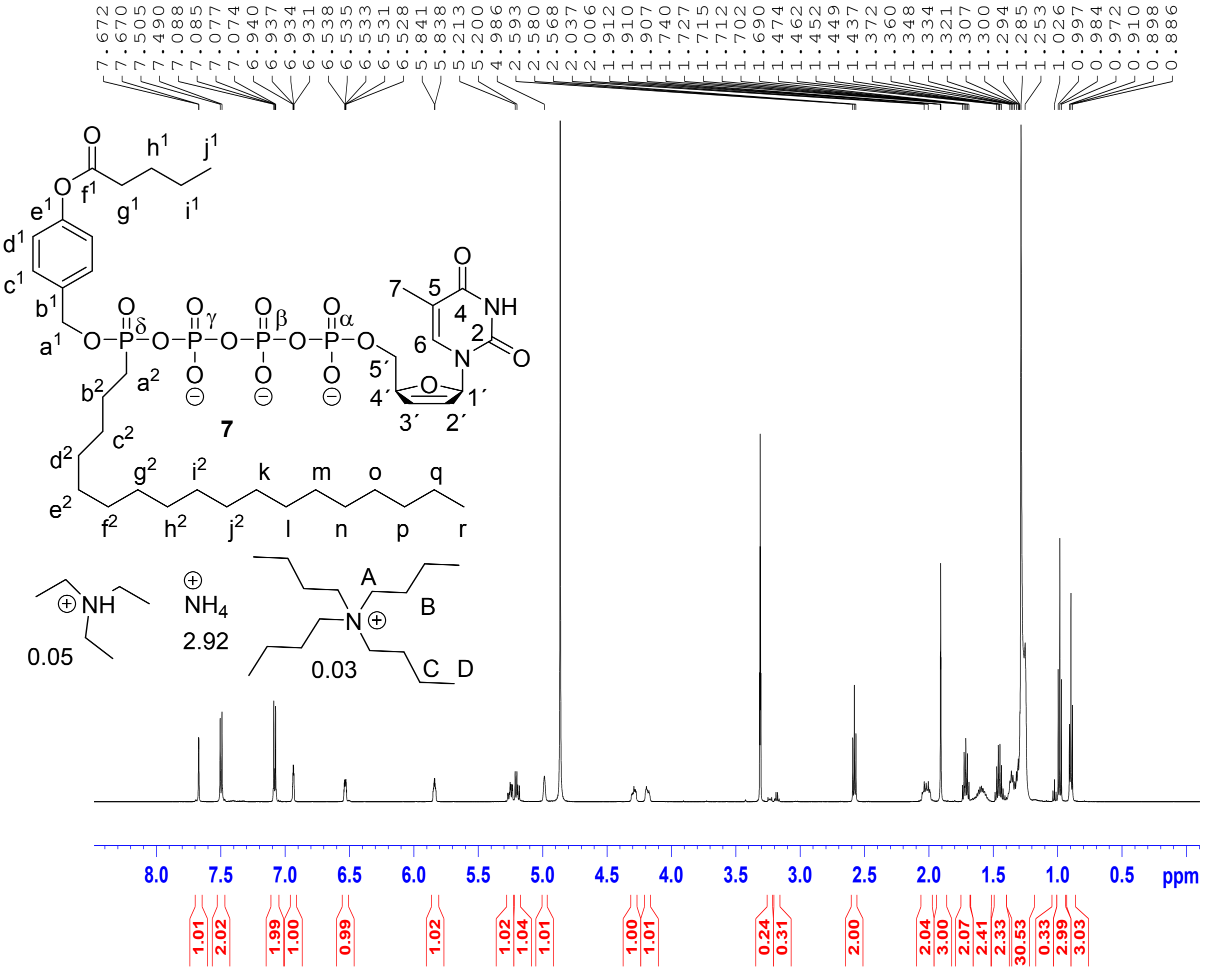

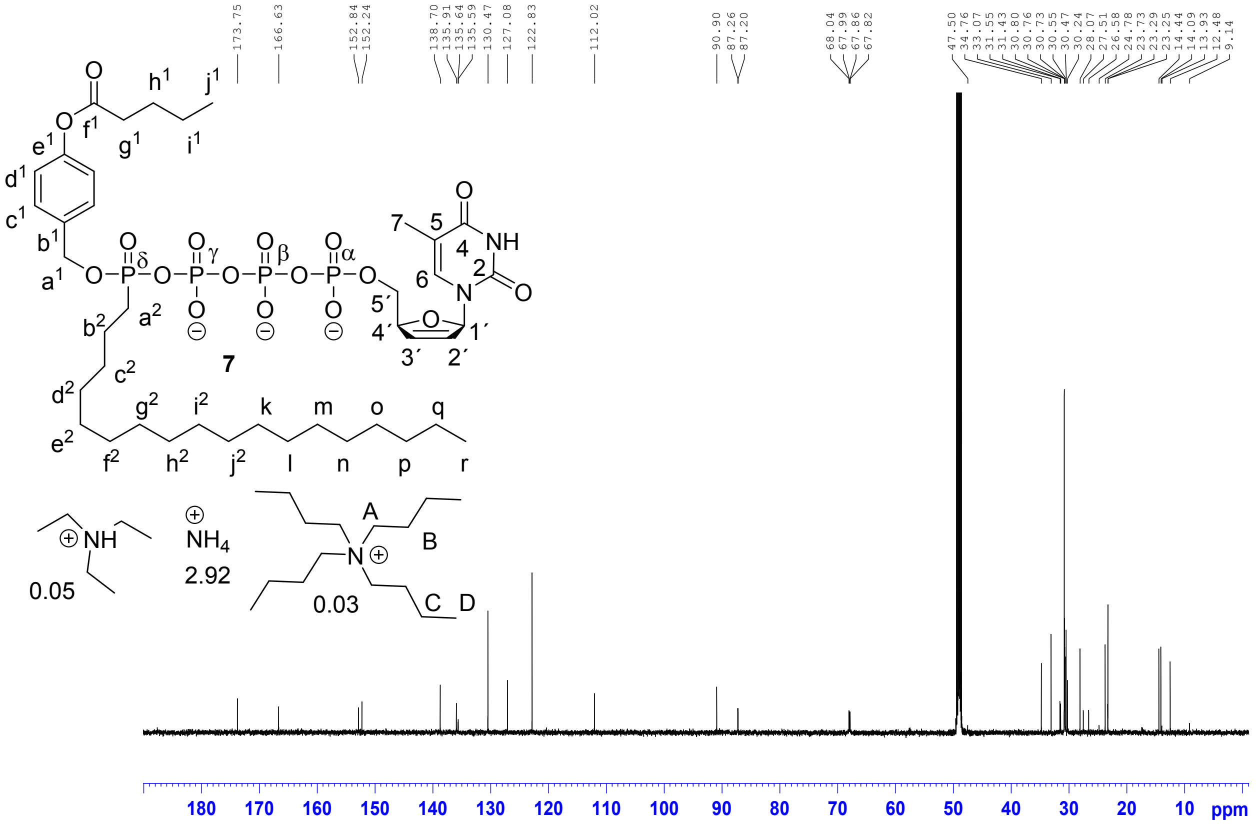

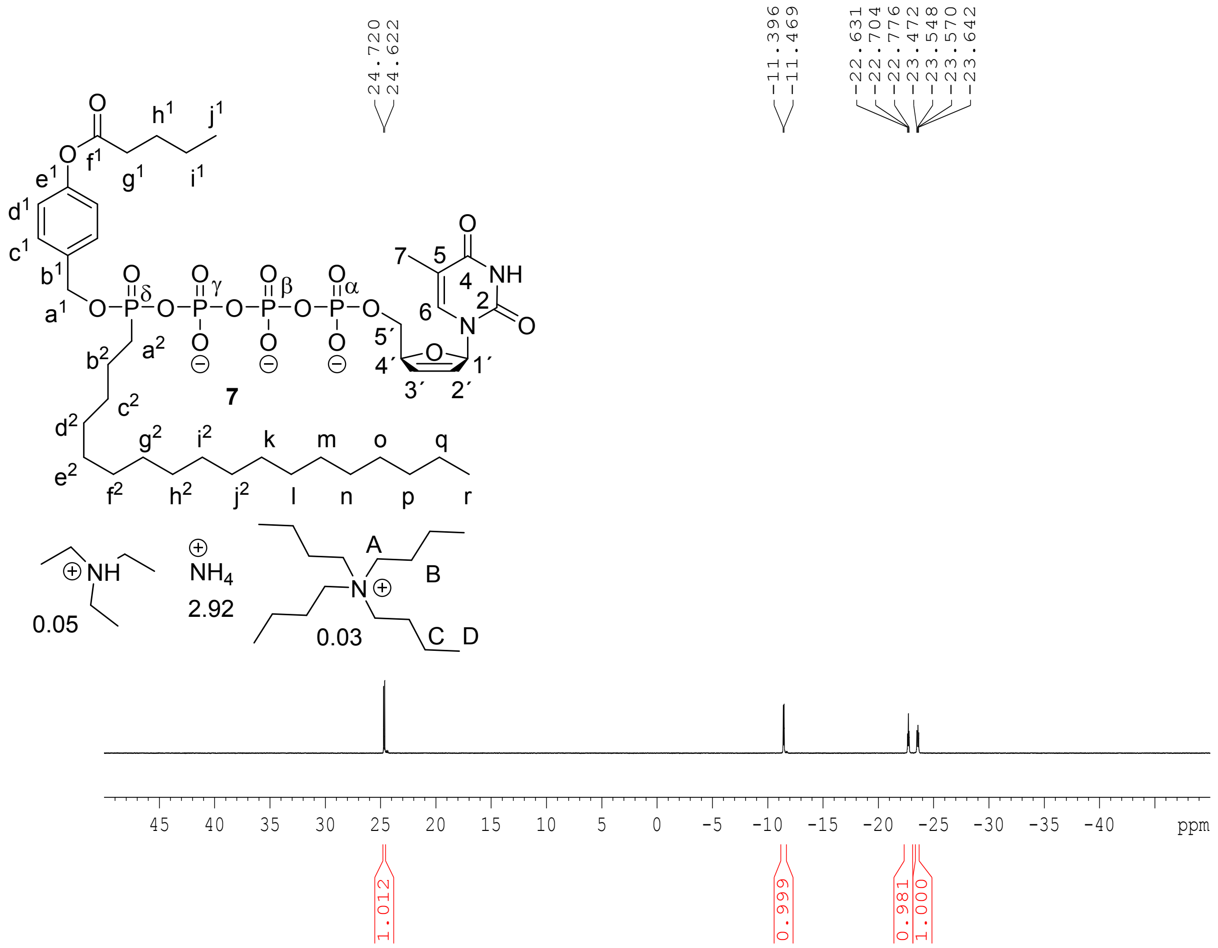

24.720
24.622
-11.396
-11.469
-22.631
-22.704
-22.776
-23.472
-23.548
-23.570
-23.642
7
0.05
2.92
0.03
ppm
1.012
0.999
0.981
1.000

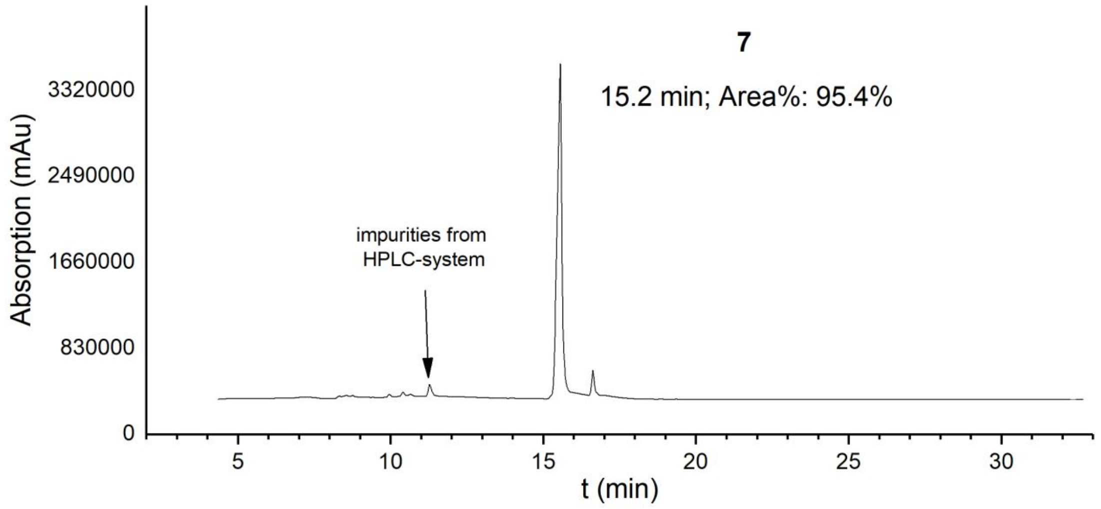

7
15.2 min; Area%: 95.4%
impurities from HPLC-system
Absorption (mAu)
t (min)

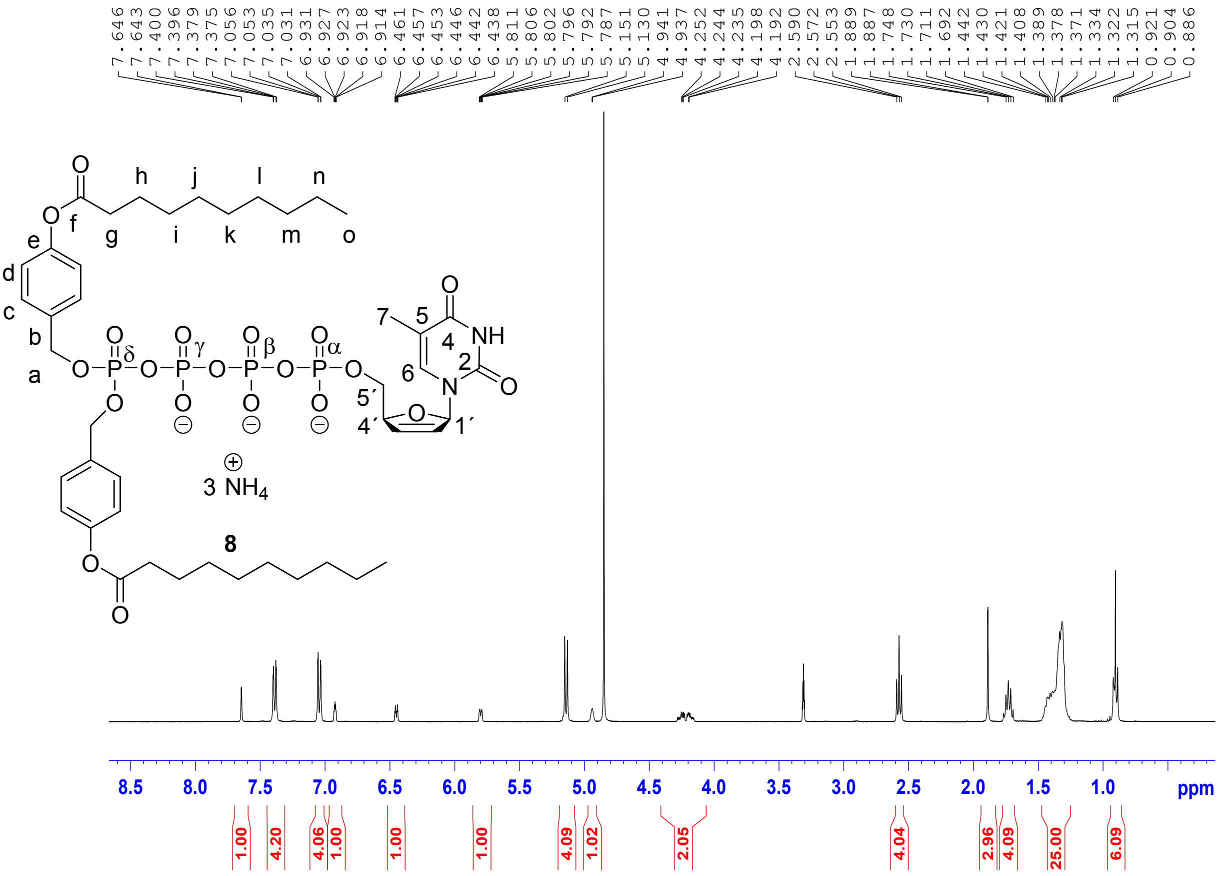
7.646 7.643 7.400 7.396 7.379 7.375 7.056 7.053 7.035 7.031 6.931 6.927 6.923 6.918 6.914 6.461 6.457 6.453 6.446 6.442 6.438 5.811 5.806 5.802 5.796 5.792 5.787 5.151 5.130 4.941 4.937 4.252 4.244 4.235 4.198 4.192 2.590 2.572 2.553 1.887 1.748 1.730 1.711 1.692 1.442 1.430 1.421 1.408 1.389 1.378 1.371 1.334 1.322 1.315 0.921 0.904 0.886
h j l n
f g i k m o
e d c b a
δ γ β α
7 5 4 NH 2 6 N O
5´ 4´ 1´
3 NH4
8
8.5 8.0 7.5 7.0 6.5 6.0 5.5 5.0 4.5 4.0 3.5 3.0 2.5 2.0 1.5 1.0 ppm
1.00 4.20 4.06 1.00 1.00 1.00 4.09 1.02 2.05 4.04 2.96 4.09 25.00 6.09

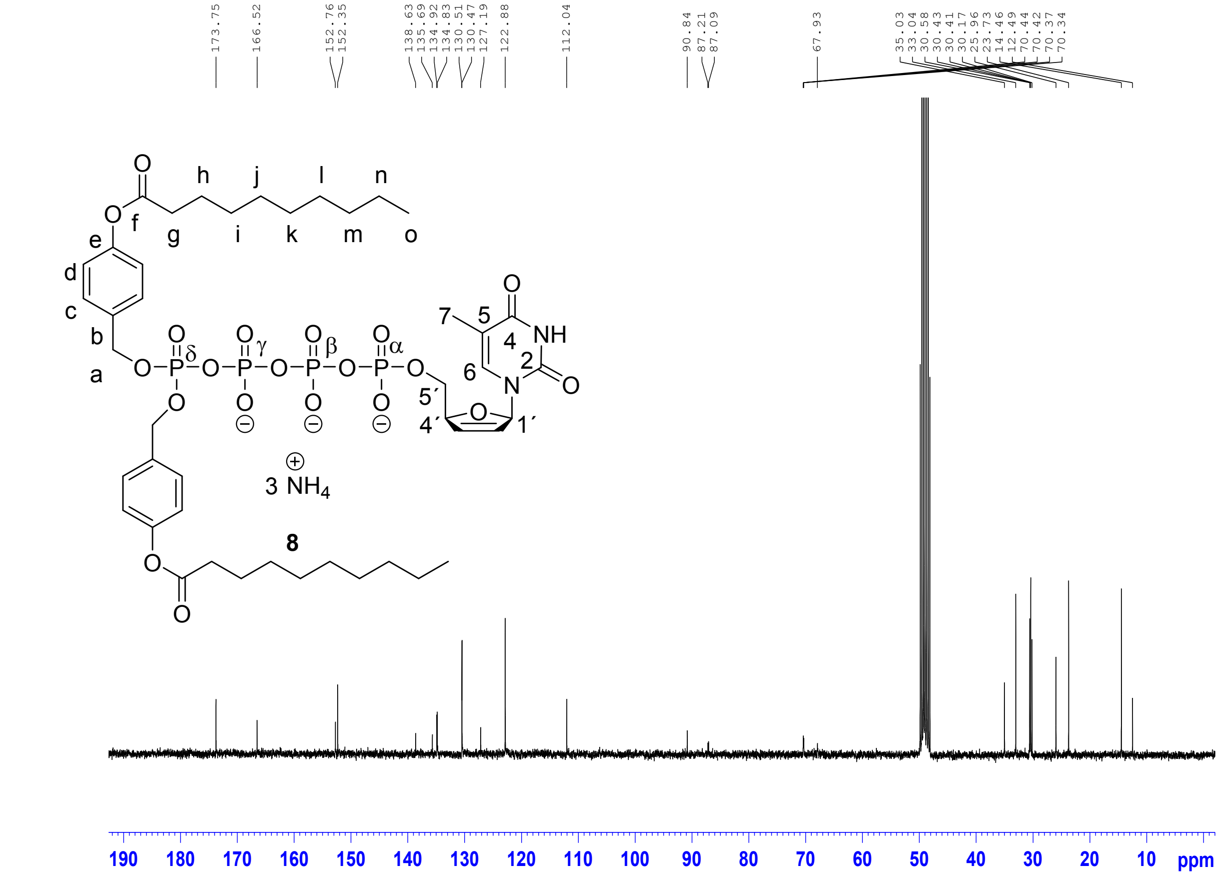
173.75 166.52 152.76 152.35 138.63 135.69 134.92 134.83 130.51 130.47 127.19 122.88 112.04 90.84 87.21 87.09 67.93 35.03 33.04 30.58 30.43 30.41 30.17 25.96 23.73 14.46 12.49 70.44 70.42 70.37 70.34
h j l n
f g i k m o
e d c b a
δ γ β α
7 5 4 NH 2 6 N O
5´ 4´ 1´
3 NH4
8
190 180 170 160 150 140 130 120 110 100 90 80 70 60 50 40 30 20 10 ppm

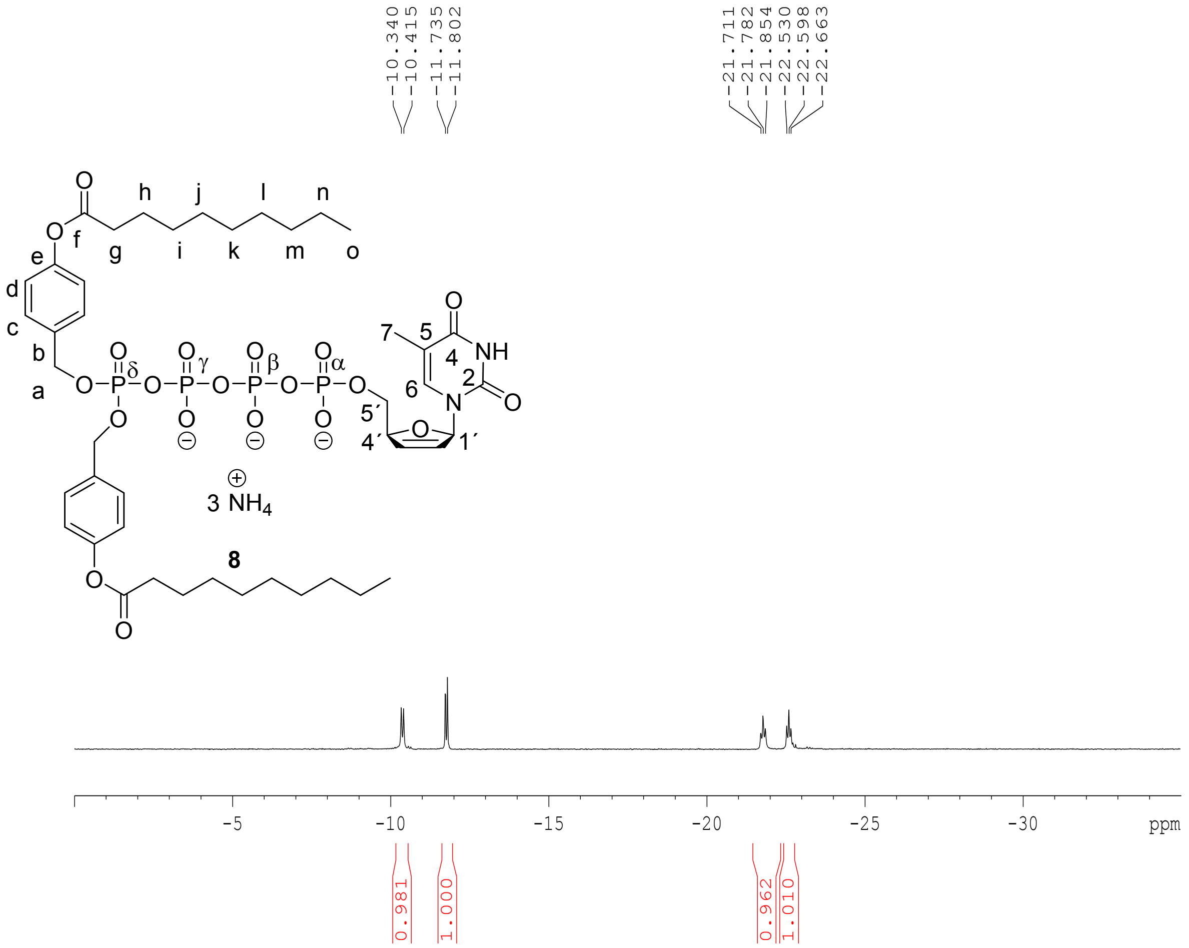
-10.340
-10.415
-11.735
-11.802
-21.711
-21.782
-21.854
-22.530
-22.598
-22.663
8
3 NH4
-5
-10
-15
-20
-25
-30
ppm
0.981
1.000
0.962
1.010

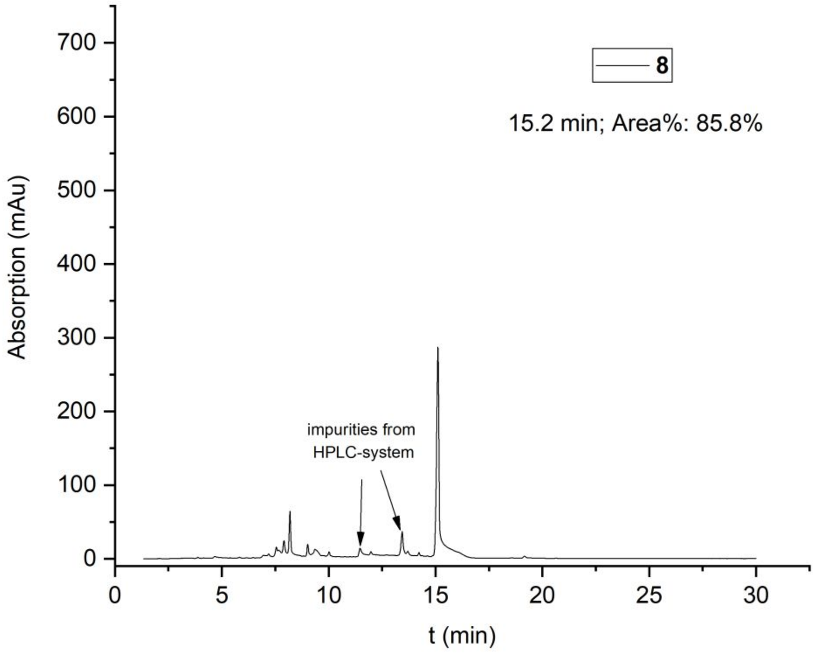
8
15.2 min; Area%: 85.8%
Absorption (mAu)
impurities from
HPLC-system
t (min)

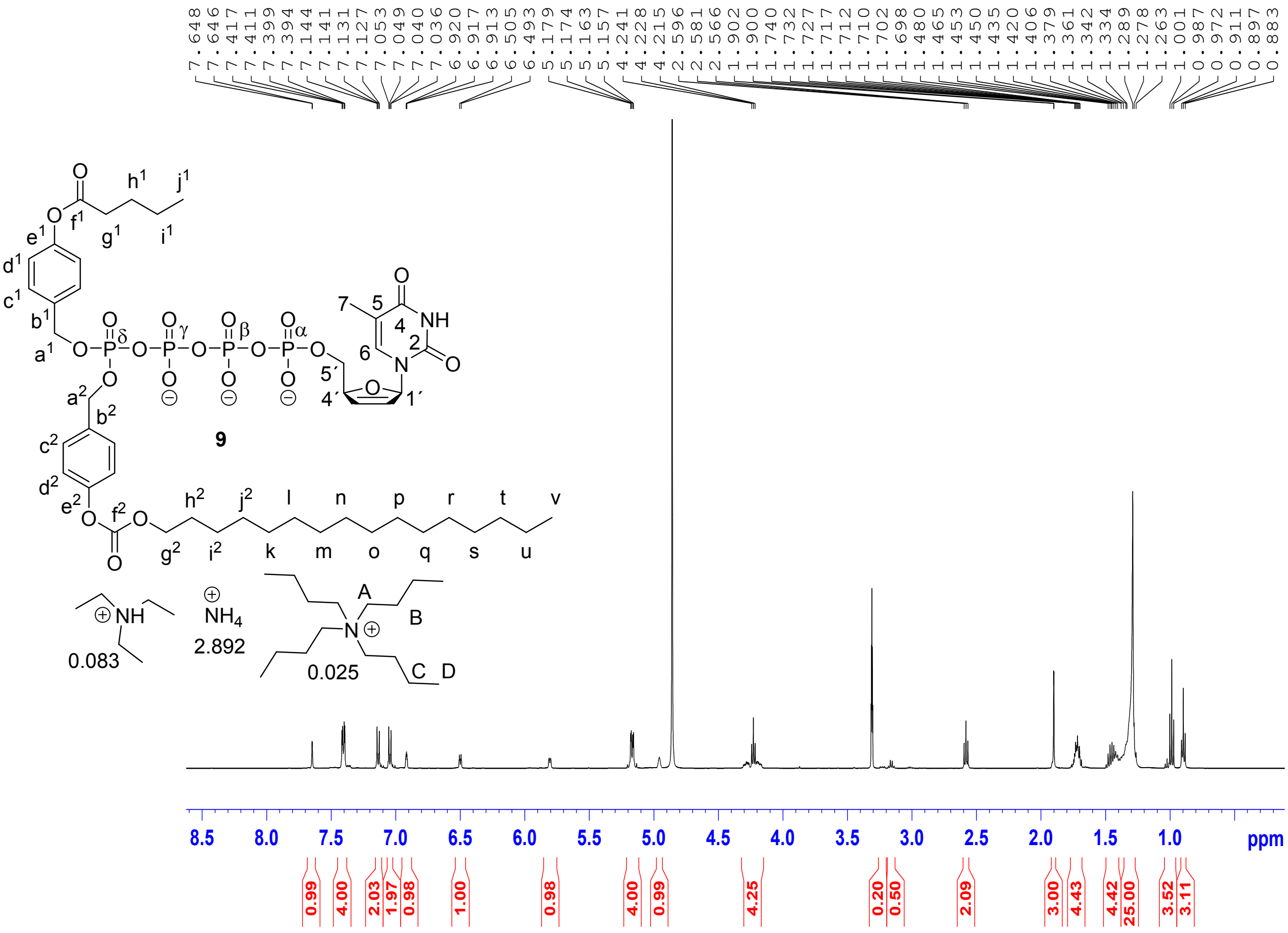
9
0.083
2.892
0.025
ppm

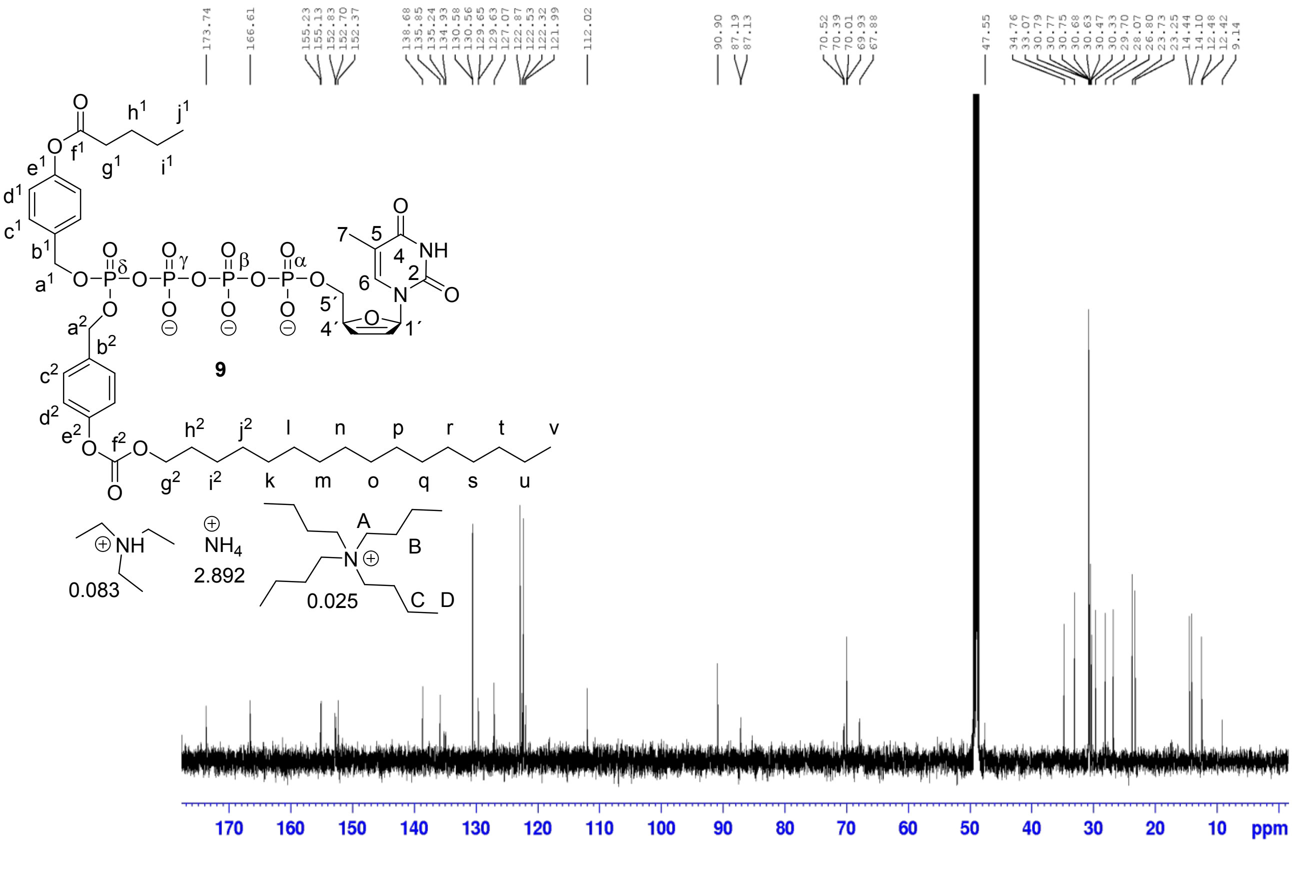
9
0.083
2.892
0.025
ppm

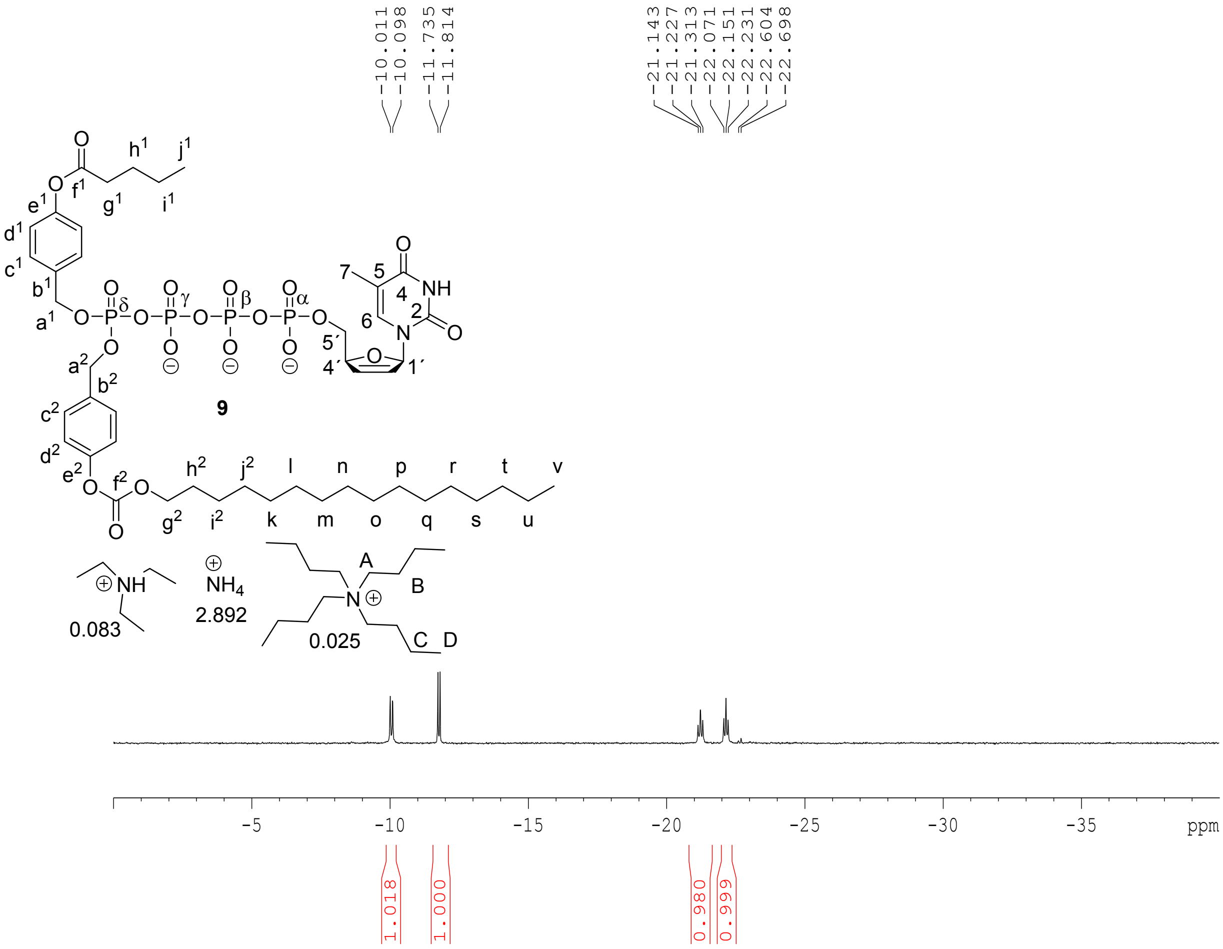

-10.011
-10.098
-11.735
-11.814
-21.143
-21.227
-21.313
-22.071
-22.151
-22.231
-22.604
-22.698
9
0.083
2.892
0.025
-5
-10
-15
-20
-25
-30
-35
ppm
1.018
1.000
0.980
0.999

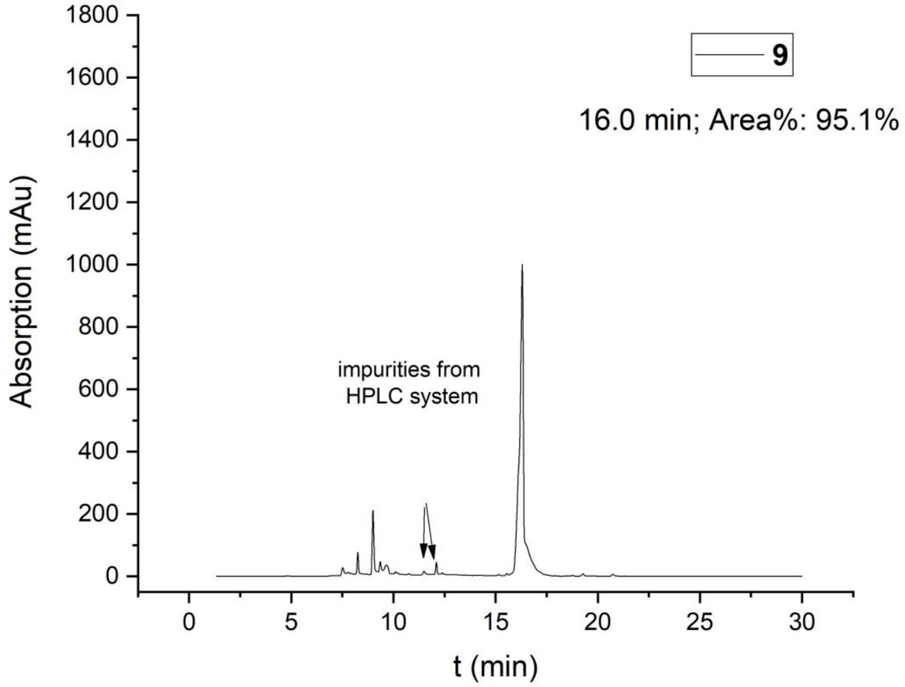

9
16.0 min; Area%: 95.1%
impurities from HPLC system
Absorption (mAu)
t (min)

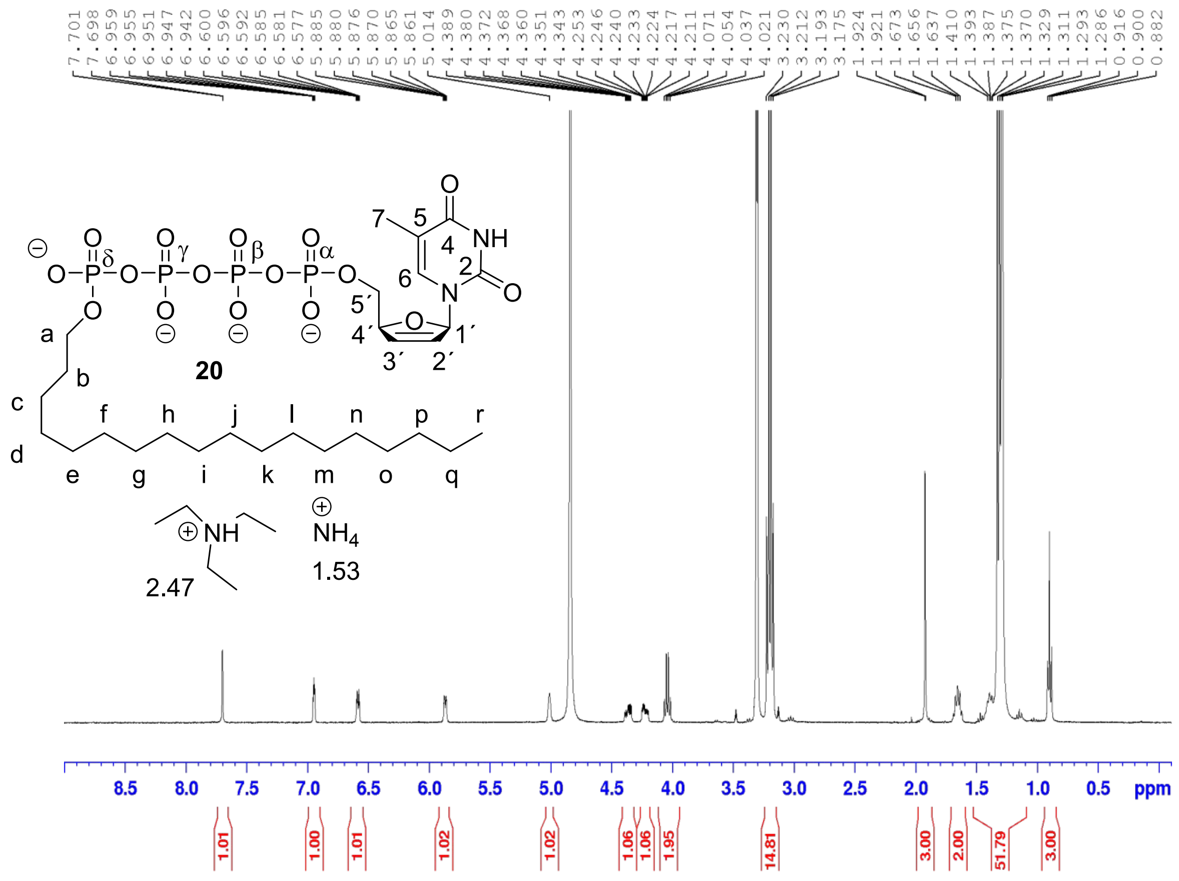

20
2.47
1.53

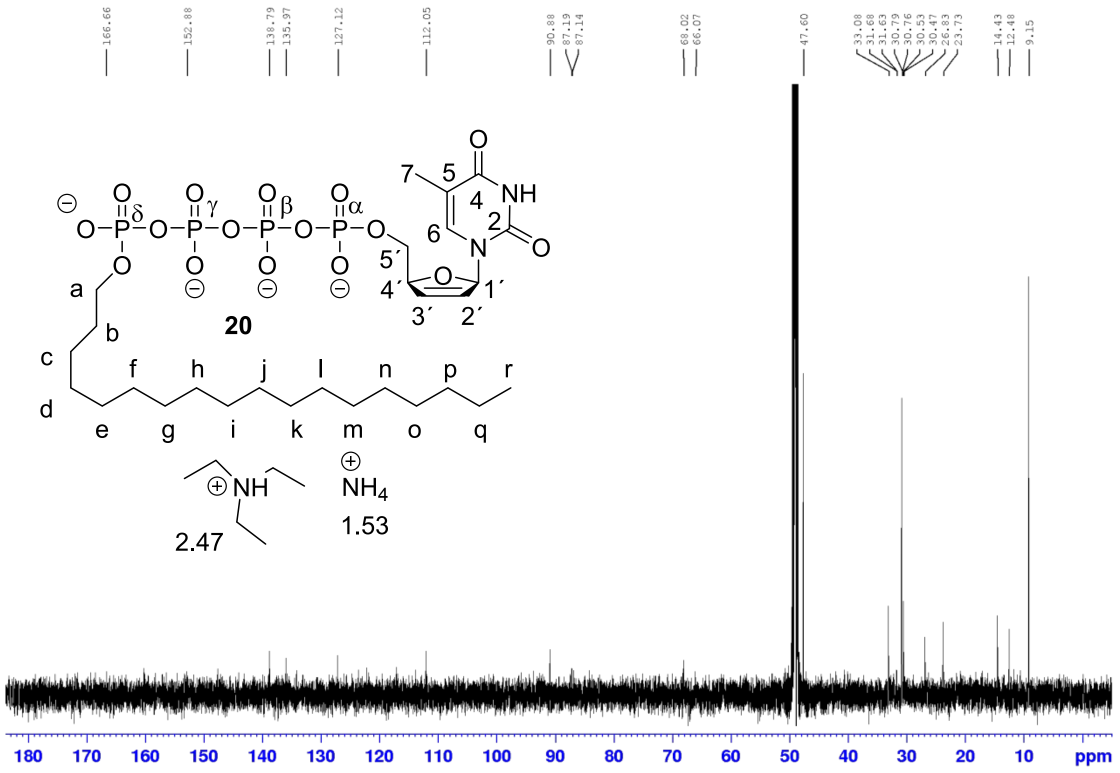

20
2.47
1.53

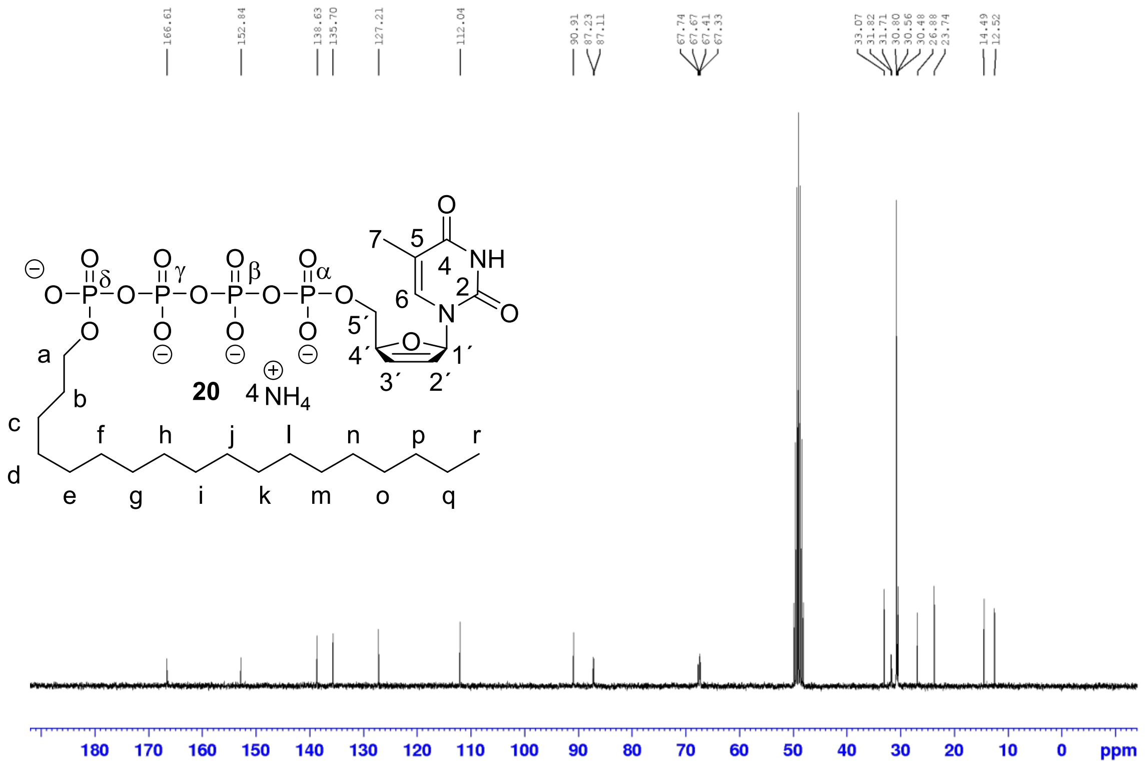
166.61
152.84
138.63
135.70
127.21
112.04
90.91
87.23
87.11
67.74
67.67
67.41
67.33
33.07
31.82
31.71
30.80
30.56
30.48
26.88
23.74
14.49
12.52
20
4 NH4
ppm

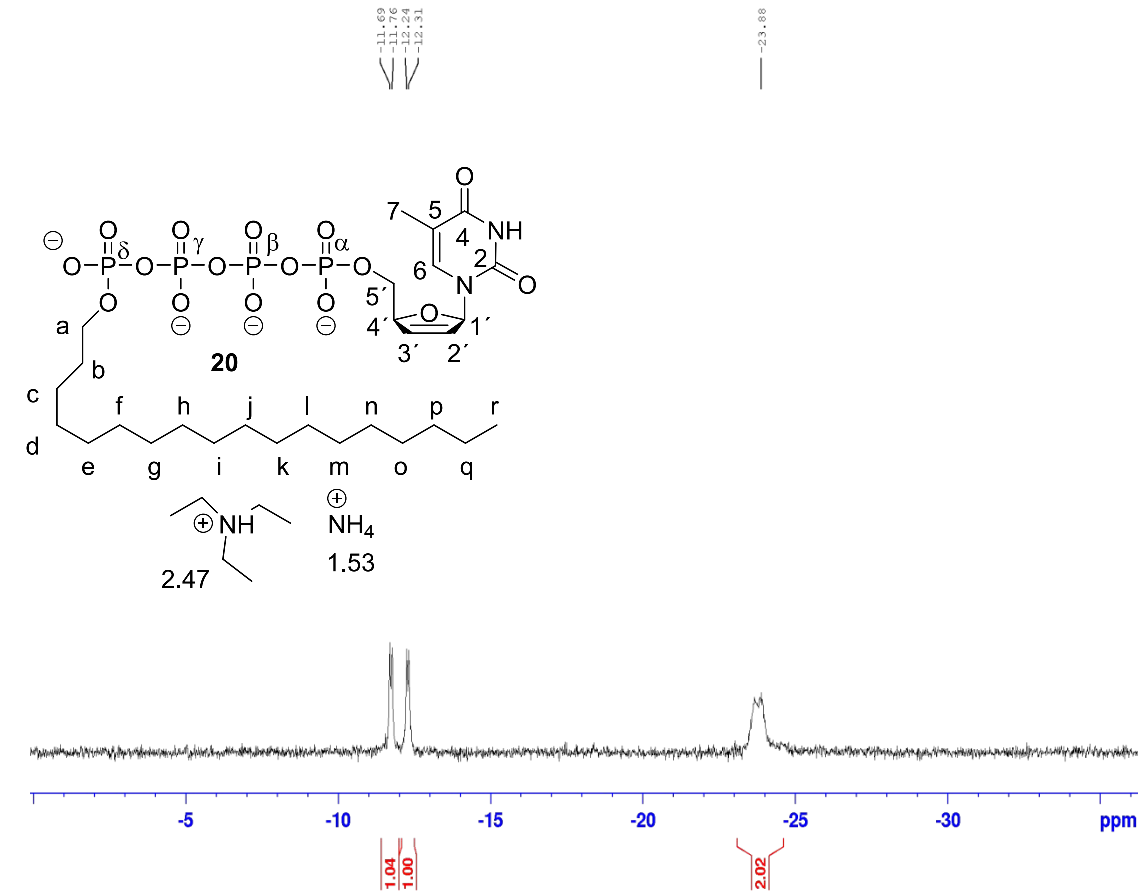
-11.69
-11.76
-12.24
-12.31
-23.88
20
NH
2.47
NH4
1.53
ppm
1.04
1.00
2.02

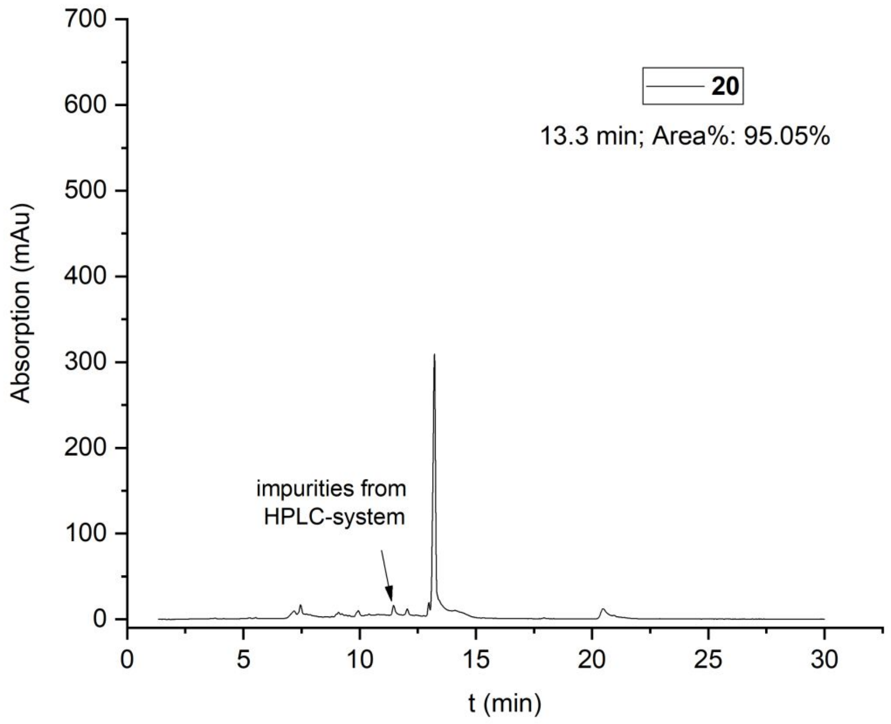

20
13.3 min; Area%: 95.05%
impurities from HPLC-system
Absorption (mAu)
t (min)

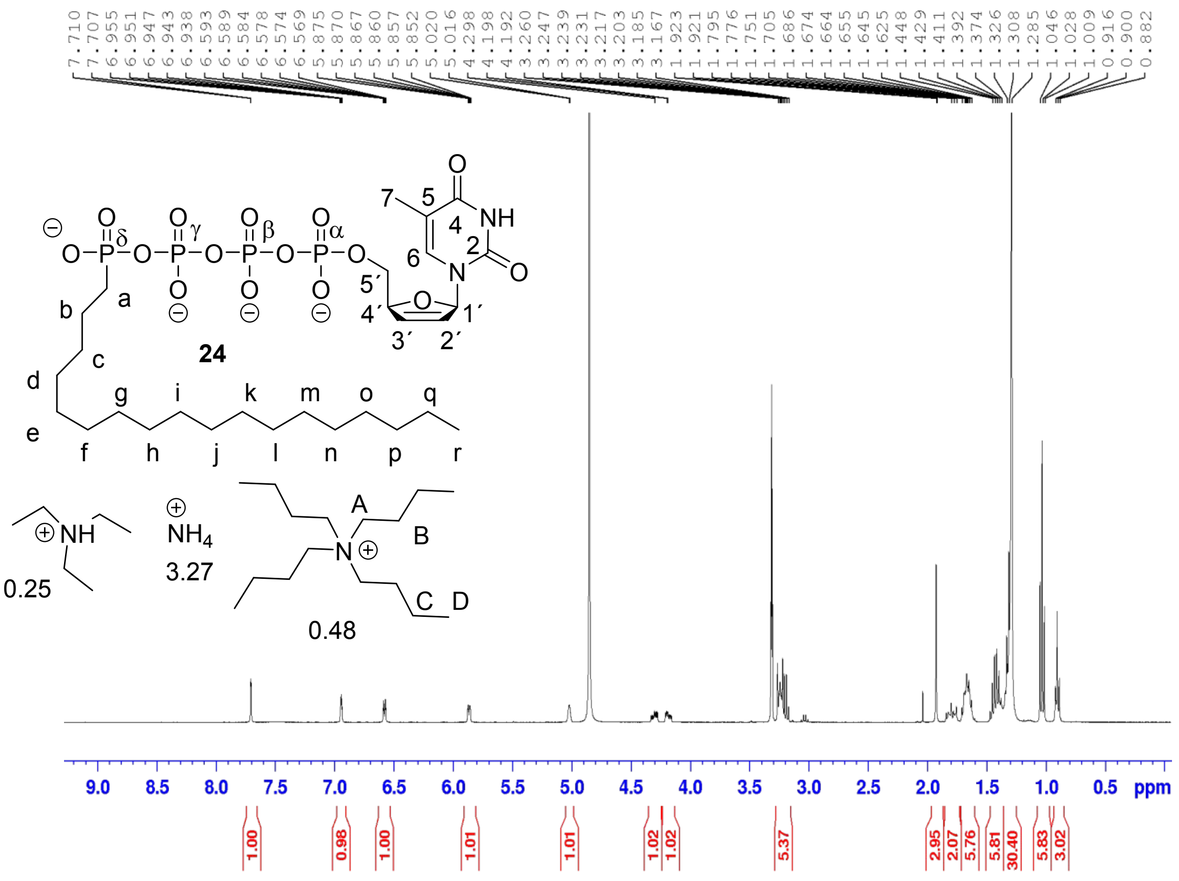

24
0.25
3.27
0.48
ppm

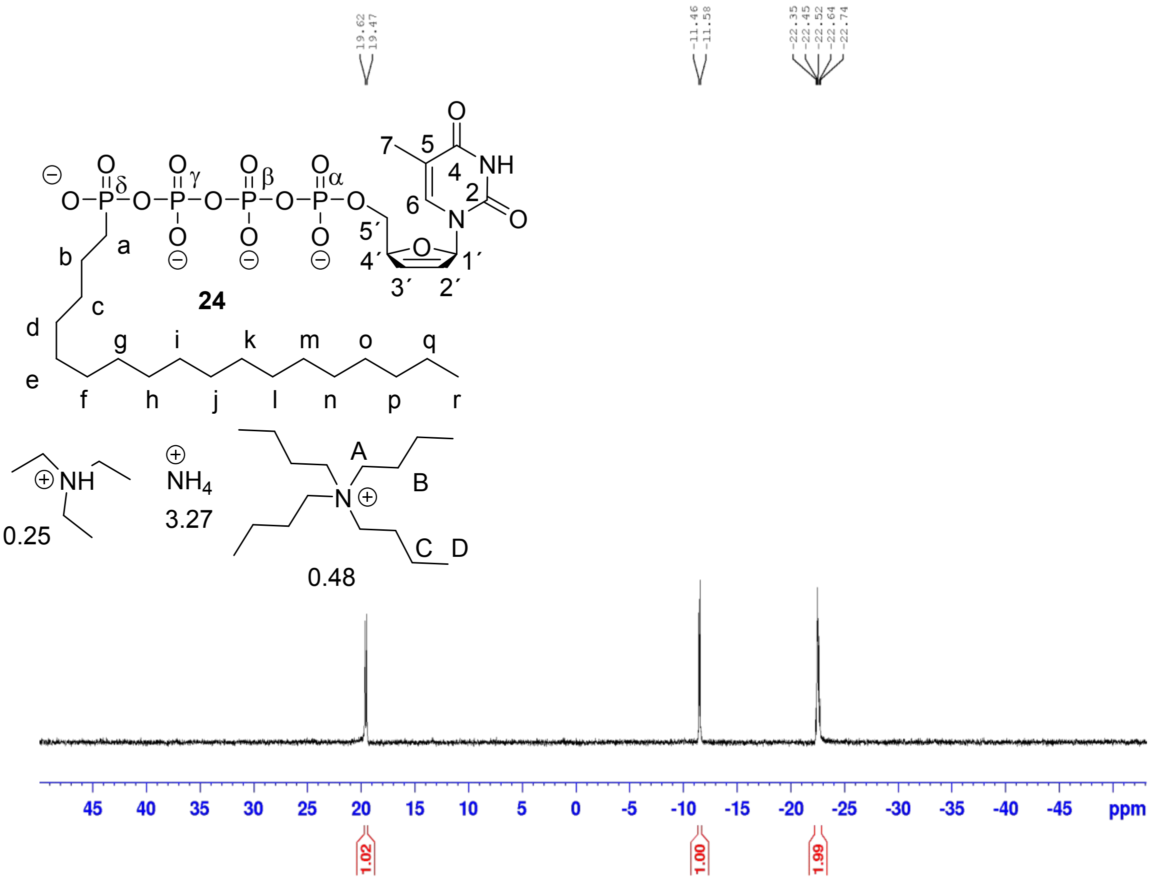
19.62
19.47
-11.46
-11.58
-22.35
-22.45
-22.52
-22.64
-22.74
24
0.25
3.27
0.48
ppm
1.02
1.00
1.99

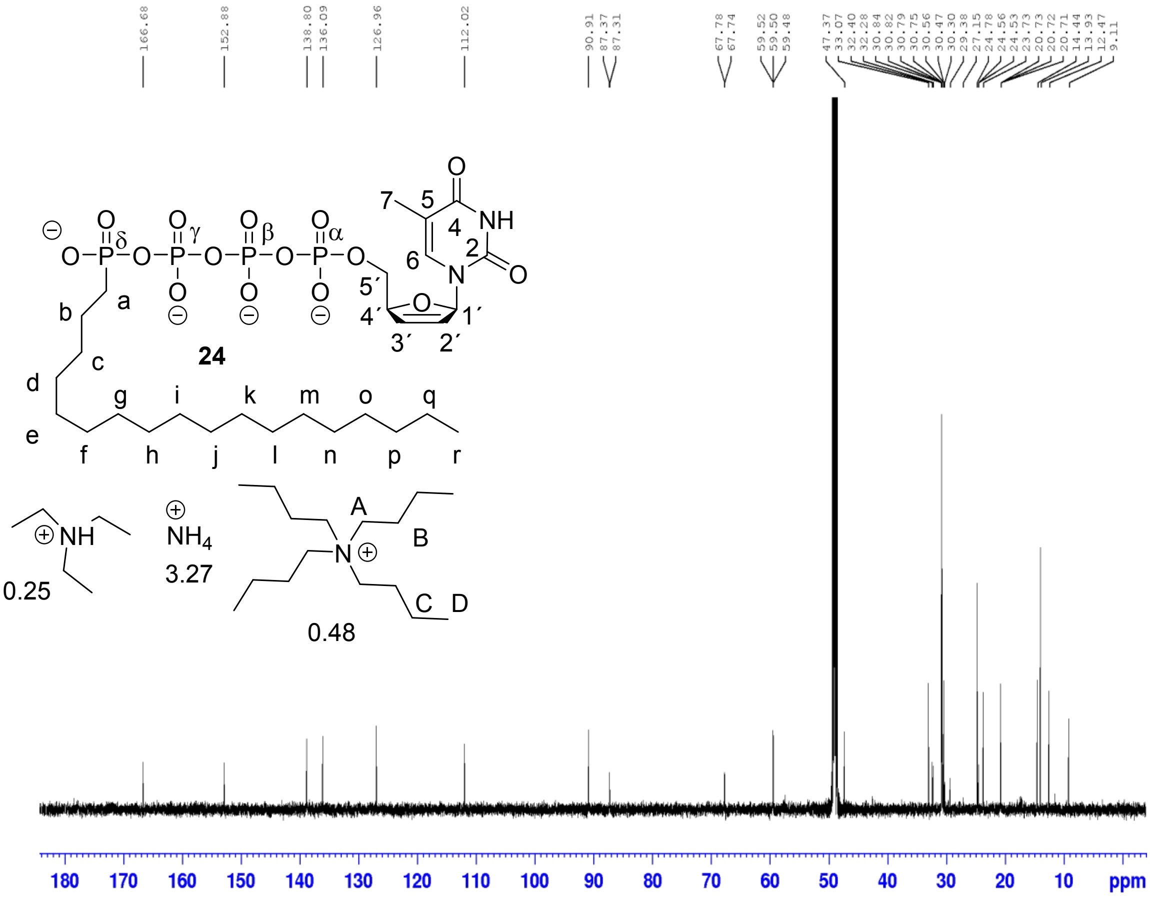
166.68
152.88
138.80
136.09
126.96
112.02
90.91
87.37
87.31
67.78
67.74
59.52
59.50
59.48
47.37
33.07
32.40
32.28
30.84
30.82
30.79
30.75
30.56
30.47
30.30
29.38
27.15
24.78
24.56
24.53
23.73
20.73
20.72
20.71
14.44
13.93
12.47
9.11
24
0.25
3.27
0.48
ppm

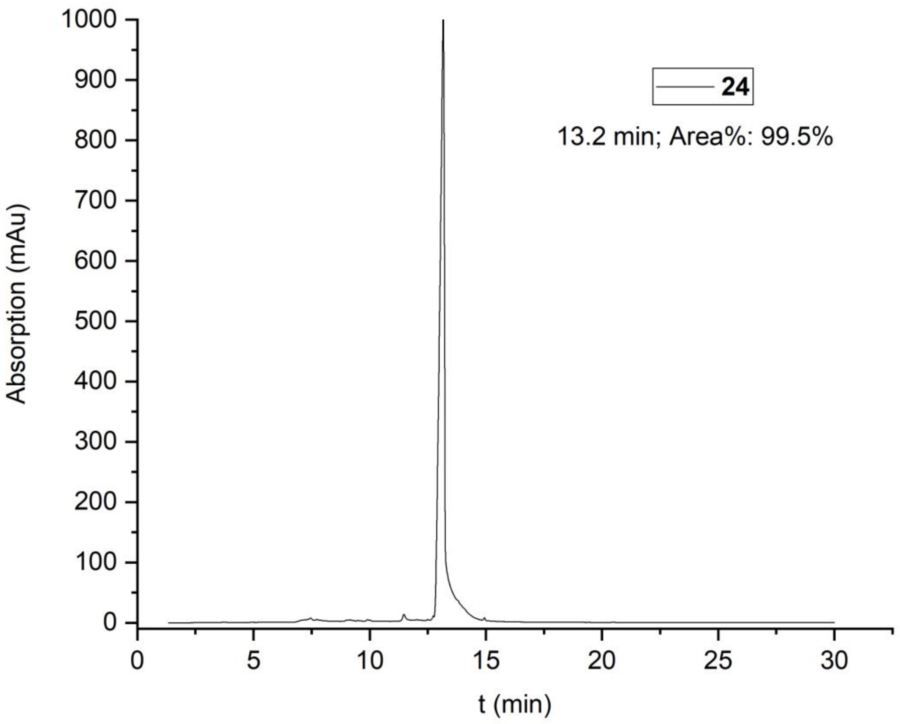

Absorption (mAu)
t (min)
24
13.2 min; Area%: 99.5%

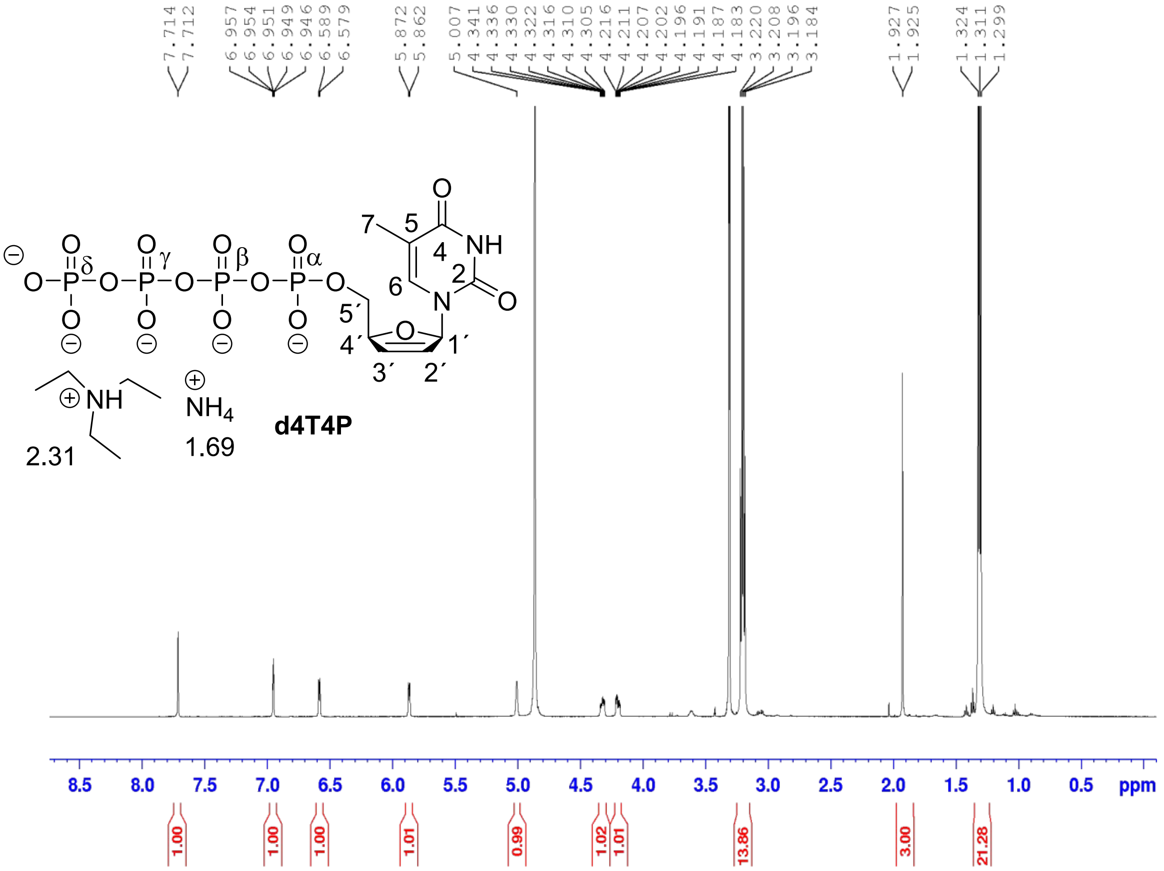

d4T4P
2.31
1.69
ppm

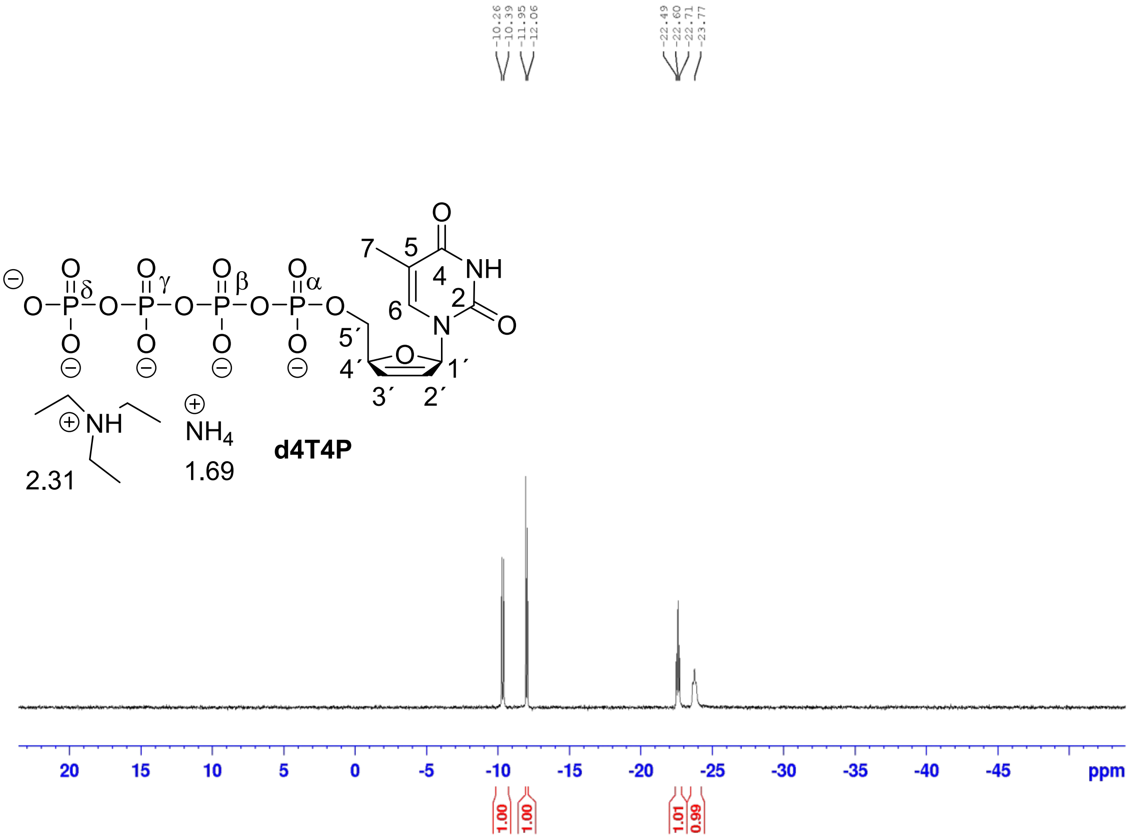

d4T4P
2.31
1.69
ppm

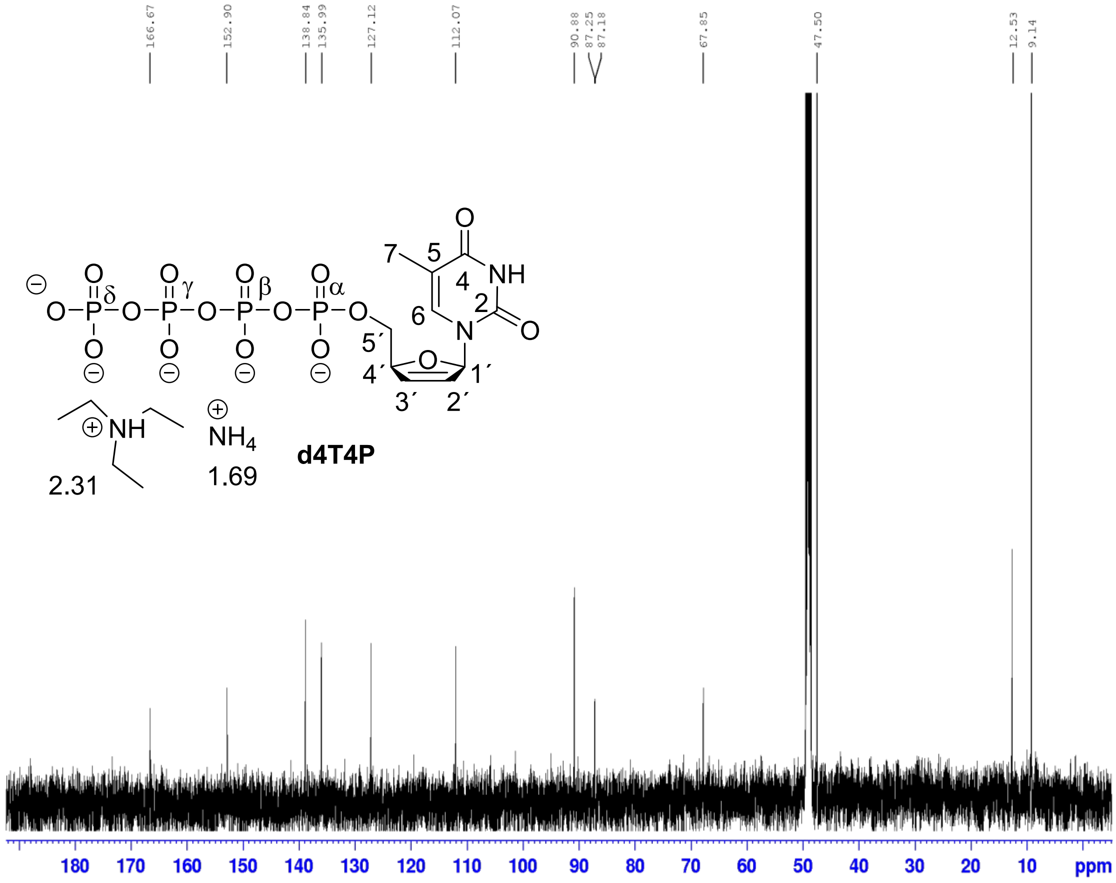

d4T4P
2.31
1.69
ppm

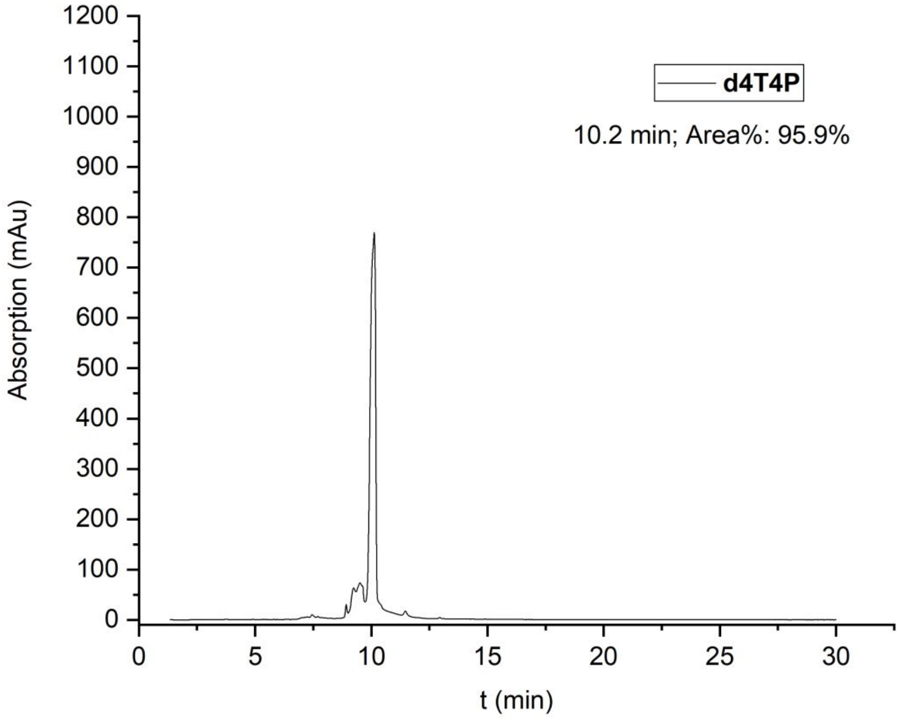

d4T4P
10.2 min; Area%: 95.9%
Absorption (mAu)
t (min)

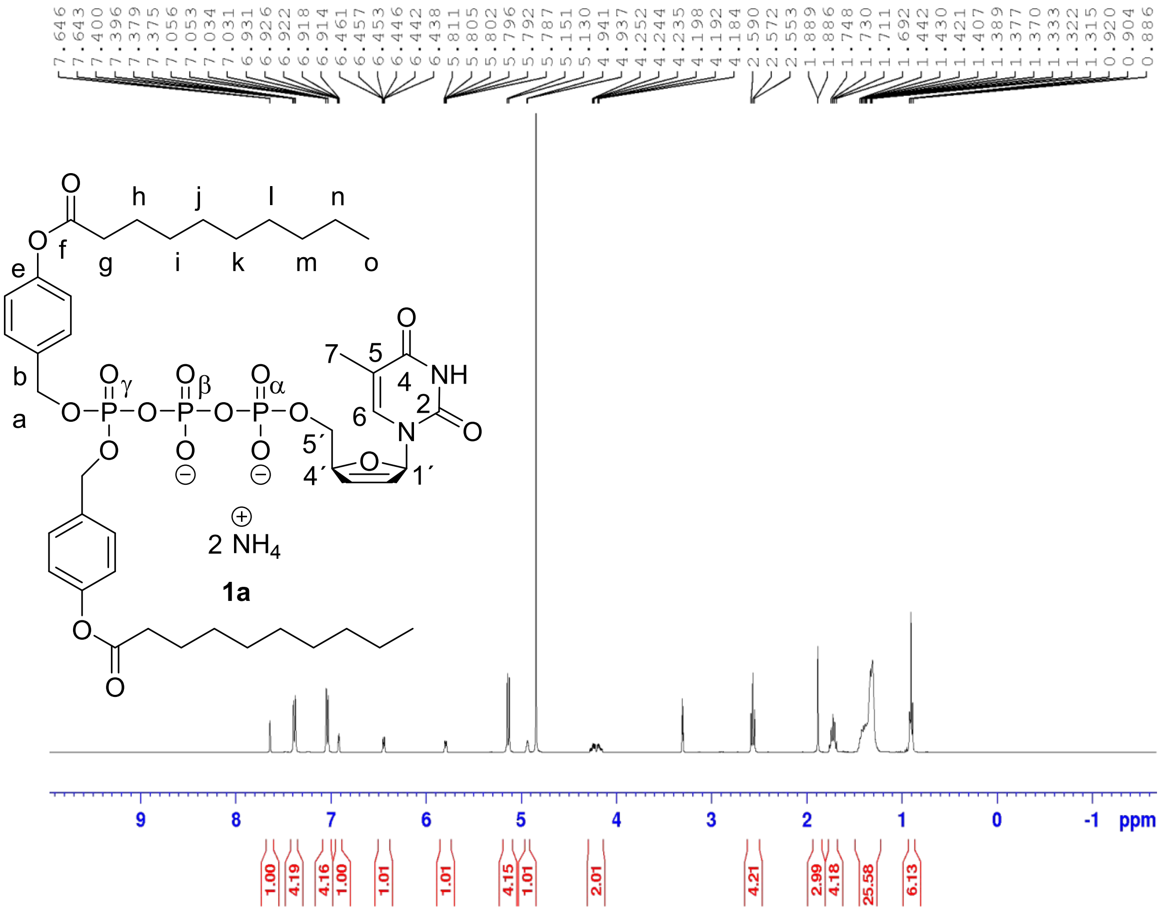

7.646 7.643 7.400 7.396 7.379 7.375 7.056 7.053 7.034 7.031 6.931 6.926 6.922 6.918 6.914 6.461 6.457 6.453 6.446 6.442 6.438 5.811 5.805 5.802 5.796 5.792 5.787 5.151 5.130 4.941 4.937 4.252 4.244 4.235 4.198 4.192 4.184 2.590 2.572 2.553 1.889 1.886 1.748 1.730 1.711 1.692 1.442 1.430 1.421 1.407 1.389 1.377 1.370 1.333 1.322 1.315 0.920 0.904 0.886
2 NH4
1a
ppm
1.00 4.19 4.16 1.00 1.01 1.01 4.15 1.01 2.01 4.21 2.99 4.18 25.58 6.13

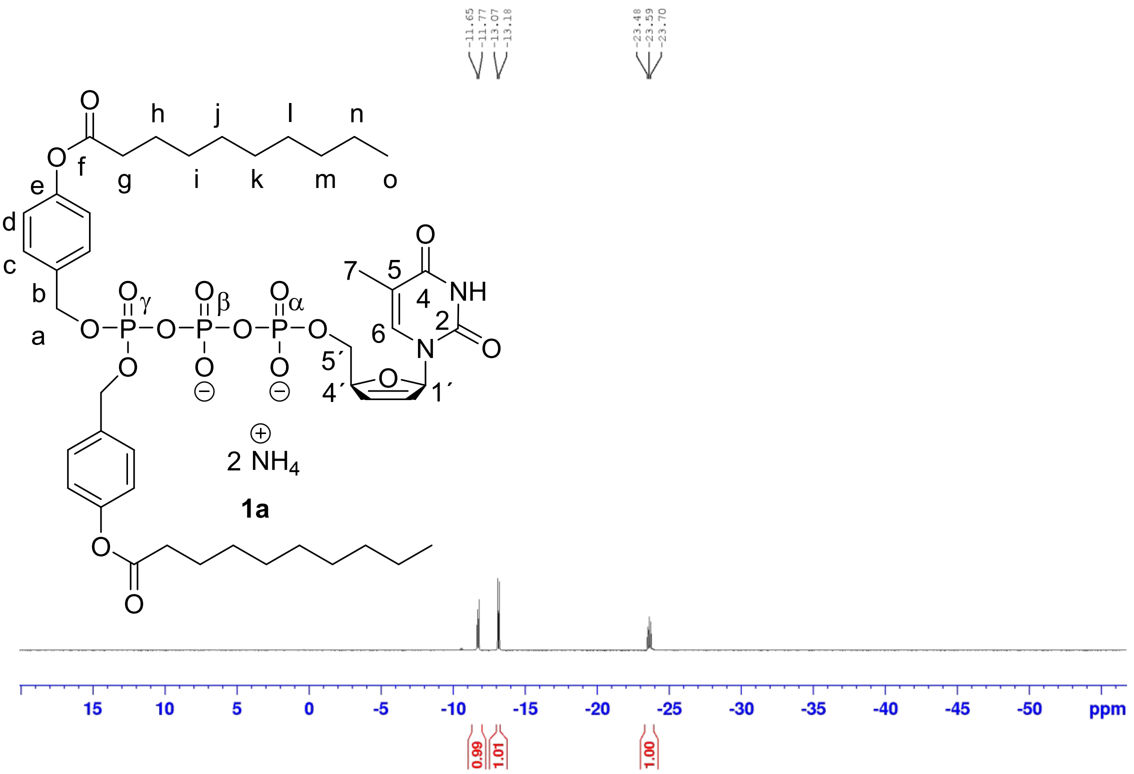
-11.65
-11.77
-13.07
-13.18
-23.48
-23.59
-23.70
O
h
j
l
n
O
f
g
i
k
m
o
e
d
c
b
a
7
5
4
NH
2
6
N
O
O
γ
β
α
5´
4´
1´
2 NH4
1a
0.99
1.01
1.00
ppm

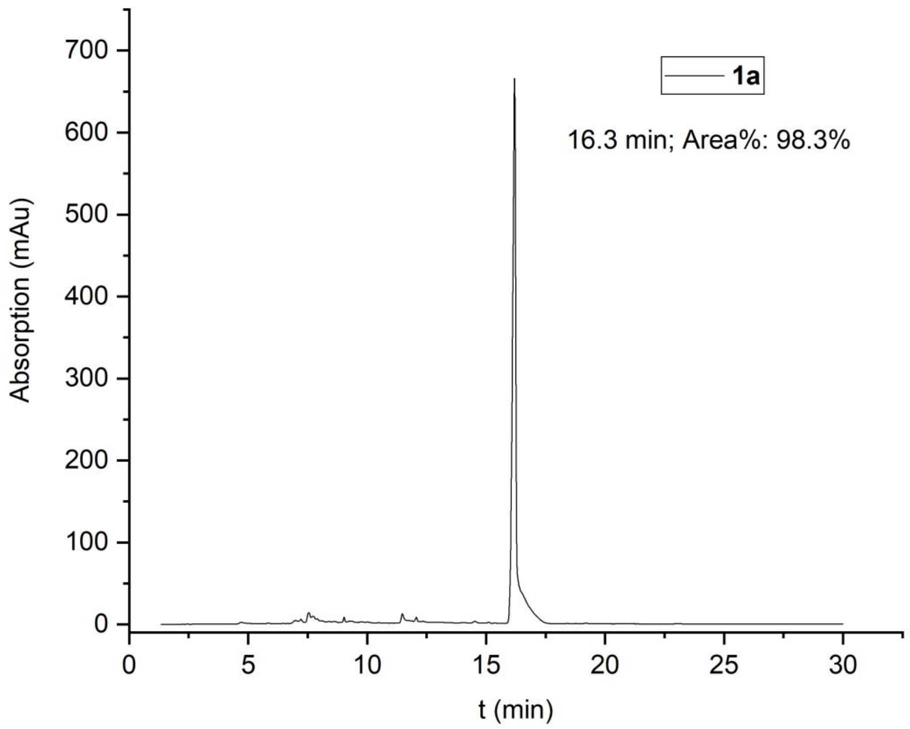
1a
16.3 min; Area%: 98.3%
Absorption (mAu)
t (min)
